# Society for Cardiovascular Magnetic Resonance reference values (“normal values”) in cardiovascular magnetic resonance: 2025 update

**DOI:** 10.1016/j.jocmr.2025.101853

**Published:** 2025-02-04

**Authors:** Nadine Kawel-Boehm, Scott J. Hetzel, Bharath Ambale-Venkatesh, Gabriella Captur, Calvin W.L. Chin, Christopher J. François, Michael Jerosch-Herold, Judy M. Luu, Zahra Raisi-Estabragh, Jitka Starekova, Michael Taylor, Max van Hout, David A. Bluemke

**Affiliations:** a Department of Radiology, Kantonsspital Graubuenden, Chur, Switzerland; b Department of Biostatistics and Medical Informatics, University of Wisconsin, Madison, Wisconsin, USA; c Department of Radiology, Johns Hopkins University, Baltimore, Maryland, USA; d Inherited Heart Muscle Conditions Clinic, Royal Free Hospital NHS Foundation Trust, London, UK; e Institute of Cardiovascular Science, University College London, London, UK; f Department of Cardiology, National Heart Centre, Singapore, Singapore; g Cardiovascular Sciences ACP, Duke NUS Medical School, Singapore, Singapore; h Department of Radiology, Mayo Clinic, Rochester, Minnesota, USA; i Department of Radiology, Brigham and Women’s Hospital, Boston, Massachusetts, USA; j Department of Medicine, Division of Cardiology, McGill University Health Centre, Montreal, Quebec, Canada; k William Harvey Research Institute, NIHR Barts Biomedical Research Centre, Queen Mary University of London, London, UK; l Department of Radiology, University of Wisconsin, Madison, Wisconsin, USA; m Dell Children's Hospital Medical Center, University of Texas Dell Medical School, Austin, Texas, USA; n Department of Cardiology, Leiden University Medical Center, Leiden, the Netherlands

**Keywords:** Normal values, Reference range, Cardiovascular magnetic resonance

## Abstract

Quantitative assessment of morphological and functional cardiac parameters by cardiovascular magnetic resonance (CMR) is essential for research and routine clinical practice. Beyond established parameters of chamber size and function, tissue properties such as relaxation times play an increasing role. Normal reference ranges are required for interpretation of results obtained by quantitative CMR. Since the last publication of the “normal values review” in 2020 many new publications related to CMR reference values have been published, which were integrated in this update. The larger sample size provides greater statistical confidence in the estimates of upper and lower limits, and enables further partitioning, e.g., by age and ethnicity for several parameters. Previous topics were expanded by new sections.

## Graphical abstract

**Figure:**
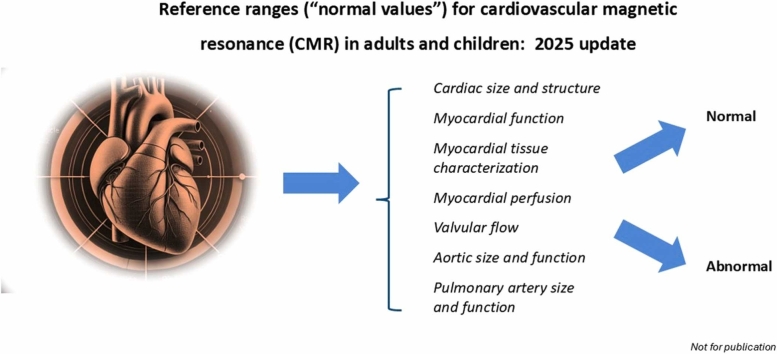

## 1 Introduction

This review provides reference ranges (“normal values”) for quantitative cardiovascular magnetic resonance imaging (CMR) including various morphologic and functional parameters. Reference ranges are required to distinguish between normal and abnormal results of quantitative CMR imaging assessment, including chamber size, regional and global function, morphologic parameters, and tissue composition, as well as vessel size and flow. Quantitative imaging further enables grading of disease severity, monitoring changes under therapy, and evaluating prognosis.

Since the last publication of the “normal value review” in 2020 [1], a recent systematic review of the literature revealed new publications related to normal reference ranges in CMR imaging, including publications with large samples of healthy volunteers, e.g., based on a subset of healthy participants of the UK-Biobank cohort. The larger sample size provides greater statistical confidence in the estimates of upper and lower limits, and enables further partitioning by age, gender, and ethnicity for several parameters. Further, previous topics were expanded by new sections including parametric mapping and strain in children and quantification of myocardial fat.

## 2 Methods

For each section/parameter, a separate literature search for publications including reference ranges of a healthy cohort was performed in PubMed. Inclusion criteria were as follows:

a) Sample size of at least 40 subjects. This is the smallest sample size that allows calculation of reference ranges using a parametric method for data with a Gaussian distribution [2]. In circumstances where gender-specific reference ranges were required, calculated reference ranges were limited to studies with at least 40 subjects per gender. Exceptions to sample size of 40 subjects per group were made in cases where no publication was available with sufficient sample size for a certain parameter. However, reference ranges based on a smaller sample size are of limited validity and should be applied with caution.
b) Only values of “healthy” reference cohorts were included. In particular, reference cohorts that included subjects with a disease or condition known to affect the measured parameter (e.g., hypertension and diabetes) were excluded. For publications that described population statistics (e.g., the Multi-Ethnic Study in Atherosclerosis [MESA] study, UK Biobank), we used data only from subgroups of individuals without risk factors or conditions known to affect the CMR parameter. In cases where the original manuscript did not provide sufficient information to allow upper and lower limits to be calculated, authors were contacted to clarify their inclusion and exclusion criteria.
c) If two or more publications were determined to refer to the same healthy reference cohort, the values of the cohort were included only once.

Manuscripts were then excluded from consideration as follows: a) obsolescent CMR technique, b) missing data that were not provided by the authors of the original publication on request, c) insufficient or inconsistent description of methods, and/or d) methods of analysis that were not consistent with current Society for Cardiovascular Magnetic Resonance (SCMR) guidelines [3] as of the time of this review.

Each quantitative CMR parameter is discussed in a separate section, with separate sections for adults, children and athletes. Each section follows the same structure. The first table of each section lists all publications eligible for inclusion providing information about the sample size, male-to-female ratio, age range and study population. Further, CMR acquisition parameters are provided. Information regarding post-processing are also included in the first table of each section and measurement technique is illustrated in figures. The first table is followed by a paragraph discussing factors with a potential influence on reference values (e.g., age, gender, anthropometric measures, ethnicity, differences in imaging technique and post-processing). This paragraph is followed by tables providing the mean value, standard deviation and reference range of the respective parameter. When statistically feasible, weighted mean values were calculated based on several studies using the same acquisition and measurement technique. In cases when the combination of studies was not possible, reference ranges of the studies are shown separately.

### 2.1 Statistical methods

Statistical analyses were performed with R for statistical computing (version 4.1, R Core Team, Vienna, Austria [4]). Results from multiple studies reporting normal values for the same CMR parameters were combined using a random effects meta-analysis model as implemented by the metamean function in the meta library in R. This produced a weighted, pooled estimate of the population mean of the CMR parameters in the combined studies. Reference ranges were estimated as the 0.025th and the 0.975th quantile using the Frequentist approach centered around a random effects model as described recently by Siegel et al for aggregated participant data [5].

In several cases, it was not possible to calculate weighted mean values based on a combination of different publications, related to differences in image acquisition and/or measurement technique, differences in cohorts (e.g., ethnic variation), and large differences in mean values for unknown reasons (leading to large standard deviations of the weighted mean and thus to unrealistic references ranges). In these cases, mean and standard deviation and reference ranges are reported separately for each study. Studies related to a very specific reference group and/or a postprocessing technique differing from the standard techniques indicated by the SCMR guidelines for post-processing in CMR were referenced only [3].

Reference ranges for single studies were calculated as the mean plus or minus twice the standard deviation. In cases with a small sample size (<40 subjects) or a large standard deviation with consecutively unrealistic lower and upper limits (e.g., negative values), reference ranges were not calculated and only the mean and standard deviation were provided instead.

Reference limits were rounded up to avoid excess digits beyond the measurement capability of CMR.

## 3 Normal volumes, mass, dimensions, and function of the left ventricle in adults

Figs. 1 and 2

**Fig. 1:**
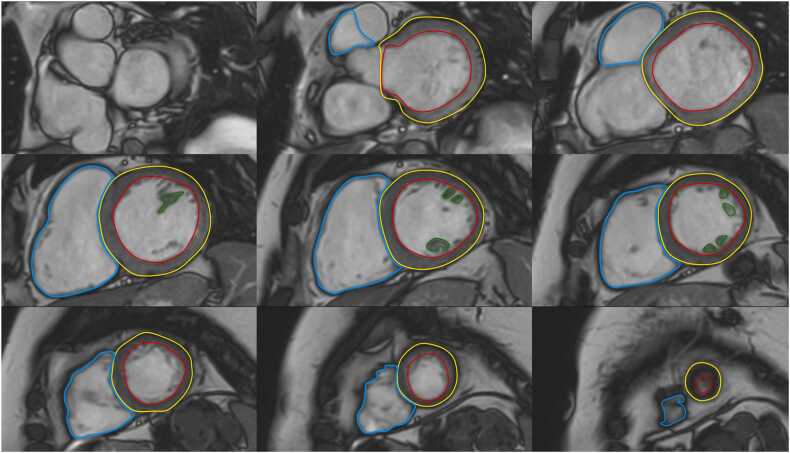
Contouring of the left and right ventricle. Note that left ventricular papillary muscle mass has been isolated and added to left ventricular mass. Right ventricular papillary muscles and trabeculations were included in the right ventricular volume

**Fig. 2:**
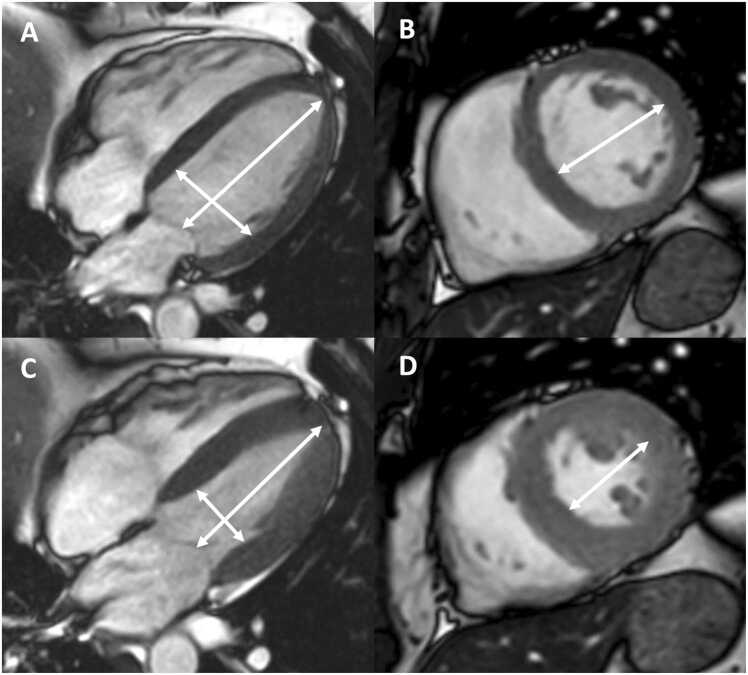
Measurements of left ventricular diameters obtained on cine balanced steady-state free precession images during diastole (A, B) and systole (C, D) on the four-chamber view (A, C) and short-axis view (B, D). The longitudinal diameter of the left ventricle was measured on the four-chamber view as the distance between the mitral valve plane and left ventricular apex (A, C). On the four-chamber view, the transverse diameter was defined as the distance between the septum and the lateral wall at the basal level. On the short-axis view, the transverse diameter was obtained at the level of the basal papillary muscles (B, D)

### 3.1 Influencing factors

**Gender:** There are significant differences in left ventricular (LV) metrics between men and women. On average, men have higher absolute LV volumes and mass compared to women [28]. These differences persist after indexing for body size [6].

**Table 1:** References. Normal volumes, mass, dimensions, and function of the left ventricle in adults.

First author, year n, male: female Age range Study population	CMR acquisition	Parameter	Post-processing (Figs. 1 and 2)
Hudsmith, 2005 [6] 63:45 21-68 years Volunteers, UK	• 1.5T, cine bSSFP • Short-axis stack	• LVEF, LVM, LVEDV, LVESV and LVSV • Absolute and indexed to BSA	• Argus software (Version 2002B^a^). Manual tracing of the endocardial and epicardial contour at end-diastole and end-systole. • Basal slice defined as the slice where 50% of the blood volume was surrounded by myocardium. • Apical slice defined as the last slice showing intracavity blood pool. • Papillary muscles were included in LVM and excluded from LV volume.
Maceira, 2006 [7] 60:60 20-80 years Volunteers, UK	• 1.5T, cine bSSFP • Short-axis stack, 2ch, 4ch	• LVEF, LVM, LVEDV, LVESV and LVSV • Absolute and indexed to BSA • Early and active peak filling rate, absolute and indexed to BSA • Septal and lateral atrioventricular plane descent • Sphericity index at diastole and systole	• Semiautomatic software used for contouring (CMRtools^b^) of endocardial and epicardial borders in all planes in all cardiac phases. • Papillary muscles were included in LVM and excluded from LV volume using thresholding technique. • Peak filling rate was calculated from the derivative of the time/volume curve. • Longitudinal atrioventricular plane descent was measured in the septum and lateral wall and expressed as a ratio of the ventricular length. • Sphericity index calculated as LVEDV/(4π/3)(4ch length/2)^3^.
Chang, 2012 [8] 64:60 20-70 years Volunteers, Korea	• 1.5T, cine bSSFP • Short-axis stack	• LVEF, LVM, LVEDV, LVESV, LVSV, LVCO. • Absolute and indexed to BSA	• Manual contouring at end-systole and end-diastole using Argus software (version 4.02).^a^ • Basal slice defined as the slice where 50% of the blood volume was surrounded by myocardium. • Papillary muscles and trabeculae were excluded from the LVM and included in the LV volume.
Macedo, 2013 [9] 54:53 20-80 years Substudy of CMR-LAC registry, Brazil	• 1.5T, cine bSSFP • Short-axis stack, 2ch, 4ch	• LVEF, LVM, LVEDV, LVESV, EDD, ESD. • Absolute and indexed to BSA	• Semi-automatic contouring at end-systole and end-diastole using View Forum^c^ and Argus software.^a^ • Basal slice defined as the slice where 50% of the blood volume was surrounded by myocardium. • Apical slice defined as the last slice showing intracavity blood pool. • Papillary muscles were included in LVM and excluded from LV volume.
Yeon, 2015 [10] 340:512 (61 ± 9)^d^ years FHS offspring cohort, USA	• 1.5T, cine bSSFP • Short-axis stack	• LVEF, LVM, LVEDV, LVESV, EDD, LVM/EDV • Absolute, indexed to BSA, indexed to height, indexed to allometric powers of height (HT^1.7^ and HT^2.7^), and indexed to fat-free-mass	• Manual contouring at end-systole and end-diastole using the EasyVision software (version 5.1).^c^
Le Ven, 2016 [11] 196:238 18-36 years Caucasian volunteers, Canada	• 1.5T, cine bSSFP • Short-axis stack, 2ch, 4ch, 3ch	• LVEDV, LVESV, LVSV, LVEF, LVM • Segmental systolic wall thickening • Absolute and indexed to BSA.	• Semi-automated segmentation in end-diastole and end-systole using cmr42 software (version 3.4.1)^e^ • Papillary muscles were included in LVM and excluded from LV volume. • If the basal slice contained both ventricular and atrial wall, the contours were drawn up to the junction of the atrium and the ventricle and the appropriate volume attributed to the ventricle. • If the aortic valve appeared in the basal slice, blood volume up to the aortic valve was included in the LV volume.
Lei, 2017 [12] 60:60 23-83 years Volunteers, China	• 3.0T, bSSFP • Short-axis stack, 2ch, 4ch, 3ch	• LVEDV, LVESV, LVSV, LVCO, LVM, LVEF, EDD, ESD. • Absolute and indexed to BSA.	• Manual analysis in end-diastole and end-systole using Qmass software (version 7.6).^f^ • Papillary muscles were excluded from LVM and included in LV volume. • Contours were drawn up to the aortic valve cusp with the outflow tract included in the LV volume.
Bentatou, 2018 [13] 70:70 20-69 years Caucasian volunteers, France	• 1.5T, bSSFP • Short-axis stack	• LVEDV, LVESV, LVM, LVEF • Compact (C) myocardial mass, trabeculated (T) myocardial mass, T/C ratio • Indexed to BSA	• Manual segmentation in end-diastole and end-systole using Argus software.^a^ • Basal slice defined as the slice where 50% of the blood volume was surrounded by myocardium. • LV trabeculations, endocardial and epicardial borders were defined, in the whole stack of the diastolic phase, using the semi-automatic method of Bricq et al. [14] • Compact and trabeculated myocardial masses were defined as the difference between the epicardium and the endocardium and between the trabeculation contour and the endocardium, respectively. • Papillary muscles were included in the compact LVM.
Liu, 2018 [15] 50:50 20-70 years Volunteers, UK	• 1.5T, cine bSSFP • Short-axis stack, 2ch, 4ch	• LVEF, LVEDV, LVESV, LVM • Indexed to BSA	• Manual segmentation in end-diastole and end-systole using cvi42 software (version 5.3.4).^e^ • Delineation of papillary muscles and trabeculations using thresholding • Papillary muscles were included in LVM and excluded from LV volume.
Zhuang, 2020 [16] 100:100 20-70 years Volunteers, China	• 3.0T, cine bSSFP • Short-axis stack, 2ch, 4ch	• LVEDD (horizontal, vertical) • (LV area, LVEF, LVEDV, LVESV, LVSV, LVM, LVCO; not included, LV contouring method not described) • Absolute and indexed to BSA	• LV areas measured at end-diastole in four-chamber view. • LV dimensions (measured parallel to the interventricular septum) in the maximum short-axis view at both end- diastole and end-systole. • Semiautomated segmentation in end-diastole and end-systole using cvi42 software.^e^ • Basal slice defined as the slice where 50% of the blood volume was surrounded by myocardium.
Gregor, 2021 [17] 100:100 20->50 years Volunteers, Hungary	• 1.5T, cine bSSFP • Short-axis stack, 2ch, 4ch, 3ch	• LVEDV, LVESV, LVSV, LVCO, LVCI, LVEF, total LV mass • LV compact myocardial mass, total papillary muscle mass, trabeculated and papillary muscle mass-to-myocardial mass, myocardial mass-to-end-diastolic volume, trabeculated and papillary muscle mass-to-end-diastolic volume ratios • Indexed to BSA	• Semi-automatic segmentation in end-diastole and end-systole using Medis Suite software (version 3.20).^f^ • Threshold-based method (MassK module of the Medis Suite program^f^) was used to delineate endocardial and papillary muscles. • Present results for both total LVM (papillary muscles included in LVM) and compact LVM (papillary muscles excluded from LVM).
Meloni, 2021[18] 50:50 20-70 years Volunteers, Italy	• 1.5T, cine bSSFP • Short-axis stack, 2ch, 4ch	• LVEDV, LVESV, LVM, LVSV, LVEF • Indexed to BSA	• Manual segmentation in end-diastole and end-systole using cvi42 software (version 5.11.4).^e^ • Papillary muscles were included in LVM and excluded from LV volume.
Zhang, 2021 [19] 323:227 21-70 years Screening cohort, China	• 1.5T, cine bSSFP • Short-axis stack, 2ch, 4ch, 3ch	• LVEDV, LVESV, LVSV, LVEF, LVM, LVCO. • Absolute and indexed to BSA	• Semi-automatic segmentation in end-diastole and end-systole using cvi42 software (version 5.6.2).^e^ • Basal slice defined as the slice where 50% of the blood volume was surrounded by myocardium. • The most apical section with visible cavity was considered as the apex. • LV endocardial borders were traced by semi-automatic threshold-based segmentation method with manual correction if necessary. • Trabeculae and papillary muscles were excluded from the LV blood pool and included in the LVM.
Luu, 2022 [20] 1126:2080 35-75 years CAHHM, Canada	• 1.5T and 3.0T, cine bSSFP • Short-axis stack, 2ch, 4ch	• LVEF, LVSV, LVEDV, LVESV, LVM, LVM/ LVEDV • Indexed to BSA	• Segmentation in end-diastole and end-systole using cvi42 software (version 4).^e^ • Epicardial and endocardial contours were traced manually or semi-automatically using the built-in threshold tool. • Papillary muscles and trabecular tissue were included in LVM, measured at end-systole. • For LV volume calculations, the entire outflow tract was included into the end-diastolic and end-systolic phases. The trabeculae and papillary muscles were excluded from the blood pool.
Raisi-Estabragh, 2024 [21] 4452:4636 18-83 years Healthy Hearts Consortium^g^	• 1.5T and 3.0T, cine bSSFP • Short-axis stack, 2ch, 4ch, 3ch	• LVEDV, LVESV, LVSV, LVCO, LVEF, LVM • Absolute, indexed to BSA, and indexed to height.	• Fully automated batch processing using the cvi42 software (prototype version 5.14).^e^ • Results presented for three segmentation methods: 1) smooth: trabecular and papillary muscles excluded from LVM and included in blood pool, 2) papillary: papillary muscles included in LVM and excluded from the blood pool, 3) anatomical: papillary muscles and trabecular included in LVM and excluded from blood pool.

*n* number of study subjects, *T* Tesla, *bSSFP* balanced steady-state free precession, *LV* left ventricular, *EF* ejection fraction, *M* mass, *EDV* end-diastolic volume, *ESV* end-systolic volume, *SV* stroke volume, *BSA* body surface area, *2ch* two-chamber view, *4ch* four-chamber view, *CO* cardiac output, *CI* cardiac index, *CMR-LAC registry* The Latin-American Multi-Center reference study of CMR, *EDD* end-diastolic diameter, *ESD* end-systolic diameter, *3ch* three-chamber view, *FHS* Framingham Heart Study, *CAHHM* Canadian Alliance for Healthy Heart and Minds

^a^Siemens Healthineers AG, Erlangen, Germany; ^b^Cardiovascular Imaging Solutions, London, UK; ^c^Koninklijke Philips N.V., Amsterdam, The Netherlands; ^d^ mean ± SD (age range not provided in original publication); ^e^Circle Cardiovascular Imaging Inc., Calgary, Alberta, Canada; ^f^Medis Medical Imaging Systems, Leiden, The Netherlands; ^g^UK Biobank, UK (n = 7672) [22] + SHIP, Germany (Study of Health in Pomerania) (n = 627) [23] + Italy (n = 291) [24] + Germany (n = 190) [25] + Singapore (n = 180) [26] + The Netherlands (n = 128) [27]

**Table 2:** Absolute and indexed (BSA) parameters of left ventricular volume, mass, and function in men and women for bSSFP imaging; papillary muscles/trabeculations included in left ventricular mass.^a^

		Men			Women		
Parameter	References	n	Mean ± SD	LL-UL	n	Mean ± SD	LL-UL
LVEDV (mL)	[6], [7], [9], [11], [19], [20], [21]	6213	147 ± 33	83-211	7269	118 ± 24	72-164
LVEDV/BSA (mL/m^2^)	[6], [7], [9], [11], [13], [15], [17], [18], [19], [20], [21]	6485	75 ± 15	46-104	7538	69 ± 11	46-91
LVESV (mL)	[6], [7], [9], [11], [19], [20], [21]	6206	51 ± 16	20-82	7264	39 ± 11	17-60
LVESV/BSA (mL/m^2^)	[6], [7], [9], [11], [13], [15], [17], [18], [19], [20], [21]	6471	26 ± 8	11-41	7538	23 ± 6	11-34
LVSV (mL)	[6], [7], [11], [19], [20], [21]	6158	98 ± 23	53-142	7214	79 ± 16	47-111
LVSV/BSA (mL/m^2^)	[6], [7], [11], [17], [18], [19], [20], [21]	6312	50 ± 10	30-69	7372	47 ± 8	31-63
LVEF (%)	[6], [7], [9], [11], [13], [15], [17], [18], [19], [20], [21]	6472	66 ± 7	53-79	7541	67 ± 6	55-80
LVM (g)	[6], [7], [9], [11], [19], [20], [21]	6205	124 ± 23	78-169	7261	87 ± 18	52-123
LVM/BSA (g/m^2^)	[6], [7], [9], [11], [15], [17], [18], [19], [20], [21]	6374	64 ± 10	44-84	7463	52 ± 9	35-69
LVCO (L/min)	[19], [21]	4719	5.5 ± 1.4	2.6-8.3	4794	4.7 ± 1.1	2.6-6.9
LVCI (L/min/m^2^)	[17]	100	3.2 ± 0.8	1.6-4.8	100	3.0^b^	[2.6,3.6]^c^
LVM/LVEDV (g/mL)	[11], [20]	1310	0.8 ± 0.2	0.5-1.1	2305	0.7 ± 0.1	0.4-1.0

*Data are means ± standard deviation or median and interquartile range, as indicated*. *BSA* body surface area, *bSSFP* balanced steady-state free precession, *n* number of study subjects, *SD* standard deviation, *LL* lower limit, *UL* upper limit, *LV* left ventricular, *EDV* end-diastolic volume, *ESV* end-systolic volume, *SV* stroke volume, *EF* ejection fraction, *M* mass, *CO* cardiac output, *CI* cardiac index

^a^Measured on a short-axis cine stack, ^b^median, ^c^[interquartile range] (according to original publication). References [20,21] provided additional data for analysis

**Table 3:** Absolute and indexed (BSA) parameters of left ventricular volume, mass, and function in men and women for bSSFP imaging; papillary muscles/trabeculations included in left ventricular volume.^a^

		Men			Women		
Parameter	References	n	Mean ± SD	LL-UL	n	Mean ± SD	LL-UL
LVEDV (mL)	[8], [10], [12], [21]	4867	143 ± 31	81-204	5213	113 ± 23	68-158
LVEDV/BSA (mL/m^2^)	[8], [10], [12], [21]	4868	75 ± 14	49-102	5209	68 ± 11	46-90
LVESV (mL)	[8], [10], [12], [21]	4857	52 ± 18	17-88	5203	39 ± 13	14-63
LVESV/BSA (mL/m^2^)	[8], [10], [12], [21]	4847	28 ± 8	11-44	5200	23 ± 6	11-36
LVSV (mL)	[8], [10], [12], [21]	4867	91 ± 19	53-128	5212	74 ± 15	45-103
LVSV/BSA (mL/m^2^)	[8], [10], [21]	4805	47 ± 9	30-64	5159	44 ± 8	29-60
LVEF (%)	[8], [10], [12], [21]	4854	64 ± 7	51-77	5209	66 ± 6	54-79
LVM (g)	[8], [10], [12], [21]	4859	105 ± 23	60-150	5203	74 ± 14	47-101
LVM/BSA (g/m^2^)	[8], [10], [12], [21]	4850	55 ± 8	39-72	5205	45 ± 6	33-56
LVCO (L/min)	[8], [10], [12], [21]	4856	6.1 ± 1.3	3.6-8.6	5199	4.9 ± 1.1	2.9-7.0
LVCI (L/min/m^2^)	[8], [10]	404	3.2 ± 0.6	1.9-4.4	572	3.0 ± 0.6	1.8-4.2
LVM/LVEDV (g/mL)	[10]	340	0.9 ± 0.2	0.5-1.3	512	0.8 ± 0.1	0.6-1.0

*Data are means ± standard deviation*. *BSA* body surface area, *bSSFP* balanced steady-state free precession, *n* number of study subjects, *SD* standard deviation, *LL* lower limit, *UL* upper limit, *LV* left ventricular, *EDV* end-diastolic volume, *ESV* end-systolic volume, *SV* stroke volume, *EF* ejection fraction, *M* mass, *CO* cardiac output, *CI* cardiac index

^a^Measured on a short-axis cine stack

References [8], [21] provided additional data for analysis

**Table 4:** Absolute and indexed (BSA) parameters of left ventricular volume, mass, and function in men and women by ethnicity for bSSFP imaging; papillary muscles/trabeculation included in left ventricular mass.^a^

			Men			Women		
Parameter	Ethnicity	References	n	Mean ± SD	LL-UL	n	Mean ± SD	LL-UL
LVEDV (mL)	Black	[21]	148	146 ± 28	90-201	189	118 ± 21	76-159
	Caucasian	[11], [20], [21]	4680	156 ± 31	95-217	5553	119 ± 21	78-160
	Chinese	[19], [20], [21]	663	122 ± 22	80-165	766	98 ± 16	67-128
	Latin American	[9]	54	142 ± 27	88-196	53	114 ± 26	62-166
	South Asian	[20], [21]	367	120 ± 22	77-163	252	98 ± 17	64-131
LVEDV/BSA (mL/m^2^)	Black	[21]	148	71 ± 13	47-96	189	64 ± 10	45-83
	Caucasian	[11], [13], [17], [18], [20], [21]	4902	77 ± 15	48-106	5776	69 ± 11	47-91
	Chinese	[19], [20], [21]	663	67 ± 11	45-88	765	62 ± 9	45-79
	Latin American	[9]	54	74 ± 13	48-100	53	69 ± 17	35-103
	South Asian	[20], [21]	367	62 ± 11	40-83	250	58 ± 9	40-75
LVESV (mL)	Black	[21]	147	49 ± 15	19-79	189	38 ± 10	18-58
	Caucasian	[11], [20], [21]	4673	56 ± 16	25-87	5549	40 ± 11	20-61
	Chinese	[19], [20], [21]	663	43 ± 12	19-66	766	31 ± 8	14-47
	Latin American	[9]	54	50 ± 14	22-78	53	40 ± 18	4-76
	South Asian	[20], [21]	368	42 ± 12	19-64	250	32 ± 9	14-50
LVESV/BSA (mL/m^2^)	Black	[21]	146	24 ± 7	10-37	189	21 ± 5	10-31
	Caucasian	[11], [13], [17], [18], [20], [21]	4888	28 ± 8	13-43	5773	23 ± 6	11-35
	Chinese	[19], [20], [21]	663	23 ± 6	11-36	766	20 ± 5	10-29
	Latin American	[9]	54	26 ± 7	12-40	53	24 ± 11	2-46
	South Asian	[20], [21]	367	21 ± 6	11-32	250	19 ± 5	8-30
LVSV (mL)	Black	[21]	147	95 ± 18	60-130	188	79 ± 15	50-108
	Caucasian	[11], [20], [21]	4681	100 ± 21	59-142	5552	79 ± 15	50-107
	Chinese	[19], [20], [21]	663	79 ± 14	52-107	767	67 ± 12	44-90
	Latin American	-^b^	-^b^	-^b^	-^b^	-^b^	-^b^	-^b^
	South Asian	[20], [21]	366	77 ± 17	45-110	251	64 ± 13	39-90
LVSV/BSA (mL/m^2^)	Black	[21]	147	47 ± 8	31-62	189	43 ± 7	30-57
	Caucasian	[11], [17], [18], [20], [21]	4836	50 ± 10	30-69	5710	46 ± 8	31-61
	Chinese	[19], [20], [21]	663	43 ± 7	29-57	767	43 ± 7	30-56
	Latin American	-^b^	-^b^	-^b^	-^b^	-^b^	-^b^	-^b^
	South Asian	[20], [21]	364	40 ± 8	24-56	251	38 ± 7	25-52
LVEF (%)	Black	[21]	147	66 ± 6	54-79	190	68 ± 6	56-79
	Caucasian	[11], [13], [17], [18], [20], [21]	4890	64 ± 7	51-77	5773	66 ± 6	54-79
	Chinese	[19], [20], [21]	663	65 ± 6	53-78	767	69 ± 6	57-81
	Latin American	[9]	54	65 ± 6	53-77	53	66 ± 7	52-80
	South Asian	[20], [21]	366	65 ± 7	51-79	251	67 ± 7	53-81
LVM (g)	Black	[21]	148	127 ± 23	82-172	188	95 ± 17	62-128
	Caucasian	[11], [20], [21]	4674	122 ± 22	79-164	5549	83 ± 15	54-112
	Chinese	[19], [20], [21]	661	110 ± 21	69-151	766	77 ± 15	48-106
	Latin American	[9]	54	115 ± 26	63-167	53	75 ± 20	35-115
	South Asian	[20], [21]	366	102 ± 19	66-138	251	72 ± 14	45-100
LVM/BSA (g/m^2^)	Black	[21]	148	62 ± 9	45-79	189	51 ± 7	38-65
	Caucasian	[11], [17], [18], [20], [21]	4794	63 ± 10	43-84	5697	50 ± 8	34-67
	Chinese	[19], [20], [21]	661	60 ± 10	40-80	766	49 ± 8	33-65
	Latin American	[9]	54	60 ± 12	36-84	53	45 ± 12	21-69
	South Asian	[20], [21]	366	53 ± 8	36-69	250	43 ± 7	29-56
LVCO (L/min)	Black	[21]	147	6.0 ± 1.2	3.6-8.4	188	5.1 ± 1.1	2.9-7.3
	Caucasian	[21]	3613	6.1 ± 1.3	3.6-8.6	3701	5.1 ± 1.0	3.1-7.1
	Chinese	[19], [21]	469	5.2 ± 1.1	3.1-7.3	415	4.5 ± 0.9	2.8-6.2
	Latin American	-^b^	-^b^	-^b^	-^b^	-^b^	-^b^	-^b^
	South Asian	[21]	318	5.2 ± 1.1	3.0-7.3	188	4.4 ± 0.9	2.6-6.2
LVCI (L/min/m^2^)	Black	-^b^	-^b^	-^b^	-^b^	-^b^	-^b^	-^b^
	Caucasian	[17]	100	3.2 ± 0.8	1.6-4.8	100	3.0^c^	[2.6,3.6]^d^
	Chinese	-^b^	-^b^	-^b^	-^b^	-^b^	-^b^	-^b^
	Latin American	-^b^	-^b^	-^b^	-^b^	-^b^	-^b^	-^b^
	South Asian	-^b^	-^b^	-^b^	-^b^	-^b^	-^b^	-^b^
LVM/LVEDV (g/mL)	Black	-^b^	-^b^	-^b^	-^b^	-^b^	-^b^	-^b^
	Caucasian	[11], [20]	1057	0.8 ± 0.2	0.5-1.1	1842	0.7 ± 0.1	0.4-1.0
	Chinese	[20]	193	0.9 ± 0.2	0.6-1.2	352	0.8 ± 0.1	0.5-1.0
	Latin American	-^b^	-^b^	-^b^	-^b^	-^b^	-^b^	-^b^
	South Asian	[20]	51	0.9 ± 0.2	0.5-1.3	63	0.7 ± 0.1	0.5-1.0

*Data are means ± standard deviation or median and interquartile range, as indicated.BSA* body surface area, *bSSFP* balanced steady-state free precession, *n* number of study subjects, *SD* standard deviation, *LL* lower limit, *UL* upper limit, *LV* left ventricular, *EDV* end-diastolic volume, *ESV* end-systolic volume, *SV* stroke volume, *EF* ejection fraction, *M* mass, *CO* cardiac output, *CI* cardiac index

^a^Measured on a short-axis cine stack, ^b^insufficient data, ^c^median, ^d^[interquartile range] (according to original publication)

[20], [21] provided additional data for analysis

**Table 5:** Absolute and indexed (BSA) parameters of left ventricular volume, mass and function in men and women of mixed ethnicity by age for bSSFP imaging; papillary muscles/trabeculation included in left ventricular mass.^a^

			Men			Women		
Parameter	Age^b^	References	n	Mean ± SD	LL-UL	n	Mean ± SD	LL-UL
LVEDV (mL)	20-29	[7], [9], [19], [21]	193	155 ± 36	85-225	178	123 ± 25	73-172
	30-39	[7], [9], [19], [20], [21]	320	146 ± 31	86-205	289	116 ± 23	71-162
	40-49	[7], [9], [19], [20], [21]	898	144 ± 32	81-207	1137	115 ± 24	68-162
	50-59	[7], [9], [19], [20], [21]	1668	137 ± 33	72-203	2242	109 ± 23	64-155
	60-69	[7], [19], [20], [21]	1427	132 ± 34	65-198	1815	105 ± 23	59-151
	≥70	[7], [20], [21]	1440	132 ± 26	81-183	1320	105 ± 19	68-142
LVEDV/BSA (mL/m^2^)	20-29	[7], [9], [17], [19], [21]	218	78 ± 15	48-108	203	71 ± 12	48-95
	30-39	[7], [9], [17], [19], [20], [21]	344	73 ± 13	48-98	314	69 ± 12	46-92
	40-49	[7], [9], [17], [19], [20], [21]	924	74 ± 14	46-101	1163	67 ± 11	46-89
	50-59	[7], [9], [19], [20], [21]	1668	70 ± 15	41-99	2248	63 ± 11	41-86
	60-69	[7], [19], [20], [21]	1431	68 ± 15	39-98	1812	62 ± 11	40-84
	≥70	[7], [20], [21]	1438	70 ± 13	44-95	1315	62 ± 10	42-82
LVESV (mL)	20-29	[7], [9], [19], [21]	193	57 ± 19	20-93	178	43 ± 12	20-67
	30-39	[7], [9], [19], [20], [21]	320	51 ± 15	22-79	289	39 ± 13	14-65
	40-49	[7], [9], [19], [20], [21]	898	51 ± 17	18-84	1141	39 ± 12	15-62
	50-59	[7], [9], [19], [20], [21]	1669	48 ± 17	15-80	2241	35 ± 12	13-58
	60-69	[7], [19], [20], [21]	1421	44 ± 17	11-77	1807	33 ± 12	9-56
	≥70	[7], [20], [21]	1438	46 ± 14	19-74	1320	35 ± 10	15-55
LVESV/BSA (mL/m^2^)	20-29	[7], [9], [17], [19], [21]	218	28 ± 8	11-44	203	24 ± 6	12-36
	30-39	[7], [9], [17], [19], [20], [21]	344	25 ± 7	12-38	314	23 ± 8	8-38
	40-49	[7], [9], [17], [19], [20], [21]	921	25 ± 8	10-41	1165	22 ± 6	10-34
	50-59	[7], [9], [19], [20], [21]	1666	24 ± 8	9-40	2244	20 ± 6	8-33
	60-69	[7], [19], [20], [21]	1423	23 ± 8	7-39	1812	19 ± 6	7-31
	≥70	[7], [20], [21]	1437	24 ± 7	10-38	1317	20 ± 6	9-31
LVSV (mL)	20-29	[7], [19], [21]	183	98 ± 26	47-149	168	80 ± 18	45-115
	30-39	[7], [19], [20], [21]	308	93 ± 22	51-135	271	77 ± 18	42-111
	40-49	[7], [19], [20], [21]	890	94 ± 22	51-137	1123	78 ± 16	46-109
	50-59	[7], [19], [20], [21]	1650	90 ± 23	46-135	2234	75 ± 16	44-106
	60-69	[7], [19], [20], [21]	1426	87 ± 21	45-129	1816	72 ± 15	43-102
	≥70	[7], [20], [21]	1442	89 ± 19	52-126	1319	73 ± 14	45-100
LVSV/BSA (mL/m^2^)	20-29	[7], [17], [19], [21]	208	50 ± 11	28-72	193	48 ± 8	32-64
	30-39	[7], [17], [19], [20], [21]	332	47 ± 9	30-65	296	46 ± 8	29-62
	40-49	[7], [17], [19], [20], [21]	917	48 ± 9	30-67	1150	46 ± 8	31-61
	50-59	[7], [19], [20], [21]	1651	46 ± 10	27-66	2242	44 ± 8	29-59
	60-69	[7], [19], [20], [21]	1428	45 ± 10	27-64	1816	43 ± 7	28-57
	≥70	[7], [20], [21]	1442	46 ± 9	28-64	1317	43 ± 8	28-57
LVEF (%)	20-29	[7], [9], [17], [19], [21]	218	64 ± 7	50-78	203	66 ± 6	54-78
	30-39	[7], [9], [17], [19], [20], [21]	345	65 ± 6	54-77	314	67 ± 7	54-80
	40-49	[7], [9], [17], [19], [20], [21]	925	66 ± 7	52-79	1166	67 ± 6	55-79
	50-59	[7], [9], [19], [20], [21]	1668	66 ± 6	53-78	2250	68 ± 6	55-81
	60-69	[7], [19], [20], [21]	1419	67 ± 7	53-81	1812	69 ± 7	56-83
	≥70	[7], [20], [21]	1435	66 ± 7	52-79	1313	68 ± 6	56-81
LVM (g)	20-29	[7], [9], [19], [21]	193	130 ± 24	82-177	178	87 ± 16	54-119
	30-39	[7], [9], [19], [20], [21]	320	125 ± 22	81-169	289	85 ± 17	51-118
	40-49	[7], [9], [19], [20], [21]	897	125 ± 23	79-170	1139	85 ± 19	47-123
	50-59	[7], [9], [19], [20], [21]	1664	123 ± 24	76-171	2237	89 ± 18	54-124
	60-69	[7], [19], [20], [21]	1423	124 ± 25	75-172	1811	91 ± 20	52-129
	≥70	[7], [20], [21]	1441	121 ± 29	65-176	1319	89 ± 23	44-133
LVM/BSA (g/m^2^)	20-29	[7], [9], [17], [19], [21]	218	69 ± 11	47-91	203	54 ± 9	36-72
	30-39	[7], [9], [17], [19], [20], [21]	344	66 ± 11	45-86	314	52 ± 9	35-70
	40-49	[7], [9], [17], [19], [21]	897	64 ± 11	42-86	1164	52 ± 11	31-73
	50-59	[7], [9], [19], [20], [21]	1662	63 ± 11	43-84	2249	52 ± 10	32-72
	60-69	[7], [19], [20], [21]	1421	64 ± 11	42-86	1811	54 ± 11	33-75
	≥70	[7], [20], [21]	1440	62 ± 13	37-86	1309	51 ± 12	29-74
LVCO (L/min)	20-29	[19], [21]	173	6.2 ± 1.8	2.7-9.7	158	5.1 ± 1.3	2.6-7.6
	30-39	[19], [21]	260	5.9 ± 1.9	2.2-9.6	215	5.1 ± 1.3	2.5-7.8
	40-49	[19], [21]	594	5.7 ± 1.8	2.2-9.3	624	4.9 ± 1.3	2.4-7.5
	50-59	[19], [21]	1206	5.4 ± 1.6	2.3-8.6	1337	4.7 ± 1.2	2.4-7.1
	60-69	[19], [21]	1109	5.3 ± 1.5	2.4-8.2	1241	4.7 ± 1.0	2.6-6.7
	≥70	[21]	1377	5.5 ± 1.1	3.3-7.8	1219	4.7 ± 1.0	2.8-6.6
LVM/LVEDV (g/mL)	20-29	-^c^	-^c^	-^c^	-^c^	-^c^	-^c^	-^c^
	30-39	[20]	38	0.8 ± 0.1	0.6-1.1	46	0.7 ± 0.1	0.5-0.9
	40-49	[20]	285	0.8 ± 0.1	0.5-1.1	491	0.7 ± 0.1	0.5-0.9
	50-59	[20]	430	0.8 ± 0.2	0.5-1.1	888	0.7 ± 0.1	0.5-1.0
	60-69	[20]	306	0.9 ± 0.2	0.5-1.2	557	0.8 ± 0.1	0.5-1.0
	≥ 70	[20]	55	0.9 ± 0.2	0.5-1.2	85	0.8 ± 0.1	0.5-1.1

*Data are means ± standard deviation. BSA* body surface area, *bSSFP* balanced steady-state free precession, *n* number of study subjects, *SD* standard deviation, *LL* lower limit, *UL* upper limit, *LV* left ventricular, *EDV* end-diastolic volume, *ESV* end-systolic volume, *SV* stroke volume, *EF* ejection fraction, *M* mass, *CO* cardiac output, *CI* cardiac index

^a^Measured on a short-axis cine stack, ^b^in years, ^c^insufficient data

References [20], [21] provided additional data for analysis

**Table 6:** Absolute and indexed (BSA) left ventricular diameters in men and women for bSSFP imaging.

		Men			Women		
Diameter	References	n	Mean ± SD	LL-UL	n	Mean ± SD	LL-UL
LV end-diastolic 4ch (mm)	[9]	54	49 ± 5	39-59	53	46 ± 5	36-56
LV end-diastolic 4ch/BSA (mm/m^2^)	[9]	54	26 ± 4	18-34	53	27 ± 4	19-35
LV end-diastolic SAX (mm)	[10], [12], [16]	500	52 ± 5	44-61	672	48 ± 4	40-56
LV end-diastolic SAX/BSA (mm/m^2^)	[16]	100	26 ± 3	20-32	100	32 ± 4	24-40
LV end-systolic 4ch (mm)	[9]	54	32 ± 3	26-38	53	28 ± 6	16-40
LV end-systolic 4ch/BSA (mm/m^2^)	[9]	54	17 ± 2	13-21	53	17 ± 4	9-25
LV end-systolic SAX (mm)	[12]	60	34 ± 3	28-40	60	31 ± 4	23-39

*Data are means ± standard deviations. BSA* body surface area, *bSSFP* balanced steady-state free precession, *n* number of study subjects, *SD* standard deviation, *LL* lower limit, *UL* upper limit, *LV* left ventricular, *4ch* four-chamber view, *SAX* short-axis view

**Age:** LV volumes and mass change with age. In younger adults, the LV volume is larger and LVM is higher compared to older adults. With increasing age, LV volumes decrease, while LVM may remain relatively stable or increase due to myocardial remodeling [29]. Therefore, age-specific reference ranges may be useful in some situations.

**Ethnicity:** Caucasian adults generally have larger LV volumes compared to Black adults who tend to have higher LV mass [21]. Chinese and South Asian populations exhibit smaller LV dimensions and mass, with Chinese women having notably higher ejection fractions. These differences highlight the utility of ethnicity-specific reference ranges for CMR interpretation.

**Anthropometric measures:** Normal LV parameters vary by body size. LV parameters are indexed to BSA to reduce the effect of body size on normal values. Allometric indexing methods (e.g., height^2.7^) have been suggested as alternatives to BSA-based indexation [10], although the superiority of one indexation method over another has not been established.

**Acquisition technique:** bSSFP is established as the standard cine sequence for contemporary scanning. All studies include short-axis and most include at least two long-axis slices, with some including three. Most studies used 1.5T scanners. Two studies in Chinese populations used 3T scanners. The effect of 3T versus 1.5T CMR on normal LV values has not been determined.

**Post-processing:** Variability in post-processing software and segmentation techniques substantially affect LV parameters [30]. A major source of variation is handling of papillary muscles and trabeculae [31]. Inclusion of papillary muscles as part of LVM (and exclusion from LV volume) results in higher LVM, lower LV volumes, and higher LVEF. Further variations in published methods exist, including inclusion of papillary muscles (but not trabeculae) in LVM and measurement of LVM in systole compared to diastole [20]. There is need for standardization of segmentation practices to ensure global cross-comparability of derived metrics [32].

## 4 Normal volumes, mass, dimensions, and function of the right ventricle in adults

### 4.1 Influencing factors

**Gender:** RV volumes and mass are generally higher in men compared to women [34]. Notably, the measurement of RVM is subject to variability between studies due to lower reproducibility than LV parameters [20].

**Table 7:** References. Normal volumes, mass, dimensions, and function of the right ventricle in adults.

First author, year n, male:female Age range Study population	CMR acquisition	Parameter	Post-processing (Fig. 1)
Hudsmith, 2005 [6] 63:45 21-68 years Volunteers, UK	• 1.5T, cine bSSFP • Short-axis stack	• RVEF, RVM, RVEDV, RVESV, RVSV • Absolute, indexed to BSA	• Argus software (Version 2002B)^a^. Manual tracing of the endocardial and epicardial contour at end-diastole and end-systole. • Volumes below the pulmonary valve were included as part of the RV. • From the inflow tract, RV volumes were excluded if the surrounding muscle was thin and not trabeculated, suggestive of right atrium.
Maceira, 2006 [33] 60:60 20-80 years Volunteers, UK	• 1.5T, cine bSSFP • Short-axis stack, 2ch, 4ch	• RVEDV, RVESV, RVSV, RVEF, RVM • Absolute, indexed to BSA	• Semiautomatic software used for contouring (CMRtools)^b^ of endocardial and epicardial borders in all planes in all cardiac phases. • Systolic descent and twist of the tricuspid valve from tracking of the valve motion on the long-axis cines was used to correct for loss of systolic RV volume due to atrioventricular ring descent.
Chang, 2012 [8] 64:60 20-70 years Volunteers, Korea	• 1.5T, cine bSSFP • Short-axis stack	• RVEDV, RVESV, RVEF, RVSV, RVCO, RVCI • Absolute, indexed to BSA	• Manual contouring at end-systole and end-diastole using Argus software (version 4.02).^a^ • RV analysis was performed in selected end-diastolic and end-systolic frames based on the cavity size. • The RV outflow tract was included in the RV volume. • The RV base was determined by detection of the tricuspid annulus in the short-axis stack.
Macedo, 2013 [9] 54:53 20-80 years Substudy of CMR-LAC registry, Brazil	• 1.5T, cine bSSFP • Short-axis stack, 2ch, 4ch	• RVEDV, RVESV, RVDD, RVSD, RVEF, RVM • Absolute, indexed to BSA	• Semi-automatic contouring at end-systole and end-diastole using View Forum^c^ and Argus software.^a^ • The volumes below the pulmonary valve were included in the RV. • From the inflow tract, RV volumes were excluded if the surrounding muscle was thin and not trabeculated, suggestive of right atrium.
Le Ven, 2016 [11] 196:238 18-36 years Caucasian volunteers, Canada	• 1.5T, cine bSSFP • Short-axis stack, 2ch, 4ch, 3ch	• RVEDV, RVESV, RVSV, RVEF • Absolute, indexed to BSA	• Manual segmentation in end-diastole and end-systole using cmr^42^ software (version 3.4.1).^d^ • The endocardial border was traced from the most apical to the most basal slice. The RV outflow tract was accounted for in the RV volume, up to the level of the pulmonary valve. • Trabeculations and moderator band of the RV were ignored, and a smooth endocardial border was drawn. The moderator band was included in blood pool.
Lei, 2017 [12] 60:60 23-83 years Volunteers, China	• 3.0T, bSSFP • Short-axis stack, 2ch, 4ch, 3ch	• RVEDV, RVESV, RVSV, RVEF • Absolute, indexed to BSA	• Manual analysis in end-diastole and end-systole using Qmass software (version 7.6).^e^ • The volume below the level of the pulmonary valve was included in the RV. In the basal slices, the outflow tract was included in the RV volume.
Liu, 2018 [15] 50:50 20-70 years Volunteers, UK	• 1.5T, cine bSSFP • Short-axis stack, 2ch, 4ch	• RVEF, RVEDV, RVESV • Indexed to BSA	• Manual segmentation in end-diastole and end-systole using cvi42 software (version 5.3.4).^d^
Zhang, 2021 [19] 323:227 21-70 years Screening cohort, China	• 1.5T, cine bSSFP • Short-axis stack, 2ch, 4ch, 3ch	• RVEDV, RVESV, RVSV, RVEF, RVCO • Absolute, indexed to BSA	• Semi-automatic segmentation in end-diastole and end-systole using cvi42 software (version 5.6.2).^d^ • From the inflow tract, RV volumes were excluded if the surrounding muscle was thin and not trabeculated, suggestive of right atrium. • Trabeculae and papillary muscles were included in the RV blood pool.
Luu, 2022 [20] 1126:2080 35-75 years CAHHM, Canada	• 1.5T and 3.0T, cine bSSFP • Short-axis stack, 2ch, 4ch	• RVEF, RVESV, RVEDV, RVSV • Indexed to BSA	• Segmentation in end-diastole and end-systole using cvi42 software (version 4).^d^ • Epicardial and endocardial contours were traced manually or semi-automatically using the built-in threshold tool. • Trabeculae and papillary muscles were excluded from the RV blood pool.
Raisi-Estabragh, 2024 [21] 4452:4636 18-83 years Healthy Hearts Consortium^f^	• 1.5T and 3.0T, cine bSSFP • Short-axis stack, 2ch, 4ch	• RVEDV, RVSV, RVCO, RVEF • Absolute, indexed to BSA, and indexed to height	• Fully automated batch processing using the cvi42 software (prototype version 5.14).^d^ • Results presented with both trabeculae and papillary muscles 1) excluded and 2) included in the RV blood pool.

*n* number of study subjects, *T* Tesla, *bSSFP* balanced steady-state free precession, *RV* right ventricular, *EF* ejection fraction, *M* mass, *EDV* end-diastolic volume, *ESV* end-systolic volume, *SV* stroke volume, *BSA* body surface area, *2ch* two-chamber view, *4ch* four-chamber view, *CO* cardiac output, *CI* cardiac index, *CMR-LAC registry* The Latin-American Multi-Center reference study of CMR, *DD* diastolic diameter, *SD* systolic diameter, *CAHHM* Canadian Alliance for Healthy Heart and Minds

^a^Siemens Healthineers AG, Erlangen, Germany; ^b^Cardiovascular Imaging Solutions, London, UK; ^c^Koninklijke Philips N.V., Amsterdam, The Netherlands; ^d^Circle Cardiovascular Imaging Inc., Calgary, Alberta, Canada; ^e^Medis Medical Imaging Systems, Leiden, The Netherlands; ^f^UK Biobank, UK (n = 7672) [22] + SHIP, Germany (Study of Health in Pomerania) (n = 627) [23] + Italy (n = 291) [24] + Germany (n = 190) [25] + Singapore (n = 180) [26] + The Netherlands (n = 128) [27]

**Table 8:** Absolute and indexed (BSA) parameters of right ventricular volume, mass and function in men and women for bSSFP imaging; papillary muscles/ trabeculations included in right ventricular mass.^a^

		Men			Women		
Parameter	References	n	Mean ± SD	LL-UL	n	Mean ± SD	LL-UL
RVEDV (mL)	[6], [20], [21], [33]	5641	172 ± 33	107-237	6760	130 ± 25	81-179
RVEDV/BSA (mL/m^2^)	[6], [15], [20], [21], [33]	5686	83 ± 17	49-117	6806	73 ± 13	47-99
RVESV (mL)	[6], [20], [21], [33]	5639	72 ± 21	30-113	6749	50 ± 14	23-77
RVESV/BSA (mL/m^2^)	[6], [15], [20], [21], [33]	5694	34 ± 11	12-56	6797	27 ± 8	11-43
RVSV (mL)	[6], [20], [21], [33]	5640	101 ± 22	58-144	6755	80 ± 16	48-112
RVSV/BSA (mL/m^2^)	[6], [20], [21], [33]	5639	51 ± 11	30-72	6761	46 ± 9	29-64
RVEF (%)	[6], [15], [20], [21], [33]	5689	60 ± 9	44-77	6803	63 ± 7	49-77
RVM (g)	[6], [21], [33]	4433	50 ± 16	19-81	4600	39 ± 10	20-58
RVM/BSA (g/m^2^)	[6], [21], [33]	4440	25 ± 8	10-41	4596	22 ± 6	11-34
RVCO (L/min)	[21]	4397	6.1 ± 1.4	3.4-8.8	4567	5.0 ± 1.1	2.9-7.2

*Data are means ± standard deviations. BSA* body surface area, *bSSFP* balanced steady-state free precession, *n* number of study subjects, *SD* standard deviation, *LL* lower limit, *UL* upper limit, *RV* right ventricular, *EDV* end-diastolic volume, *ESV* end-systolic volume, *SV* stroke volume, *EF* ejection fraction, *M* mass, *CO* cardiac output

^a^Measured on a short-axis cine stack

References [20], [21] provided additional data for analysis

**Table 9:** Absolute and indexed (BSA) parameters of right ventricular volume, mass and function in men and women for bSSFP imaging; papillary muscles/trabeculations included in right ventricular volume.^a^

		Men			Women		
Parameter	References	n	Mean ± SD	LL-UL	n	Mean ± SD	LL-UL
RVEDV (mL)	[8], [9], [11], [12], [19], [21]	5097	152 ± 40	74-231	5219	115 ± 29	59-172
RVEDV/BSA (mL/m^2^)	[8], [9], [11], [12], [19], [21]	5095	82 ± 18	47-116	5221	71 ± 14	44-99
RVESV (mL)	[8], [9], [11], [12], [19], [21]	5103	65 ± 20	26-104	5216	46 ± 15	17-75
RVESV/BSA (mL/m^2^)	[8], [9], [11], [12], [19], [21]	5102	34 ± 9	16-52	5214	28 ± 8	13-43
RVSV (mL)	[8], [11], [12], [19], [21]	5041	89 ± 29	33-146	5166	71 ± 20	32-109
RVSV/BSA (mL/m^2^)	[8], [11], [19], [21]	4984	48 ± 14	21-76	5114	45 ± 10	25-64
RVEF (%)	[8], [9], [11], [12], [19], [21]	5095	58 ± 7	44-72	5219	61 ± 7	47-75
RVM (g)	[9], [21]	4366	36 ± 9	18-54	4550	29 ± 7	15-43
RVM/BSA (g/m^2^)	[9], [21]	4372	18 ± 4	11-26	4550	16 ± 4	9-24
RVCO (L/min)	[8], [19], [21]	4779	5.5 ± 1.6	2.4-8.6	4852	4.6 ± 1.2	2.2-7.1
RVCI (L/min/m^2^)	[8]	64	2.9 ± 0.9	1.1-4.7	60	2.7 ± 0.7	1.3-4.1

*Data are means ± standard deviations. BSA* body surface area, *bSSFP* balanced steady-state free precession, *n* number of study subjects, *SD* standard deviation, *LL* lower limit, *UL* upper limit, *RV* right ventricular, *EDV* end-diastolic volume, *ESV* end-systolic volume, *SV* stroke volume, *EF* ejection fraction, *M* mass, *CO* cardiac output, *CI* cardiac index

^a^Measured on a short-axis cine stack

References [8], [21] provided additional data for analysis.

**Table 10:** Absolute and indexed (BSA) parameters of right ventricular volume, mass and function in men and women by ethnicity for bSSFP imaging; papillary muscles/trabeculation included in right ventricular volume.^a^

			Men			Women		
Parameter	Ethnicity	References	n	Mean ± SD	LL-UL	n	Mean ± SD	LL-UL
RVEDV (mL)	Black	[21]	147	171 ± 30	112-230	189	137 ± 26	86-189
	Caucasian	[11], [21]	3815	184 ± 36	113-255	3950	135 ± 25	87-184
	Chinese	[12], [19], [21]	530	136 ± 27	83-190	475	102 ± 20	63-141
	Latin American	[9]	54	149 ± 34	81-217	53	115 ± 26	63-167
	South Asian	[21]	316	143 ± 26	92-194	188	110 ± 19	73-147
RVEDV/BSA (mL/m^2^)	Black	[21]	147	84 ± 13	58-109	189	74 ± 12	51-97
	Caucasian	[11], [21]	3815	93 ± 19	56-130	3952	79 ± 15	50-107
	Chinese	[12], [19], [21]	530	76 ± 13	50-102	475	66 ± 11	44-88
	Latin American	[9]	54	78 ± 16	46-110	53	69 ± 15	39-99
	South Asian	[21]	315	75 ± 12	52-99	188	65 ± 10	46-85
RVESV (mL)	Black	[21]	146	71 ± 16	39-103	189	53 ± 14	26-81
	Caucasian	[11], [21]	3823	73 ± 18	37-109	3947	51 ± 13	26-77
	Chinese	[12], [19], [21]	530	57 ± 16	26-89	475	38 ± 10	19-58
	Latin American	[9]	54	64 ± 19	26-102	53	45 ± 14	17-73
	South Asian	[21]	317	58 ± 13	32-85	188	41 ± 9	22-59
RVESV/BSA (mL/m^2^)	Black	[21]	146	35 ± 8	20-50	188	29 ± 7	16-42
	Caucasian	[11], [21]	3821	37 ± 9	19-55	3946	30 ± 7	15-44
	Chinese	[12], [19], [21]	530	32 ± 8	17-48	475	25 ± 6	13-36
	Latin American	[9]	54	33 ± 9	15-51	53	27 ± 9	9-45
	South Asian	[21]	317	31 ± 7	18-44	187	24 ± 5	14-34
RVSV (mL)	Black	[21]	145	99 ± 19	63-136	189	84 ± 17	52-117
	Caucasian	[11], [21]	3815	111 ± 26	60-162	3952	84 ± 17	51-116
	Chinese	[12], [19], [21]	530	79 ± 15	49-108	475	64 ± 13	39-90
	Latin American	-^b^	-^b^	-^b^	-^b^	-^b^	-^b^	-^b^
	South Asian	[21]	317	85 ± 17	52-117	188	69 ± 12	45-94
RVSV/BSA (mL/m^2^)	Black	[21]	147	49 ± 9	32-67	189	45 ± 8	30-60
	Caucasian	[11], [21]	3817	56 ± 14	29-82	3958	49 ± 10	30-69
	Chinese	[19], [21]	470	44 ± 8	28-60	415	43 ± 7	29-56
	Latin American	-^b^	-^b^	-^b^	-^b^	-^b^	-^b^	-^b^
	South Asian	[21]	316	44 ± 8	29-60	188	41 ± 7	28-54
RVEF (%)	Black	[21]	147	59 ± 6	47-70	189	62 ± 6	50-73
	Caucasian	[11], [21]	3813	60 ± 7	46-74	3950	62 ± 6	50-75
	Chinese	[12], [19], [21]	530	58 ± 6	47-70	475	63 ± 5	53-73
	Latin American	[9]	54	57 ± 7	43-71	53	61 ± 8	45-77
	South Asian	[21]	317	59 ± 6	48-70	189	63 ± 5	53-73
RVM (g)	Black	[21]	148	41 ± 6	30-52	187	35 ± 5	25-45
	Caucasian	[21]	3619	41 ± 7	28-53	3718	33 ± 5	22-43
	Chinese	[21]	56	37 ± 4	29-45	99	31 ± 4	23-38
	Latin American	[9]	54	29 ± 8	13-45	53	24 ± 8	8-40
	South Asian	[21]	317	37 ± 5	26-47	188	30 ± 4	22-38
RVM/BSA (g/m^2^)	Black	[21]	147	20 ± 2	16-25	189	19 ± 2	14-24
	Caucasian	[21]	3628	20 ± 3	14-26	3718	18 ± 3	13-24
	Chinese	[21]	56	21 ± 2	16-25	99	19 ± 3	14-24
	Latin American	[9]	54	16 ± 4	8-24	53	15 ± 5	5-25
	South Asian	[21]	315	19 ± 2	15-24	188	18 ± 2	14-22
RVCO (L/min)	Black	[21]	148	6.3 ± 1.4	3.6-9.0	188	5.4 ± 1.2	3.0-7.8
	Caucasian	[21]	3610	6.4 ± 1.5	3.5-9.3	3700	5.3 ± 1.2	3.0-7.6
	Chinese	[19], [21]	469	5.2 ± 1.1	3.1-7.4	415	4.4 ± 0.9	2.6-6.1
	Latin American	-^b^	-^b^	-^b^	-^b^	-^b^	-^b^	-^b^
	South Asian	[21]	318	5.3 ± 1.1	3.1-7.5	187	4.5 ± 0.9	2.7-6.3

*Data are means ± standard deviation. BSA* body surface area, *bSSFP* balanced steady-state free precession, *n* number of study subjects, *SD* standard deviation, *LL* lower limit, *UL* upper limit, *RV* right ventricular, *EDV* end-diastolic volume, *ESV* end-systolic volume, *SV* stroke volume, *EF* ejection fraction, *M* mass, *CO* cardiac output

^a^Measured on a short-axis cine stack; ^b^insufficient data

Reference [21] provided additional data for analysis

**Table 11:** Absolute and indexed (BSA) parameters of right ventricular volume, mass and function in men and women of mixed ethnicity by age for bSSFP imaging; papillary muscles/trabeculation included in left ventricular volume.^a^

			Men			Women		
Parameter	Age^b^	References	n	Mean ± SD	LL-UL	n	Mean ± SD	LL-UL
RVEDV (mL)	20-29	[8], [9], [19], [21]	196	174 ± 41	93-254	181	124 ± 27	72-176
	30-39	[8], [9], [19], [21]	287	156 ± 38	81-230	243	117 ± 25	68-166
	40-49	[8], [9], [19], [21]	613	150 ± 41	70-231	647	119 ± 29	63-175
	50-59	[8], [9], [19], [21]	1235	146 ± 40	67-225	1359	111 ± 29	54-167
	60-69	[8], [19], [21]	1122	132 ± 42	50-214	1258	109 ± 28	54-164
	≥70	[21]	1380	157 ± 29	101-214	1228	119 ± 22	77-162
RVEDV/BSA (mL/m^2^)	20-29	[8], [9], [19], [21]	196	93 ± 19	56-129	181	77 ± 13	51-102
	30-39	[8], [9], [19], [21]	286	80 ± 16	50-111	243	73 ± 13	47-100
	40-49	[8], [9], [19], [21]	611	79 ± 18	45-114	645	71 ± 13	45-97
	50-59	[8], [9], [19], [21]	1236	77 ± 18	42-112	1370	66 ± 13	41-91
	60-69	[8], [19], [21]	1122	72 ± 17	38-105	1254	66 ± 12	41-90
	≥70	[21]	1380	79 ± 14	52-106	1225	69 ± 11	47-90
RVESV (mL)	20-29	[8], [9], [19], [21]	196	83 ± 32	20-147	181	57 ± 20	17-97
	30-39	[8], [9], [19], [21]	287	71 ± 21	29-113	243	49 ± 19	13-86
	40-49	[8], [9], [19], [21]	615	66 ± 23	21-111	649	48 ± 16	17-78
	50-59	[8], [9], [19], [21]	1235	63 ± 22	19-107	1361	43 ± 15	14-73
	60-69	[8], [19], [21]	1125	54 ± 20	14-93	1253	40 ± 14	12-68
	≥70	[21]	1381	64 ± 15	34-94	1226	43 ± 11	22-65
RVESV/BSA (mL/m^2^)	20-29	[8], [9], [19], [21]	196	43 ± 16	12-73	181	33 ± 11	11-55
	30-39	[8], [9], [19], [21]	286	36 ± 9	18-55	243	31 ± 11	9-53
	40-49	[8], [9], [19], [21]	611	35 ± 11	14-55	647	28 ± 8	12-45
	50-59	[8], [9], [19], [21]	1240	33 ± 11	12-55	1366	26 ± 8	11-41
	60-69	[8], [19], [21]	1124	29 ± 9	12-46	1250	24 ± 7	10-38
	≥70	[21]	1381	32 ± 7	18-47	1224	25 ± 6	14-36
RVSV (mL)	20-29	[8], [19], [21]	186	93 ± 34	27-160	171	70 ± 19	33-106
	30-39	[8], [19], [21]	275	85 ± 25	36-133	225	69 ± 23	24-114
	40-49	[8], [19], [21]	602	84 ± 29	28-141	638	74 ± 19	36-111
	50-59	[8], [19], [21]	1223	85 ± 26	35-135	1354	69 ± 20	30-108
	60-69	[8], [19], [21]	1122	79 ± 26	27-130	1255	69 ± 17	35-103
	≥70	[21]	1377	93 ± 19	56-130	1225	76 ± 15	47-104
RVSV/BSA (mL/m^2^)	20-29	[8], [19], [21]	186	49 ± 16	18-80	171	43 ± 9	25-62
	30-39	[8], [19], [21]	274	44 ± 11	23-65	225	43 ± 11	22-64
	40-49	[8], [19], [21]	605	44 ± 13	20-69	636	44 ± 9	27-61
	50-59	[8], [19], [21]	1223	44 ± 11	23-66	1361	41 ± 9	25-58
	60-69	[8], [19], [21]	1126	42 ± 11	20-64	1256	42 ± 8	27-58
	≥70	[21]	1374	47 ± 9	29-65	1227	44 ± 8	28-59
RVEF (%)	20-29	[8], [9], [19], [21]	196	53 ± 13	27-78	181	55 ± 11	35-76
	30-39	[8], [9], [19], [21]	287	55 ± 7	41-69	243	58 ± 11	35-80
	40-49	[8], [9], [19], [21]	615	57 ± 8	41-72	650	61 ± 7	47-75
	50-59	[8], [9], [19], [21]	1240	57 ± 7	44-71	1368	62 ± 7	49-75
	60-69	[8], [19], [21]	1123	60 ± 6	48-72	1255	64 ± 7	51-77
	≥70	[21]	1370	59 ± 6	48-71	1219	64 ± 6	53-75
RVM (g)	20-29	[9], [21]	123	35 ± 7	21-49	112	26 ± 7	12-39
	30-39	[9], [21]	147	34 ± 6	22-47	155	27 ± 4	19-35
	40-49	[9], [21]	490	35 ± 11	14-57	560	28 ± 10	8-48
	50-59	[9], [21]	1156	35 ± 12	11-58	1290	34 ± 6	23-45
	60-69	[21]	1065	41 ± 6	28-53	1203	33 ± 5	23-43
	≥70	[21]	1377	40 ± 6	28-51	1225	31 ± 5	22-40
RVM/BSA (g/m^2^)	20-29	[9], [21]	123	18 ± 4	11-26	112	15 ± 4	7-23
	30-39	[9], [21]	146	17 ± 3	11-23	155	16 ± 2	11-20
	40-49	[9], [21]	489	19 ± 4	11-27	557	16 ± 5	6-26
	50-59	[9], [21]	1159	18 ± 5	9-27	1294	16 ± 5	5-26
	60-69	[21]	1069	20 ± 3	14-26	1202	18 ± 3	13-24
	≥70	[21]	1378	20 ± 3	15-25	1225	18 ± 2	13-23
RVCO (L/min)	20-29	[8], [19], [21]	186	6.4 ± 2.4	1.7-11.0	171	4.7 ± 1.6	1.5-7.9
	30-39	[8], [19], [21]	275	5.9 ± 1.9	2.1-9.7	225	4.8 ± 1.6	1.5-8.0
	40-49	[8], [19], [21]	601	5.6 ± 2.0	1.8-9.5	635	4.9 ± 1.4	2.3-7.6
	50-59	[8], [19], [21]	1217	5.6 ± 1.6	2.4-8.8	1350	4.5 ± 1.3	1.9-7.1
	60-69	[8], [19], [21]	1123	5.2 ± 1.6	2.1-8.3	1251	4.6 ± 1.2	2.3-6.9
	≥70	[21]	1377	5.8 ± 1.3	3.4-8.3	1220	4.9 ± 1.1	2.8-7.0

*Data are means ± standard deviations. BSA* body surface area, *bSSFP* balanced steady-state free precession, *n* number of study subjects, *SD* standard deviation, *LL* lower limit, *UL* upper limit, *RV* right ventricular, *EDV* end-diastolic volume, *ESV* end-systolic volume, *SV* stroke volume, *EF* ejection fraction, *M* mass, *CO* cardiac output, *CI* cardiac index

^a^Measured on a short-axis cine stack, ^b^in years, ^c^insufficient data

References [8], [21] provided additional data for analysis

**Age:** RV volumes are lower in adults of older age for both men and women [35]. Thus, age-specific reference ranges may be useful in some clinical settings.

**Ethnicity:** RV parameters vary by ethnicity. For example, Caucasians have the largest RVEDV compared to other ethnic groups, while Chinese individuals have the smallest [19].

**Anthropometric measures:** RV parameters are indexed to BSA in order to account for variation by body size. Other indexing methods (e.g., height) have been proposed but are infrequently used.

**Acquisition technique:** Differences in scanner field strength, imaging protocols, and post-processing techniques can affect measurements [3].

**Post-processing:** Differences in post-processing software and techniques affect RV parameter measurements. In general, fully-automated software methods for RV analysis are less reliable for RV compared to LV segmentation. Thus, software-derived RV contours frequently require manual correction and are more prone to inter-observer variations. Post-processing by the same operator with the same software aids comparison of serial RV parameters for an individual patient. AI-aided software have shown potential for improving the reproducibility of RV analysis [36].

## 5 Normal values of left atrial dimensions and functions in adults

### 5.1 Influencing factors

**Gender:** Absolute volumes of the left atrium are generally reported to be larger for men compared to women [6]. After indexing for body surface area (BSA), significant differences in left atrial size between men and women are reduced, or in some reports, reversed. In a study of 120 healthy participants (60 males, 60 females), males had significantly larger absolute LA volumes, diameters, and areas, except for transverse and anteroposterior diameters, which showed no differences. However, when adjusted for BSA, females had larger longitudinal, transverse, and anteroposterior diameters [38]. Le Ven et al. described larger maximal LA volumes indexed to BSA in 195 healthy men compared to 239 healthy women (18-36 years) [11]. In 408 healthy Chinese (21-70 years), Gao et al. found larger absolute maximal LA volumes in men compared to women, but after indexation to BSA women had larger volumes compared to men [42].

**Fig. 3:**
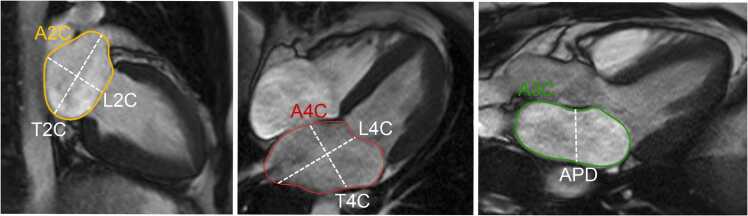
Measurements of left atrial area (A2C, A4C, A3C), longitudinal (L2C, L4C), and anteroposterior (APD) diameters on the two-, four-, and three-chamber view according to reference [38]

**Fig. 4:**
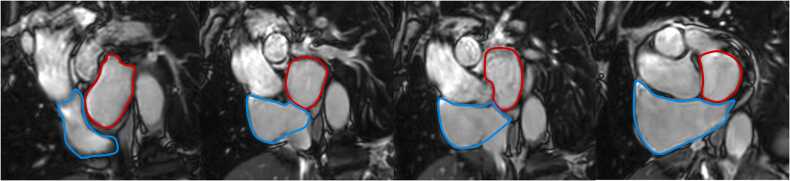
Measurements of maximal left and right atrial volume using the Simpson’s method, obtained on a short-axis cine stack of balanced steady-state free precession images covering the atria, by contouring left (red) and right (blue) atrial borders at ventricular systole according to reference [11]

**Table 12:** References. Normal values of left atrial dimensions and functions in adults.

First author, year n, male:female Age range Study population	CMR acquisition	Parameter	Post-processing (Figs. 3 and 4)
Sievers, 2005 [37] 59:52 25-73 years Volunteers, Germany	• 1.5T, bSSFP • 2ch, 4ch	• Maximal LA transverse diameter (absolute and indexed to BSA)	• On the 2ch- and 4ch-view, respectively, the LA transverse diameter was measured in the middle of the LA at ventricular end-systole.
Maceira, 2010 [38] 60:60 20-80 years Volunteers, UK	• 1.5T, bSSFP • 2ch, 3ch, 4ch • SAX cine stack covering the entire LA	• Maximal LA volume (absolute and indexed to BSA) • Maximal LA area (absolute and indexed to BSA) on a 2ch-, 3ch- and 4ch-view • Maximal LA longitudinal and transverse diameter (absolute and indexed to BSA) of a 2ch- and 4ch-view • Maximal LA antero-posterior diameter (absolute and indexed to BSA) of a 3ch-view	• Measurements were obtained at ventricular systole. • LA volume was calculated by a semi-automated software (CMRTools^a^) after delineation of the atrial endocardial borders in all planes (including the LA appendage, excluding the pulmonary veins). • LA areas were planimetered in the 2ch-, 3ch-, and 4ch-view (excluding the LA appendage and the pulmonary veins). • Longitudinal and transverse LA diameters were measured in the 2ch- and 4ch-view and LA antero-posterior diameter on the 3ch-view as shown in Fig. 3.
Le Ven, 2016 [11] 196:238 18-36 years Caucasian volunteers, Canada	• 1.5T, bSSFP • 2ch, 3ch, 4ch • SAX cine stack covering the entire LA	• Maximal LA volume [LA end-diastolic volume] (absolute and indexed to BSA) • Minimal LA volume [LA end-systolic volume] (absolute and indexed to BSA) • LA stroke volume (absolute and indexed to BSA) • LA ejection fraction	• Long-axis planes were used to place a boundary at the level of the atrio-ventricular valve annulus. • LA endocardial borders were manually traced (using cvi42, version 3.4.1^b^) in atrial end-diastolic and end-systolic phases on contiguous short-axis slices, end-diastole referring to the atrial diastole (maximum volume). • The atrial appendage was included in the total LA volume, but the pulmonary veins were excluded. • Volumes were calculated from Simpson’s rule.
Li, 2017 [39] 66:69 23-83 years Volunteers, China	• 3.0T, bSSFP • 2ch, 3ch, 4ch • SAX cine stack covering the entire LA	• Maximal LA longitudinal and transverse diameter (absolute and indexed to BSA) of a 2ch- and 4ch-view • Maximal LA antero-posterior diameter (absolute and indexed to BSA) of a 3ch-view • Maximal LA volume (in SAX and biplane, absolute and indexed to BSA) • LA volume prior to atrial contraction (in SAX and biplane, absolute and indexed to BSA) • Minimal LA volume (in SAX and biplane, absolute and indexed to BSA)	• LA dimensions measured at the end of the systolic phase of the left ventricle (before the opening of the mitral valve) on 2-ch, 3-ch, and 4-ch bSSFP cine images in both bi-plane method and SAX method using Simpson’s method (using Qmass, version 7.6^c^). • The LA appendage and the pulmonary veins were excluded using the bi-plane area-length method.
Zemrak, 2017 [40] 109:174 65 ± 9^c^ years MESA, USA	• 1.5T, bSSFP • 2ch, 4ch • SAX cine stack covering the entire LA	• LA volume indexed to BSA, weight, height, and fat-free mass	• Horizontal and vertical long-axis cine bSSFP images were used for measuring the biplanar LA volume in LV end-systole, just before mitral valve opening. • Pulmonary veins and LA appendage were excluded from LA volume. • LA area and LA height from both vertical and horizontal planes were used to calculate the biplanar LA volume using the formula: LA volume = 8 × vertical area × horizontal area/(3 × PI × [(vertical length + horizontal length)/2]). • LA volume was also calculated based on a SAX cine stack using the Simpson’s method (using cvi42^b^).
Funk, 2018 [41] 105:77 19-76 years Volunteers, Germany	• 1.5T and 3.0T, bSSFP • 2ch, 4ch	• LA end-diastolic volume (absolute and indexed to height and BSA) • LA end-systolic volume (absolute and indexed to height and BSA) • LA stroke volume (absolute) • LA ejection fraction	• Bi-planar approach using 2ch and 4ch views (using cvi42 version 5.1^b^). • Pulmonary veins and LA appendage were excluded from LA volume.
Zhuang, 2020 [16] 100:100 20-70 years Volunteers, China	• 3.0T • bSSFP and turbo spin echo black blood imaging • 2ch, 3ch, and 4ch	• Antero-posterior and left-to-right LA diameter • Maximal LA longitudinal and transverse diameter (absolute and indexed to BSA) of a 2ch- and 4ch-view in diastole and systole • Maximal LA antero-posterior and lateral diameter (absolute and indexed to BSA) of a 3ch-view • Maximal LA volume in systole and diastole (absolute and indexed to BSA) • Maximal LA area in systole and diastole of a 2ch- and 4ch-view (absolute and indexed to BSA)	• Antero-posterior and left-to-right measured using an axial black blood sequence. • Antero-posterior diameters also measured on 3ch bSSFP. • bSSFP 2ch and 4ch views to measure transverse and longitudinal diameters, and LA area in end-diastole and end-systole.
Gao, 2022 [42] 220:188 21-70 years Healthy participants of CMR screening, China	• 1.5T, bSSFP • SAX cine stack covering the entire LA • 2ch, 3ch, 4ch	• Maximal, pre-atrial contraction, and minimal volume at end-diastole, end-systole, before atrial contraction, and at late ventricular diastole, respectively; all indexed to BSA	• LA endocardial contours manually traced in 2ch and 4ch views (using cvi42 version 5.12.1^b^); excluding pulmonary veins and the LA appendage using the bi-plane method.
Luu, 2022 [20] 1126:2080 35-75 years CAHHM, Canada	• 1.5T and 3.0T, bSSFP • 2ch and 4ch	• Maximal and minimal LA volumes in 2ch and 4ch views	• Maximal LA volume was measured at end-systole, immediately before mitral valve opening and minimum LA volume was measured at end-diastole, immediately before mitral valve closure (using cvi42 version 4^b^). • Using the bi-plane area-length method to calculate LA volume.
Raisi-Estabragh, 2024 [21] 4452:4636 18-83 years Healthy Hearts Consortium^d^	• 1.5T or 3.0T, bSSFP • 2ch and 4ch	• Maximal LA end-systolic volume (indexed to BSA and height) • LA ejection fraction	• LA volumes extracted from volume curves (using a fully automatic prototype of cvi42 version 5.14^b^), which were created using the bi-plane area-length method from 4ch- and 2ch-views: volume 1⁄4 (0.848 × area 4-chamber × area 2-chamber)/([length 2-chamber + length 4-chamber]/2).

*n* number of study subjects, *T* Tesla, *bSSFP* balanced steady-state free-precession, *2ch* two-chamber view, 3ch three-chamber view, 4ch four-chamber view, *LA* left atrial, *BSA* body surface area, *SAX* short-axis, *MESA* Multi-Ethnic Study in Atherosclerosis, *CAHHM* Canadian Alliance for Healthy Heart and Minds

^a^Cardiovascular Imaging Solutions, London, UK; ^b^Circle Cardiovascular Imaging Inc., Calgary, Alberta, Canada; ^c^Medis Medical Imaging Systems, Leiden, The Netherlands; ^c^mean ± SD (age-range not provided in original publication); ^d^UK Biobank, UK (n = 7672) [22] + SHIP, Germany (Study of Health in Pomerania) (n = 627) [23] + Italy (n = 291) [24] + Germany (n = 190) [25] + Singapore (n = 180) [26] + The Netherlands (n = 128) [27]

**Table 13:** Absolute and indexed (BSA) left atrial volumes and function in men and women for bSSFP imaging.

			Men			Women		
Method	Parameter	References	n	Mean ± SD	LL-UL	n	Mean ± SD	LL-UL
Bi-plane area-length method; LA appendage excluded	Max. LA volume (mL)	[16], [20], [21], [39], [40], [41], [42]	13,969	68 ± 22	26-110	15,563	60 ± 17	26-94
	Max. LA volume/BSA (mL/m^2^)	[16], [20], [21], [39], [40], [41], [42]	13,972	36 ± 10	16-56	15,557	36 ± 9	18-54
	Min. LA volume (mL)	[16], [20], [21], [39], [41], [42]	13,800	28 ± 14	2-55	15,359	24 ± 11	3-45
	Min. LA volume/BSA (mL/m^2^)	[16], [20], [21], [39], [41], [42]	13,803	15 ± 7	2-28	15,335	14 ± 6	3-26
	LASV (mL)	[20], [41]	1219	40 ± 14	13-68	2144	38 ± 15	10-67
	LASV/BSA (mL/m^2^)	[20]	1114	18 ± 6	6-29	2067	18 ± 6	7-30
	LA ejection fraction (%)	[20], [21], [39], [41], [42]	13,703	59 ± 13	35-84	15,175	61 ± 11	39-83
Simpson’s method; LA appendage included	Max. LA volume (mL)	[11], [38], [39]	322	75 ± 18	40-111	367	65 ± 14	38-93
	Max. LA volume/BSA (mL/m^2^)	[11], [38], [39]	322	40 ± 8	25-56	367	41 ± 8	25-56
	Min. LA volume (mL)	[11], [39]	262	32 ± 9	15-50	307	26 ± 8	11-41
	Min. LA volume/BSA (mL/m^2^)	[11], [39]	262	18 ± 4	9-26	307	17 ± 5	7-27
	LASV (mL)	[11]	196	47 ± 13	21-73	238	39 ± 10	19-59
	LASV/BSA (mL/m^2^)	[11]	196	24 ± 6	12-36	238	24 ± 5	14-34
	LA ejection fraction (%)	[11], [39]	262	57 ± 9	40-74	307	59 ± 7	45-73

*Data are means ± standard deviation. BSA* body surface area, *bSSFP* balanced steady-state free precession, *n* number of subjects, *SD* standard deviation, *LL* lower limit, *UL* upper limit, *LA* left atrial, *max.* maximal, *min.* minimal, *SV* stroke volume

**Table 14:** Absolute and indexed (BSA) left atrial volumes and function in men and women by ethnicity for bSSFP imaging using the bi-plane area-length method.

			Men			Women		
Parameter	Ethnicity	References	n	Mean ± SD	LL-UL	n	Mean ± SD	LL-UL
Max. LA volume (mL)	Black	[21]	429	71 ± 21	30-111	549	67 ± 17	35-100
	Caucasian	[20], [21]	10,869	74 ± 23	30-119	11,939	64 ± 18	29-99
	Chinese	[16], [20], [21], [39], [42]	957	65 ± 15	35-95	1252	57 ± 14	30-83
	South Asian	[20], [21]	996	63 ± 17	29-97	621	57 ± 13	31-83
Max. LA volume/BSA (mL/m^2^)	Black	[21]	435	35 ± 10	15-55	549	36 ± 8	20-52
	Caucasian	[20], [21]	10,869	37 ± 11	16-59	11,936	36 ± 9	18-55
	Chinese	[16], [20], [21], [39], [42]	957	36 ± 8	20-51	1252	36 ± 9	20-53
	South Asian	[20], [21]	993	32 ± 8	16-48	618	34 ± 7	20-48
Min. LA volume (mL)	Black	[21]	435	26 ± 12	3-49	549	24 ± 9	7-42
	Caucasian	[20], [21]	10,797	34 ± 18	-^a^	11,909	28 ± 13	3-52
	Chinese	[16], [20], [21], [39], [42]	957	28 ± 10	8-49	1252	24 ± 9	7-41
	South Asian	[20], [21]	996	28 ± 13	3-53	618	25 ± 11	4-46
Min. LA volume/BSA (mL/m^2^)	Black	[21]	435	13 ± 5	2-23	549	13 ± 5	4-22
	Caucasian	[20], [21]	10,800	14 ± 9	-^a^	11,885	16 ± 7	1-30
	Chinese	[16], [20], [21], [39], [42]	957	16 ± 5	5-26	1252	15 ± 5	4-26
	South Asian	[20], [21]	996	14 ± 6	2-26	618	15 ± 6	2-27
LASV (mL)	Black	-^b^	-^b^	-^b^	-^b^	-^b^	-^b^	-^b^
	Caucasian	[20]	861	36 ± 12	14-59	1604	32 ± 10	12-51
	Chinese	[20]	193	30 ± 10	11-50	352	26 ± 8	11-41
	South Asian	[20]	51	28 ± 11	6-50	63	26 ± 10	6-46
LASV/BSA (mL/m^2^)	Black	-^b^	-^b^	-^b^	-^b^	-^b^	-^b^	-^b^
	Caucasian	[20]	861	18 ± 6	7-30	1604	19 ± 6	7-30
	Chinese	[20]	193	16 ± 5	7-26	352	16 ± 5	6-26
	South Asian	[20]	51	14 ± 5	3-24	63	15 ± 6	4-27
LA ejection fraction (%)	Black	[21]	423	64 ± 7	50-78	549	64 ± 7	51-77
	Caucasian	[20], [21]	10,830	56 ± 17	23-90	11,846	58 ± 15	29-86
	Chinese	[20], [21], [39], [42]	857	57 ± 12	34-79	1149	59 ± 11	36-81
	South Asian	[20], [21]	987	56 ± 16	24-88	615	56 ± 16	25-87

*Data are means ± standard deviation. BSA* body surface area, *bSSFP* balanced steady-state free-precession, *n* number of subjects, *SD* standard deviation, *LL* lower limit, *UL* upper limit, *LA* left atrial, *max.* maximal, *min* minimal, *SV* stroke volume

^a^Limited reliability of the reference interval, ^b^insufficient data (small sample size)

**Table 15:** Absolute and indexed (BSA) left atrial volumes and function in men and women by age for bSSFP imaging using the bi-plane area-length method.

			Men			Women		
Parameter	Age^a^	References	n	Mean ± SD	LL-UL	n	Mean ± SD	LL-UL
Max. LA volume (mL)	20-29	[16], [21], [42]	256	72 ± 16	40-103	237	59 ± 15	30-89
	30-39	[16], [20], [21], [42]	366	70 ± 19	33-107	419	59 ± 13	34-85
	40-49	[16], [20], [21], [42]	1677	71 ± 19	34-108	2093	62 ± 17	29-95
	50-59	[16], [20], [21], [42]	3803	70 ± 21	28-112	4671	59 ± 18	24-94
	60-69	[16], [20], [21], [42]	3482	67 ± 23	23-112	4156	59 ± 19	21-96
	≥70	[20], [21]	4105	69 ± 23	24-115	3667	63 ± 20	24-102
Max. LA volume/BSA (mL/m^2^)	20-29	[16], [21], [42]	256	38 ± 8	22-54	237	37 ± 8	21-53
	30-39	[16], [20], [21], [42]	366	36 ± 9	19-54	419	36 ± 7	22-50
	40-49	[16], [20], [21], [42]	1677	37 ± 9	20-54	2093	37 ± 9	20-53
	50-59	[16], [20], [21], [42]	3809	36 ± 10	17-55	4680	35 ± 9	17-52
	60-69	[16], [20], [21], [42]	3491	36 ± 11	15-57	4150	35 ± 10	15-55
	≥70	[20], [21]	4093	36 ± 12	13-59	3658	37 ± 11	15-58
Min. LA volume (mL)	20-29	[16], [21], [42]	256	27 ± 7	13-42	237	20 ± 7	7-33
	30-39	[16], [20], [21], [42]	366	29 ± 10	9-49	419	23 ± 8	9-38
	40-49	[16], [20], [21], [42]	1677	29 ± 13	4-54	2093	25 ± 9	7-44
	50-59	[16], [20], [21], [42]	3812	32 ± 14	5-59	4680	25 ± 10	6-45
	60-69	[16], [20], [21], [42]	3485	30 ± 15	1-59	4144	26 ± 12	3-49
	≥70	[20], [21]	4033	34 ± 19	-^b^	3640	29 ± 15	-^b^
Min. LA volume/BSA (mL/m^2^)	20-29	[16], [21], [42]	256	15 ± 4	7-22	237	13 ± 4	5-20
	30-39	[16], [20], [21], [42]	366	15 ± 5	6-25	419	14 ± 4	6-22
	40-49	[16], [20], [21], [42]	1677	15 ± 6	3-27	2090	15 ± 5	5-25
	50-59	[16], [20], [21], [42]	3812	16 ± 7	4-29	4677	15 ± 6	4-26
	60-69	[16], [20], [21], [42]	3488	16 ± 7	2-30	4153	16 ± 7	3-29
	≥70	[20], [21]	4033	17 ± 10	-^b^	3613	17 ± 9	-^b^
LASV (mL)	20-29	-^c^	-^c^	-^c^	-^c^	-^c^	-^c^	-^c^
	30-39	[20]	38	34 ± 12	10-57	46	28 ± 8	13-42
	40-49	[20]	285	36 ± 12	12-59	491	32 ± 10	13-51
	50-59	[20]	430	35 ± 11	13-58	888	31 ± 10	12-50
	60-69	[20]	306	34 ± 11	13-56	557	29 ± 10	11-48
	≥70	[20]	55	32 ± 11	10-53	85	31 ± 11	9-53
LASV/BSA (mL/m^2^)	20-29	-^c^	-^c^	-^c^	-^c^	-^c^	-^c^	-^c^
	30-39	[20]	38	17 ± 6	5-28	46	17 ± 4	9-24
	40-49	[20]	285	18 ± 6	6-29	491	18 ± 6	7-30
	50-59	[20]	430	18 ± 6	7-30	888	18 ± 6	7-30
	60-69	[20]	306	18 ± 6	6-29	557	18 ± 6	6-29
	≥70	[20]	55	17 ± 6	5-28	85	18 ± 6	6-30
LA ejection fraction (%)	20-29	[21], [42]	236	62 ± 5	52-71	217	66 ± 6	54-78
	30-39	[20], [21], [42]	346	57 ± 11	35-79	399	59 ± 11	38-81
	40-49	[20], [21], [42]	1654	58 ± 13	33-83	2064	60 ± 11	39-82
	50-59	[20], [21], [42]	3783	57 ± 13	31-83	4636	59 ± 12	36-82
	60-69	[20], [21], [42]	3459	57 ± 15	28-86	4097	57 ± 13	31-84
	≥70	[20], [21]	4054	55 ± 19	17-92	3616	56 ± 16	24-87

*Data are means ± standard deviation. BSA* body surface area, *bSSFP* balanced steady-state free-precession, *n* number of subjects, *SD* standard deviation, *LL* lower limit, *UL* upper limit, *LA* left atrial, *max.* maximal, *min.* minimal, *SV* stroke volume

^a^In years, ^b^limited reliability of the reference interval, ^c^insufficient data

**Table 16:** Absolute and indexed (BSA) left atrial area and diameter in men and women for bSSFP imaging.

		Men				Women	
Parameter	References	n	Mean ± SD	LL-UL	n	Mean ± SD	LL-UL
Max. LA area 2ch (cm^2^)^a^	[16], [38]	160	21 ± 4	12-29	160	18 ± 4	9-27
Max. LA area 2ch/BSA (cm^2^/m^2^)^a^	[16], [38]	160	11 ± 2	7-16	160	11 ± 3	6-16
Max. LA area 3ch (cm^2^)^a^	[38]	60	19 ± 4	11-27	60	17 ± 4	9-25
Max. LA area 3ch/BSA (cm^2^/m^2^)^a^	[38]	60	10 ± 2	6-14	60	10 ± 2	6-14
Max. LA area 4ch (cm^2^)^a^	[16], [38]	160	21 ± 4	13-29	160	19 ± 4	11-27
Max. LA area 4ch/BSA (cm^2^/m^2^)^a^	[16], [38]	160	11 ± 2	7-15	160	11 ± 2	7-16
Max. LA longitudinal diameter 2ch (cm)	[16], [37], [38], [39]	285	4.9 ± 0.7	3.6-6.2	281	4.6 ± 0.7	3.2-6.0
Max. LA longitudinal diameter 2ch/BSA (cm/m^2^)	[16], [37], [38], [39]	285	2.6 ± 0.5	1.7-3.6	281	2.8 ± 0.6	1.6-4.0
Max. LA transverse diameter 2ch (cm)	[16], [38], [39]	226	4.4 ± 0.6	3.3-5.6	229	4.3 ± 0.5	3.3-5.3
Max. LA transverse diameter 2ch/BSA (cm/m^2^)	[16], [38], [39]	226	2.4 ± 0.3	1.8-3.0	229	2.7 ± 0.4	2.0-3.4
Max. LA longitudinal diameter 3ch (cm)	[39]	66	5.5 ± 0.6	4.3-6.7	69	5.4 ± 0.7	4.0-6.8
Max. LA longitudinal diameter 3ch/BSA (cm/m^2^)	[39]	66	3.2 ± 0.4	2.4-4.0	69	3.6 ± 0.5	2.6-4.6
Max. LA antero-posterior diameter 3ch (cm)	[16], [37], [38], [39]	285	3.0 ± 0.5	2.0-4.0	281	3.0 ± 1.0	2.0-3.9
Max. LA antero-posterior diameter 3ch/BSA (cm/m^2^)	[16], [37], [38], [39]	285	1.8 ± 0.5	0.8-2.8	281	2.0 ± 1.0	0.9-3.1
Max. LA longitudinal diameter 4ch (cm)	[16], [38], [39]	226	5.7 ± 0.7	4.3-7.1	229	5.4 ± 0.7	3.9-6.8
Max. LA longitudinal diameter 4ch/BSA (cm/m^2^)	[16], [38], [39]	226	3.1 ± 0.5	2.2-4.1	229	3.4 ± 0.6	2.3-4.5
Max. LA transverse diameter 4ch (cm)	[16], [37], [38], [39]	285	4.2 ± 0.5	3.1-5.2	281	4.0 ± 0.5	3.0-5.1
Max. LA transverse diameter 4ch/BSA (cm/m^2^)	[16], [37], [38], [39]	285	2.2 ± 0.3	1.6-2.9	281	2.5 ± 0.4	1.67- 3.2

*Data are means ± standard deviation. BSA* body surface area, *bSSFP* balanced steady-state free precession, *n* number of subjects, *SD* standard deviation, *LL* lower limit, *UL* upper limit, *Max.* maximal, *LA* left atrial, *2ch* two-chamber view, *3ch* three-chamber view, *4ch* four-chamber view

^a^Left atrial appendage and pulmonary veins excluded

**Age:** The relationship between age and left atrial size is inconsistent in the literature. A positive correlation of age with absolute maximal LA volume was described by Li et al. in 135 healthy Chinese volunteers (23-83 years) and also by Gao et al. for 188 Chinese women (but not in men) [39], [42]. In a subset of 283 healthy subjects (65 ± 9 years) of the MESA cohort, Zemrak et al. did not find a difference with age in LA volume [40]. Similarly, Maceira et al. reported that age was not an independent predictor of LA volume in 120 subjects [38]. The analysis of 9088 subjects from the Healthy Hearts Consortium revealed only minimally lower LA volumes indexed to BSA with higher age [21]. Smaller maximal LA volumes indexed to BSA with higher age were described by Funk et al. for 182 healthy volunteers from Germany (19-76 years) and by Zhuang for 200 healthy Chinese (20-70 years) [16], [41].

**Anthropometric measures:** In a previous study of 120 participants, on average, BSA was higher in males compared to females [38]. The authors have shown that BSA had a significant independent influence on all LA parameters, except for the longitudinal and transverse diameters in the four-chamber view [38].

**Ethnicity:** In large contemporary studies by Zemrak et al. from the MESA cohort of 2576 participants, which included Chinese American, Black, Hispanic, and White ethnicities, researchers found that Hispanic ethnicity was associated with larger LA volumes, while Chinese ethnicity was associated with smaller LA volumes, even after adjusting for allometric measures such as BSA and height [40]. This is likely due to their overall smaller heart size, as previously shown in MESA by Natori et al., who reported lower LV mass and volumes in Chinese Americans compared to other ethnic groups [43]. Data on other ethnicities remain limited, as most studies have predominantly focused on White participants. However, Raisi et al. described only minimal ethnicity differences of LA dimensions between 9088 participants of White, South Asian, Black and Chinese ethnicities from the Healthy Hearts Consortium [21].

**Acquisition technique:** Assessment of LA dimensions typically involves acquiring cine images in the two-, three-, and/or four-chamber views, and occasionally a full short-axis stack. Visualization of the LA may be suboptimal on long-axis images, which are typically focused on the assessment of the ventricles, leading to underestimation of LA volumes [21], [44]. Additional cine images focused on the visualization of the LA might be helpful but require extra scan time [44].

**Post-processing:** SCMR guidelines recommend assessing LA size as either normal or dilated [45]. For quantification, maximal LA volume should be measured at the onset of ventricular systole from the last cine image before mitral valve opening, while minimal LA volume is captured from the first cine image after mitral valve closure. Optional measurements include reporting maximal/minimal LA volumes and indexed for BSA, longitudinal and transverse dimensions, atrial areas from two-, three-, and four-chamber images, and calculating LA ejection and stroke volumes [45].

Endocardial contouring, done manually or semi-automatically, is used to trace LA borders. It should be indicated whether the pulmonary veins and the LA appendage were included or excluded from measurements. LA volumes are commonly calculated using the biplane area-length method or Simpson’s method (if short-axis images are available) and indexed to BSA. In the MESA cohort, Zemrak et al. showed that LA volume index calculated from biplane measurements was only 0.8% smaller than from short-axis imaging using Simpson’s rule, with a strong correlation between the two methods (intraclass correlation coefficient = 0.97) [40]. Also Nacif et al. found a good correlation between the biplane area-length and the Simpson’s method [46].

Linear measurements of LA diameters are taken in two-, three-, and four-chamber views at both end-diastole and end-systole, and LA function is evaluated through volume changes across the cardiac cycle to calculate LA ejection fraction and phasic functions (reservoir, conduit, and contractile phases). Automated tools aid in contouring and three-dimensional (3D) reconstruction, enhancing accuracy and reproducibility.

## 6 Normal values of right atrial dimensions and functions in adults

### 6.1 Influencing factors

**Gender and BSA:** Absolute RA dimensions and volumes are generally larger in men than women. After indexing by body size, differences by gender are generally decreased or eliminated. In Maceira’s study of 120 participants, men had larger absolute RA volumes, diameters, and areas compared to women, except for the transverse diameter in the two-chamber view. However, when indexed to BSA, RA volumes showed no significant sex differences, and multivariable analysis confirmed that sex had no independent influence on any RA parameter [47]. Similar findings were reported in a large study by Petersen et al. from the UK Biobank, which included 804 participants [22]. Specifically, Petersen et al. report that RA maximal volume and stroke volume were significantly larger in males compared to females for absolute values. After indexing for BSA, the difference remained significant for RA maximal volume only. Females had a higher RA ejection fraction compared to males. When normalized for BSA, there were no changes in RA volumes or function with age in either sex.

**Fig. 5:**
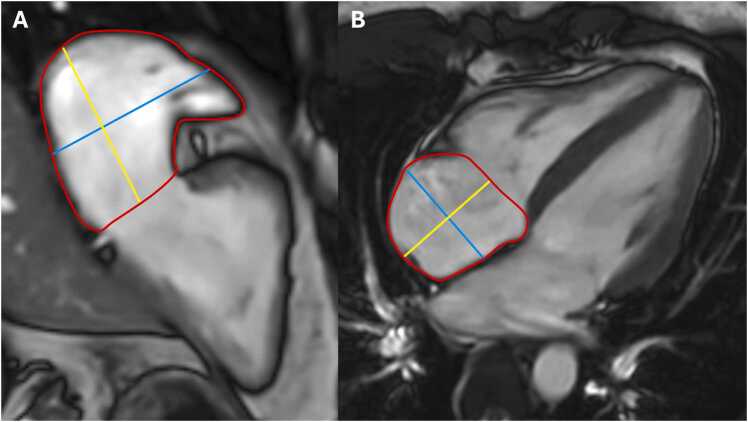
Measurements of maximal right atrial areas (red), transverse diameters (blue), and longitudinal diameters (yellow) obtained on right ventricular two-chamber view (A) and four-chamber view (B) balanced steady-state free precession images at ventricular systole according to reference [47]. The longitudinal parameters (yellow) are obtained from the posterior wall of the right atrium to the center of the tricuspid plane and the transverse diameters (blue) are obtained perpendicular to the longitudinal parameter, at the mid level of the right atrium

**Table 17:** References. Normal values of right atrial dimensions and functions in adults.

First author, year n, male:female Age range Study population	CMR acquisition	Parameter	Post-processing (Figs. 4 and 5)
Maceira, 2013 [47] 60:60 20-80 years Volunteers, UK	• 1.5T, bSSFP • RA 2ch, 4ch • SAX cine stack covering the entire RA	• Maximal RA volume (absolute and indexed to BSA) • Maximal RA area (absolute and indexed to BSA) on a RA 2ch and on a 4ch-view • Maximal RA longitudinal and transverse diameter (absolute and indexed to BSA) on a RA 2ch and on a 4ch-view	• Included the atrial appendage and excluded the cava veins. • Atrial volume analysis included 2 steps: First, delineation of the atrial endocardial border in all planes in all cardiac phases. Second, the systolic descent and twist of the tricuspid valve were calculated from tracking of the valve motion on the long axis cines, and used to correct for increase in atrial volume due to atrioventricular ring descent (using semi-automatic software CMRTools^a^). • The longitudinal diameter measured from the midpoint of the line between the lateral and septal (or superior and inferior in the two-chamber view) insertion of the tricuspid valve to the roof of the right atrium. • Transverse diameter measured perpendicular to the midpoint of the longitudinal diameter.
Le, 2016 [26] 91:89 20-69 years Volunteers, Singapore	• 1.5T, bSSFP • 4ch	• Maximal RA area (absolute and indexed to BSA) on a 4ch-view	• RA area measured at the end of ventricular systole (same frame used to assess LA dimensions) on a 4ch view (using cvi42^b^).
Le Ven, 2016 [11] 196:238 18-36 years Caucasian volunteers, Canada	• 1.5T, bSSFP • SAX cine stack	• Maximal RA volume [RA end-diastolic volume] (absolute and indexed to BSA) • Minimal RA volume [RA end-systolic volume] (absolute and indexed to BSA) • RA stroke volume (absolute and indexed to BSA) • RA ejection fraction	• RA endocardial borders manually traced in atrial end-diastolic and end-systolic phases on contiguous short-axis slices; end-diastole referring to the atrial diastole (maximum volume). • The atrial appendage was included in the total RA volumes. • Volumes were calculated from Simpson’s method (using cvi42 version 3.4.1^b^).
Li, 2017 [39] 66:69 23-83 years Volunteers, China	• 3.0T, bSSFP • SAX, RV 2ch, 4ch	• Maximal RA volume (absolute and indexed to BSA) • Minimal RA volume (absolute and indexed to BSA)	• RA dimensions measured on 4-ch images and RV 2-ch images (Fig. 3). • Maximal RA volume (prior to atrial contraction) and minimal RA volume quantified in SAX and by the bi-plane and mono-plane area-length method (using Qmass, version 7.6^c^).
Zhuang, 2020 [16] 100:100 20-70 years Volunteers, China	• 3.0T, bSSFP • 4ch	• Maximal RA longitudinal and transverse diameter (absolute and indexed to BSA) • RA area at end-systole and end-diastole (absolute and indexed to BSA)	• RA transverse diameter measured perpendicular to the interatrial septum and longitudinal diameter measured perpendicular to the tricuspid valve port. • RA area measured at end-diastole and end-systole of the 4ch-view.
Gao, 2022 [42] 220:188 21-70 years Healthy participants of CMR screening, China	• 1.5T, bSSFP • 4ch	• Maximal and minimal RA volume (indexed to BSA)	• RA endocardial contours were manually tracked on a 4ch-view. • Maximal RA volume measured pre-atrial contraction, and minimal RA volume measured after atrial contraction (using cvi42 version 5.12.1^b^).
Raisi-Estabragh, 2024 [21] 4452:4636 18-83 years Healthy Hearts Consortium^d^	• 1.5T or 3.0T, bSSFP • 4ch	• Maximal RA volume (indexed to BSA and height) • RA ejection fraction	• RA endocardial contours traced on a 4ch-view (using a fully automatic prototype of cvi42 version 5.14^b^).

*n* number of study subjects, *T* Tesla, *bSSFP* balanced steady-state free precession, *RA* right atrial, *2ch* two-chamber view, *4ch* four-chamber view, *SAX* short axis, *BSA* body surface area

^a^Cardiovascular Imaging Solutions, London, UK; ^b^Circle Cardiovascular Imaging Inc., Calgary, Alberta, Canada; ^c^Medis Medical Imaging Systems, Leiden, The Netherlands; ^d^UK Biobank, UK (n = 7672) [22] + SHIP, Germany (Study of Health in Pomerania) (n = 627) [23] + Italy (n = 291) [24] + Germany (n = 190) [25] + Singapore (n = 180) [26] + The Netherlands (n = 128) [27]

**Table 18:** Absolute and indexed (BSA) right atrial volumes and function in men and women for bSSFP imaging.

			Men			Women		
Method	Parameter	References	n	Mean ± SD	LL-UL	n	Mean ± SD	LL-UL
Bi-plane area-length method (RV 2ch, 4ch); RA appendage excluded	Max. RA volume (mL)	[39]	66	65 ± 20	25-105	69	53 ± 14	25-81
	Max. RA volume/BSA (mL/m^2^)	[39]	66	38 ± 12	14-62	69	35 ± 10	15-55
	Min. RA volume (mL)	[39]	66	32 ± 12	8-56	69	23 ± 7	9-73
	Min. RA volume/BSA (mL/m^2^)	[39]	66	19 ± 7	5-33	69	15 ± 5	5-25
	RA ejection fraction (%)	[39]	66	50 ± 9	32-68	69	56 ± 9	38-74
Single-plane area-length method (4ch); RA appendage excluded	Max. RA volume (mL)	[16], [21], [39], [42]	13,286	70 ± 28	14-125	13,842	57 ± 19	20-95
	Max. RA volume/BSA (mL/m^2^)	[16], [21], [39], [42]	13,289	37 ± 14	11-64	13,818	35 ± 10	15-56
	Min. RA volume (mL)	[16], [21], [39], [42]	13,277	41 ± 20	3-80	13,857	33 ± 17	-^a^
	Min. RA volume/BSA (mL/m^2^)	[16], [21], [39], [42]	13,274	22 ± 10	3-41	13,830	20 ± 10	1-40
	RA ejection fraction (%)	[21], [39], [42]	13,066	49 ± 10	30-69	13,592	54 ± 9	35-72
Simpson’s method; RA appendage included	Max. RA volume (mL)	[11], [39], [47]	322	102 ± 26	51-154	367	82 ± 19	46-119
	Max. RA volume/BSA (mL/m^2^)	[11], [39], [47]	322	55 ± 12	31-78	367	51 ± 10	32-70
	Min. RA volume (mL)	[11], [39]	262	49 ± 17	15-82	307	34 ± 11	13-54
	Min. RA volume/BSA (mL/m^2^)	[11], [39]	262	26 ± 8	10-42	307	22 ± 6	9-34
	RASV (ml)	[11]	196	58 ± 16	26-90	238	47 ± 12	23-71
	RASV/BSA (mL/m^2^)	[11]	196	30 ± 8	14-46	238	28 ± 7	14-42
	RA ejection fraction (%)	[11], [39]	262	52 ± 11	31-72	307	57 ± 10	38-76

*Data are means ± standard deviation. BSA* body surface area, *bSSFP* balanced steady-state free-precession, *n* number of subjects, *SD* standard deviation, *LL* lower limit, *UL* upper limit, *RV* right ventricular, *2ch* two-chamber view, *4ch* four-chamber view, *RA* right atrial, *max.* maximal, *min.* minimal, *SV* stroke volume

^a^Limited reliability of the reference interval

**Table 19:** Absolute and indexed (BSA) right atrial volumes and function in men and women by ethnicity for bSSFP imaging using the single-plane area-length method.^a^

			Men			Women		
Parameter	Ethnicity	References	n	Mean ± SD	LL-UL	n	Mean ± SD	LL-UL
Max. RA volume (mL)	Black	[21]	441	91 ± 23	47-135	567	72 ± 19	34-109
	Caucasian	[21]	10,575	89 ± 27	37-141	10,884	68 ± 18	33-104
	Chinese	[16], [21], [39], [42]	812	66 ± 19	29-103	915	55 ± 14	28-81
	South Asian	[21]	948	74 ± 21	34-114	564	60 ± 15	31-89
Max. RA volume/BSA (mL/m^2^)	Black	[21]	441	45 ± 11	23-66	564	39 ± 10	19-58
	Caucasian	[21]	10,581	44 ± 13	18-70	10,872	38 ± 10	18-58
	Chinese	[16], [21], [39], [42]	809	36 ± 10	16-56	915	35 ± 9	18-52
	South Asian	[21]	948	39 ± 11	18-60	561	36 ± 9	18-53
Min. RA volume (mL)	Black	[21]	438	48 ± 15	19-77	567	37 ± 12	13-61
	Caucasian	[21]	10,569	43 ± 17	9-76	10,905	31 ± 12	8-54
	Chinese	[16], [21], [39], [42]	812	40 ± 17	7-73	915	31 ± 16	1-62
	South Asian	[21]	951	36 ± 13	11-62	564	28 ± 10	8-48
Min. RA volume/BSA (mL/m^2^)	Black	[21]	438	23 ± 7	10-37	564	20 ± 6	7-23
	Caucasian	[21]	10,569	21 ± 8	4-38	10,887	17 ± 7	5-30
	Chinese	[16], [21], [39], [42]	812	22 ± 9	4-40	918	20 ± 10	1-39
	South Asian	[21]	948	19 ± 7	6-32	558	16 ± 6	5-28
RA ejection fraction (%)	Black	[21]	435	46 ± 8	31-62	570	49 ± 9	32-66
	Caucasian	[21]	10,473	52 ± 10	32-72	10,764	54 ± 10	36-73
	Chinese	[21], [39], [42]	709	49 ± 9	31-68	812	54 ± 8	38-69
	South Asian	[21]	945	51 ± 9	34-68	555	53 ± 8	37-70

*Data are means ± standard deviation. BSA* body surface area, *bSSFP* balanced steady-state free-precession, *n* number of subjects, *SD* standard deviation, *LL* lower limit, *UL* upper limit, *RA* right atrial, *max.* maximal, *min.* minimal

^a^Measured on a four-chamber view

**Table 20:** Absolute and indexed (BSA) right atrial volumes and function in men and women by age for bSSFP imaging using the single-plane area-length method.^a^

			Men			Women		
Parameter	Age^b^	References	n	Mean ± SD	LL-UL	n	Mean ± SD	LL-UL
Max. RA volume (mL)	20-29	[16], [21], [42]	361	68 ± 26	18-118	333	55 ± 13	29-81
	30-39	[16], [21], [42]	493	70 ± 23	25-114	460	55 ± 13	30-81
	40-49	[16], [21], [42]	1554	73 ± 29	16-131	1752	59 ± 20	20-97
	50-59	[16], [21], [42]	3502	72 ± 28	18-127	3924	59 ± 20	21-98
	60-69	[16], [21], [42]	3248	68 ± 31	7-129	3674	60 ± 20	22-99
	≥70	[21]	4062	87 ± 27	35-140	3630	68 ± 19	30-105
Max. RA volume/BSA (mL/m^2^)	20-29	[16], [21], [42]	361	36 ± 12	13-59	333	34 ± 8	19-49
	30-39	[16], [21], [42]	490	37 ± 10	17-57	460	34 ± 7	20-48
	40-49	[16], [21], [42]	1554	38 ± 14	11-65	1749	35 ± 10	16-55
	50-59	[16], [21], [42]	3493	37 ± 13	12-62	3924	35 ± 10	15-55
	60-69	[16], [21], [42]	3257	37 ± 15	8-66	3668	36 ± 11	15-57
	≥70	[21]	4068	44 ± 14	17-71	3615	39 ± 11	17-61
Min. RA volume (mL)	20-29	[16], [21], [42]	361	40 ± 20	1-80	333	29 ± 14	3-56
	30-39	[16], [21], [42]	493	42 ± 17	8-76	460	37 ± 25	-^c^
	40-49	[16], [21], [42]	1551	45 ± 18	9-81	1752	34 ± 14	6-61
	50-59	[16], [21], [42]	3502	46 ± 18	10-81	3930	39 ± 20	-^c^
	60-69	[16], [21], [42]	3248	44 ± 21	3-85	3674	34 ± 14	7-61
	≥70	[21]	4056	41 ± 18	6-76	3639	30 ± 12	6-54
Min. RA volume/BSA (mL/m^2^)	20-29	[16], [21], [42]	361	21 ± 10	1-42	333	19 ± 9	1-36
	30-39	[16], [21], [42]	490	22 ± 9	5-40	460	23 ± 16	-^c^
	40-49	[16], [21], [42]	1551	23 ± 9	5-41	1749	20 ± 8	4-36
	50-59	[16], [21], [42]	3508	24 ± 9	6-42	3924	23 ± 12	-^c^
	60-69	[16], [21], [42]	3248	24 ± 11	3-45	3674	20 ± 8	5-36
	≥70	[21]	4050	21 ± 9	3-38	3621	17 ± 7	3-31
RA ejection fraction (%)	20-29	[21], [42]	341	52 ± 13	27-77	313	57 ± 11	37-78
	30-39	[21], [42]	473	50 ± 11	29-71	440	57 ± 12	34-80
	40-49	[21], [42]	1534	49 ± 10	30-68	1726	52 ± 10	34-71
	50-59	[21], [42]	3467	48 ± 9	30-66	3865	51 ± 9	33-68
	60-69	[21], [42]	3207	49 ± 10	29-69	3618	53 ± 9	35-71
	≥70	[21]	3978	53 ± 11	31-74	3561	55 ± 10	36-75

*Data are means ± standard deviation. BSA* body surface area, *bSSFP* balanced steady-state free-precession, *n* number of subjects, *SD* standard deviation, *LL* lower limit, *UL* upper limit, *RA* right atrial, *max.* maximal, *min.* minimal

^a^Measured on a four-chamber view, ^b^in years, ^c^limited reliability of the reference interval

**Table 21:** Absolute and indexed (BSA) right atrial area and diameter in men and women for bSSFP imaging.

		Men				Women	
Parameter	References	n	Mean ± SD	LL-UL	n	Mean ± SD	LL-UL
Max. RA area 2ch (cm^2^)^a, b^	[47]	60	23 ± 4	15-31	60	21 ± 4	13-29
Max. RA area 2ch/BSA (cm^2^/m^2^)^a, b^	[47]	60	12 ± 2	8-16	60	12 ± 2	8-16
Max. RA area 4ch (cm^2^)	[16], [26], [47]	251	21 ± 5	11-31	249	18 ± 4	11-25
Max. RA area 4ch/BSA (cm^2^/m^2^)	[16], [26], [47]	251	11 ± 3	6-16	249	11 ± 2	6-15
Max. RA longitudinal diameter 2ch (cm)	[39], [47]	126	5.5 ± 0.7	4.2-6.7	129	5.1 ± 0.6	3.9-6.3
Max. RA longitudinal diameter 2ch/BSA (cm/m^2^)	[39], [47]	126	3.0 ± 0.4	2.3-3.7	129	3.2 ± 0.5	2.2-4.1
Max. RA transverse diameter 2ch (cm)^a^	[39], [47]	126	4.2 ± 0.9	2.6-5.9	129	4.1 ± 0.9	2.4-5.8
Max. RA transverse diameter 2ch/BSA (cm/m^2^)^a^	[39], [47]	126	2.3 ± 0.5	1.3-3.3	129	2.6 ± 0.6	1.5-3.7
Max. RA longitudinal diameter 4ch (cm)	[16], [39], [47]	226	5.2 ± 0.7	3.7-6.6	229	4.9 ± 0.7	3.6-6.3
Max. RA longitudinal diameter 4ch/BSA (cm/m^2^)	[16], [39], [47]	226	2.8 ± 0.5	1.8-3.8	229	3.1 ± 0.5	2.1-4.1
Max. RA transverse diameter 4ch (cm)	[16], [39], [47]	226	4.6 ± 0.6	3.4-5.9	229	4.1 ± 0.7	2.8-5.5
Max. RA transverse diameter 4ch/BSA (cm/m^2^)	[16], [39], [47]	226	2.5 ± 0.4	1.8-3.3	229	2.6 ± 0.4	1.7-3.4

*Data are means ± standard deviation. BSA* body surface area, *bSSFP* balanced steady-state free-precession, *n* number of subjects, *SD* standard deviation, *LL* lower limit, *UL* upper limit, *Max.* maximal, *RA* right atrial, *2ch* two-chamber view, *4ch* four-chamber view

^a^Right atrial appendage included, ^b^cava veins excluded

In contrast, Le Ven et al.'s study of 434 participants found that sex was independently associated with RA end-diastolic and end-systolic volumes, with males having larger values, even after normalizing to BSA [11]. In their multivariable analysis, BSA was independently associated with all RA parameters, while sex was independently associated with RA-EDV and RA-ESV. The discrepancy between the role of sex after normalizing to BSA may be due to differences in post-processing methods, as Maceira used long-axis views, whereas Le Ven used the short-axis stack [11], [47].

**Age:** Maceira et al., in their study of 120 participants, reported RA volumes showed no significant changes with age when indexed to BSA [47]. Similarly Le Ven in their study of 434 participants showed that age was not independently associated with any RA parameter [11]. In 408 healthy Chinese, Gao et al. described a positive correlation of absolute maximal RA volume only in women but not in men [42]. They also found a lower RV ejection fraction in men and women of older age groups compared to younger ages [42].

**Ethnicity:** Data on ethnic trends are limited. In the most recent study by Raisi-Estabragh et al., involving 9088 participants (51% female) from the Healthy Hearts Consortium, which included White, South Asian, Black, Chinese, and Mixed/other ethnicities, minimal differences in RA metrics were observed across ethnic groups. These findings were consistent across different age groups and between males and females [21].

**Acquisition technique:** Long-axis cine bSSFP images acquired in the four-chamber view and the RV two-chamber view and occasionally a short-axis stack covering the entire RA are used to measure and calculate RA dimensions. Similar as for the LA, long-axis images are frequently optimized to visualize the ventricles and might display the RA suboptimal.

**Post-processing:** SCMR guidelines recommend assessing RA size as either normal or dilated, with several optional measurements [45]. In the majority of studies, RA volume is calculated by the single(mono)-plane area-length method based on a four-chamber view. In other publications, the authors used the bi-plane area length method based on a four-chamber view and a RV two-chamber view or the Simpson’s method on a short-axis cine stack covering the entire RA to calculate RV volume. It should be mentioned if the RA appendage was included or excluded from measurements.

## 7 Normal volumes, mass, and function of the left and right ventricle in children

### 7.1 Influencing factors

**Gender:** The LV and RV end-diastolic volumes and myocardial masses for males were greater than females throughout the pediatric age range, but differences are most important in adolescent patients [50], [52], [54], [55]. One study found a small difference in LVEF for males versus females [48], but this is not a consistent finding [52], [55].

**Table 22:** References. Normal volumes, mass, and function of the left and right ventricle in children.

First author, year n, male:female Age range Study population	CMR acquisition	Parameter	Post-processing
van der Ven, 2019 [48] 68:73 0.6-18.5 years Volunteers; Germany, Switzerland, Netherlands	• 1.5T; short-axis bSSFP	• LVEDV, LVESV, LVSV, LVEF, LV mass, RVEDV, RVESV, RVSV, RVEF, RV mass	• Processed with Medis software.^a^ • According to 2013 Society for Cardiovascular Magnetic Resonance consensus [49]. • Epicardial and myocardial borders were manually contoured in end-diastolic and end-systolic phases. End-diastolic and end-systolic phases were the same for both ventricles. • Papillary muscles and major trabeculae were included in the myocardial mass. • The septum was included in the left ventricular mass. • RV contours were drawn along major trabeculae if in continuity with the ventricular wall.
Olivieri, 2020 [50] (ref. only) 70:79 21 days-12 years Healthy volunteers and infants of different ethnicities to evaluate for a vascular ring, USA	• 1.5T; short-axis bSSFP; free-breathing; non-commercial motion corrected re-binned sequence	• LVEDV, LVESV, LVSV, LV mass, LV cardiac output, RVEDV, RVESV, RVSV, RV cardiac output	• Analysis with Medis software.^a^ • Papillary muscles and trabeculations included in the blood pool. • Data was normalized to BSA and sex. • Z-score equations provided.
Cardinal, 2021 [51] (ref. only) 189:183 16-23.9 years Subjects with a clinical indication for CMR that was normal or with conditions not affecting volumes and function, Canada	• 1.5T; short-axis bSSFP	• LVEDV, LVESV, LVSV, LVEF, RVEDV, RVESV, RVSV, RVEF	• Processed with a variety of systems. Data pulled from clinical reports. • LV papillary muscles and trabeculations were included in the myocardial mass. • RV trabeculations and papillary muscles were included in the ventricular volume. • Z-score equations were calculated for all variables.
Krupickova, 2021 [52] (ref. only) 97:64 6-18 years Children with scans for clinical reasons but without cardiovascular disease and normal CMR, UK and Germany	• 1.5T, short-axis bSSFP	• LVEDV, LVESV, LVSV, LVEF, LV mass, RVEDV, RVESV, RVSV, RVEF, RV mass	• Analysis with cvi42 software.^b^ • Papillary muscles were excluded from LV mass. • LV mass was quantified at diastole. • Since BSA was a determinant for LV mass according to regression analysis, centiles were calculated by BSA.
Jhaveri, 2023 [53] (ref. only) 13:7 9-56 h Healthy newborns	• 3T; short-axis bSSFP; free-breathing	• LVEDV, LVESV, LVSV, LVEF, RVEDV, RVESV, RVSV, RVEF	• Analysis with cvi42 software.^b^ • LV papillary muscles were contoured independently and included in the myocardial mass.
Real, 2023 [54] (ref. only) 59:64 15-18 years Participants of the EnIGMA project without evidence or history of cardiovascular disease, Spain	• 3T; short-axis bSSFP	• LVEDV, LVESV, LVSV, LVEF, LVED mass, RVEDV, RVESV, RVSV, RVEF	• Analysis with IntelliSpace Portal software.^c^ • Papillary muscles and trabeculations were included in the ventricular volume.
Voges, 2023 [55] 101:53 (13.9 ± 2.8) years^d^ Children with scans for clinical reasons but without cardiovascular disease and normal CMR, UK and Germany	• 1.5T; bSSFP	• LVEDV, LVESV, LVSV, LVEF, LVED mass, LVES mass, RVEDV, RVESV, RVSV, RVEF, RVED mass; RVES mass	• Analysis with cvi42 software.^b^ • Two different contouring methods: (1) using semi-automated contour drawing, the papillary muscles and trabeculations were included in the blood volume and (2) using a threshold technique, the papillary muscles and trabeculations were included in the myocardial mass.

*n* number of study subjects, *T* Tesla, *bSSFP* balanced steady-state free precession, *LV* left ventricular, *EDV* end-diastolic volumen, *ESV* end-systolic volume, *SV* stroke volume, *EF* ejection fraction, *RV* right ventricular, *BSA* body surface area, *EnIGMA* early imaging markers of unhealthy lifestyles in adolescents

^a^Medis Medical Imaging Systems, Leiden, The Netherlands; ^b^Circle Cardiovascular Imaging Inc., Calgary, Alberta, Canada; ^c^Koninklijke Philips N.V., Amsterdam, The Netherlands; ^d^mean ± SD (age range not provided in original publication)

**Table 23:** Centiles (in mL) of normal left ventricular volumes (excluding papillary muscles and trabeculation) by body height for short-axis bSSFP in boys according to reference [55].

	LVEDV							LVESV							LVSV						
Height (cm)	5^th^	10^th^	25^th^	50^th^	75^th^	90^th^	95^th^	5^th^	10^th^	25^th^	50^th^	75^th^	90^th^	95^th^	5^th^	10^th^	25^th^	50^th^	75^th^	90^th^	95^th^
110	45	47	50	54	59	64	68	14	14	16	18	21	24	26	27	29	33	37	41	45	48
120	52	55	59	64	70	76	80	16	17	19	22	25	29	31	31	34	38	42	48	52	55
130	61	64	69	75	82	90	95	19	21	23	26	30	35	38	36	39	44	49	55	61	64
140	71	75	81	89	97	107	112	23	25	28	32	37	42	46	42	45	51	57	64	70	74
150	82	87	95	105	115	126	133	27	29	33	39	45	51	55	49	52	59	66	74	81	86
160	95	101	111	123	137	149	157	32	35	40	47	54	62	67	57	61	68	76	85	94	99
170	110	118	130	146	162	177	186	38	42	48	56	66	75	81	66	70	78	88	98	108	114
180	127	136	153	172	191	209	220	44	49	58	68	79	90	98	76	82	91	102	114	125	132
190	145	158	179	203	226	247	260	52	58	69	82	96	110	118	88	95	105	118	132	145	153
200	164	181	209	239	267	292	306	60	68	83	99	116	132	142	102	109	122	136	152	167	176

*Data are presented in centiles. bSSFP* balanced steady-state free precession, *LV* left ventricular, *EDV* end-diastolic volume, *ESV* end-systolic volume, *SV* stroke volume

**Table 24:** Centiles (in mL) of normal left ventricular volumes (including papillary muscles and trabeculation) by body height for short-axis bSSFP in boys according to reference [55].

	LVEDV							LVESV							LVSV						
Height (cm)	5^th^	10^th^	25^th^	50^th^	75^th^	90^th^	95^th^	5^th^	10^th^	25^th^	50^th^	75^th^	90^th^	95^th^	5^th^	10^th^	25^th^	50^th^	75^th^	90^th^	95^th^
110	47	49	53	58	66	79	93	16	17	19	21	24	27	29	19	21	25	28	32	36	38
120	55	58	62	69	78	93	108	19	21	23	26	29	33	36	28	30	34	39	43	47	50
130	65	68	73	81	92	106	119	23	25	27	31	35	40	43	36	39	44	49	55	59	62
140	75	79	86	95	107	120	130	27	29	33	38	43	49	52	44	47	53	59	66	72	75
150	88	92	101	112	124	138	147	33	35	40	45	52	59	64	51	55	62	70	77	84	88
160	102	108	119	132	145	158	167	39	42	48	55	63	72	77	59	64	71	80	89	96	101
170	118	126	140	155	170	183	191	45	50	57	66	77	87	93	67	72	81	90	100	108	114
180	136	147	164	182	198	212	220	53	59	69	80	93	105	113	76	81	90	101	111	120	126
290	157	172	193	214	232	247	255	63	70	82	97	113	127	137	84	90	100	111	122	132	138
200	181	201	228	251	271	271	295	73	82	98	117	136	154	165	93	99	110	121	133	143	150

*Data are presented in centiles. bSSFP* balanced steady-state free precession, *LV* left ventricular, *EDV* end-diastolic volume, *ESV* end-systolic volume, *SV* stroke volume

**Table 25:** Centiles (in mL) of normal left ventricular volumes (excluding papillary muscles and trabeculation) by body height for short-axis bSSFP in girls according to reference [55].

	LVEDV							LVESV							LVSV						
Height (cm)	5^th^	10^th^	25^th^	50^th^	75^th^	90^th^	95^th^	5^th^	10^th^	25^th^	50^th^	75^th^	90^th^	95^th^	5^th^	10^th^	25^th^	50^th^	75^th^	90^th^	95^th^
120	21	28	39	51	64	75	81	14	15	18	21	24	27	29	14	17	23	29	36	41	45
130	38	44	55	67	78	89	95	18	20	23	27	30	34	36	23	27	33	39	46	52	55
140	55	61	71	82	93	103	109	22	25	28	33	37	40	43	32	36	42	49	56	63	66
150	72	78	87	98	108	118	124	27	29	33	38	43	47	50	41	45	52	59	67	74	78
160	89	94	103	113	123	132	138	30	33	38	44	49	54	57	50	54	62	69	77	84	89
170	105	110	119	129	138	147	152	34	37	43	49	56	61	65	59	64	71	79	88	95	100
180	122	127	135	144	153	162	167	37	41	48	55	62	69	73	68	73	81	89	98	106	111

*Data are presented in centiles. bSSFP* balanced steady-state free precession, *LV* left ventricular, *EDV* end-diastolic volume, *ESV* end-systolic volume, *SV* stroke volume

**Table 26:** Centiles (in mL) of normal left ventricular volumes (including papillary muscles and trabeculation) by body height for short-axis bSSFP in girls according to reference [55].

	LVEDV							LVESV							LVSV						
Height (cm)	5^th^	10^th^	25^th^	50^th^	75^th^	90^th^	95^th^	5^th^	10^th^	25^th^	50^th^	75^th^	90^th^	95^th^	5^th^	10^th^	25^th^	50^th^	75^th^	90^th^	95^th^
120	17	25	39	55	71	85	94	13	16	21	26	31	36	39	14	17	23	29	35	40	44
130	37	44	57	72	86	99	107	19	22	27	33	38	43	46	23	26	32	39	46	52	55
140	56	63	75	89	102	114	121	25	28	34	39	45	50	53	32	36	42	49	56	63	67
150	75	82	93	105	118	129	135	31	35	40	46	52	57	60	41	45	52	59	67	74	78
160	95	101	111	122	133	144	150	37	41	46	52	59	64	68	49	54	61	70	78	85	90
170	114	119	129	139	149	159	164	43	47	53	59	66	71	75	58	63	71	80	89	97	102
180	132	138	146	156	165	174	179	49	53	59	66	72	79	82	67	72	80	90	100	108	113

*Data are presented in centiles. bSSFP* balanced steady-state free precession, *LV* left ventricular, *EDV* end-diastolic volume, *ESV* end-systolic volume, *SV* stroke volume

**Table 27:** Centiles (in g) of normal left ventricular end-diastolic mass (including papillary muscles) by body height for short-axis bSSFP in boys and girls according to reference [55].

	Boys							Girls						
Height (cm)	5^th^	10^th^	25^th^	50^th^	75^th^	90^th^	95^th^	5^th^	10^th^	25^th^	50^th^	75^th^	90^th^	95^th^
110	27	28	31	35	39	43	46	-	-	-	-	-	-	-
120	32	33	37	41	47	52	55	18	22	29	37	45	52	57
130	37	40	44	50	56	62	66	27	31	39	48	56	64	69
140	44	47	53	59	67	74	79	35	40	49	58	68	76	81
150	52	56	63	71	80	89	95	43	49	58	69	79	88	94
160	62	67	75	85	96	107	113	51	57	68	79	91	101	107
170	73	79	90	102	115	128	136	59	66	77	90	102	113	120
180	86	94	107	122	138	153	163	66	74	86	100	114	126	133
190	101	111	128	146	166	184	194	-	-	-	-	-	-	-
200	119	131	152	175	198	220	232	-	-	-	-	-	-	-

*Data are presented in centiles. bSSFP* balanced steady-state free precession

**Table 28:** Centiles (in g) of normal left ventricular end-diastolic mass (excluding papillary muscles) by body height for short-axis bSSFP in boys and girls according to reference [55].

	Boys							Girls						
Height (cm)	5^th^	10^th^	25^th^	50^th^	75^th^	90^th^	95^th^	5^th^	10^th^	25^th^	50^th^	75^th^	90^th^	95^th^
110	24	26	29	32	35	37	39	-	-	-	-	-	-	-
120	28	31	34	38	41	45	47	20	23	28	34	39	44	47
130	34	36	40	45	50	54	56	27	31	36	43	49	55	58
140	40	43	48	54	60	65	68	34	38	45	52	59	65	69
150	47	51	57	64	72	78	82	41	45	53	61	69	76	81
160	56	60	68	77	86	94	99	48	53	61	70	79	87	92
170	66	71	81	92	103	113	120	54	59	69	79	89	98	104
180	78	85	96	110	124	137	145	60	66	76	88	100	110	116
190	92	100	115	131	149	165	175	-	-	-	-	-	-	-
200	108	118	136	156	178	199	212	-	-	-	-	-	-	-

*Data are presented in centiles. bSSFP* balanced steady-state free precession

**Table 29:** Centiles (in mL) of normal right ventricular volumes (excluding papillary muscles and trabeculation) by body height for short-axis bSSFP in boys according to reference [55].

	RVEDV							RVESV							RVSV						
Height (cm)	5^th^	10^th^	25^th^	50^th^	75^th^	90^th^	95^th^	5^th^	10^th^	25^th^	50^th^	75^th^	90^th^	95^th^	5^th^	10^th^	25^th^	50^th^	75^th^	90^th^	95^th^
110	38	40	44	49	54	59	63	12	13	15	18	20	22	24	15	18	22	26	31	35	38
120	44	47	52	58	64	71	75	14	16	19	21	24	27	29	22	25	29	35	40	45	47
130	52	56	62	69	77	84	89	17	19	22	26	30	33	35	29	32	37	43	49	55	58
140	62	66	73	82	91	100	106	21	23	27	32	36	40	43	36	39	45	52	59	65	69
150	73	78	87	97	109	119	126	25	28	33	38	44	49	52	42	46	54	61	69	76	80
160	86	92	103	116	129	142	150	30	33	40	46	53	60	64	50	55	63	72	80	89	93
170	101	109	122	138	154	169	179	36	40	48	56	65	73	77	58	63	73	83	93	102	108
180	119	129	145	164	183	201	213	43	49	58	68	79	88	94	66	72	83	95	106	117	123
190	140	152	172	195	218	240	253	52	58	70	83	96	108	115	74	81	93	106	120	132	139
200	165	179	204	231	259	285	300	62	70	84	100	116	131	139	80	89	102	118	133	147	155

*Data are presented in centiles. bSSFP* balanced steady-state free precession, *RV* right ventricular, *EDV* end-diastolic volume, *ESV* end-systolic volume, *SV* stroke volume

**Table 30:** Centiles (in mL) of normal right ventricular volumes (including papillary muscles and trabeculation) by body height for short-axis bSSFP in boys according to reference [55].

	RVEDV							RVESV							RVSV						
Height (cm)	5^th^	10^th^	25^th^	50^th^	75^th^	90^th^	95^th^	5^th^	10^th^	25^th^	50^th^	75^th^	90^th^	95^th^	5^th^	10^th^	25^th^	50^th^	75^th^	90^th^	95^th^
110	46	48	51	55	62	72	81	16	17	19	22	25	29	32	26	28	31	35	41	49	56
120	55	57	61	66	74	85	95	20	21	23	27	31	35	39	30	32	36	41	47	55	62
130	64	67	72	79	89	101	111	24	26	29	33	38	43	47	35	38	42	47	54	62	68
140	76	79	86	95	106	119	129	29	31	35	40	46	53	57	41	44	49	55	63	71	77
150	89	94	102	113	127	141	151	35	38	43	49	56	64	70	48	51	57	65	73	81	87
160	104	110	121	135	151	167	177	42	46	52	60	69	79	85	55	59	67	75	85	93	99
170	121	130	144	162	180	198	209	51	55	63	73	85	96	103	64	69	78	88	98	107	113
180	140	152	172	193	215	235	247	61	67	77	90	104	117	126	74	80	91	103	114	124	129
190	160	177	204	231	257	279	292	73	81	94	110	127	143	153	85	93	106	120	132	143	149
200	180	205	241	276	306	331	345	88	97	114	134	155	174	186	98	109	124	140	153	165	171

*Data are presented in centiles. bSSFP* balanced steady-state free precession, *RV* right ventricular, *EDV* end-diastolic volume, *ESV* end-systolic volume, *SV* stroke volume

**Table 31:** Centiles of normal right ventricular volumes (excluding papillary muscles and trabeculation) by body height for short-axis bSSFP in girls according to reference [55].

	RVEDV							RVESV							RVSV						
Height (cm)	5^th^	10^th^	25^th^	50^th^	75^th^	90^th^	95^th^	5^th^	10^th^	25^th^	50^th^	75^th^	90^th^	95^th^	5^th^	10^th^	25^th^	50^th^	75^th^	90^th^	95^th^
120	24	28	36	45	54	62	67	20	20	21	21	21	22	22	10	13	18	23	28	33	36
130	38	43	51	60	70	78	83	23	24	25	26	28	29	30	19	22	27	33	39	45	48
140	52	57	66	76	85	94	100	24	26	29	32	35	38	39	27	31	37	43	50	56	59
150	66	71	80	91	101	110	116	24	27	32	37	42	47	50	36	40	46	53	61	67	71
160	79	85	95	106	117	127	133	27	30	36	42	49	54	58	44	49	56	64	72	79	83
170	93	99	110	121	133	143	149	33	36	42	48	54	59	62	53	57	65	74	83	90	95
180	107	113	124	136	149	160	166	41	44	48	53	57	62	64	61	66	74	84	94	102	107

*Data are presented in centiles. bSSFP* balanced steady-state free precession, *LV* left ventricular, *EDV* end-diastolic volume, *ESV* end-systolic volume, *SV* stroke volume, *RV* right ventricular

**Table 32:** Centiles (in mL) of normal right ventricular volumes (including papillary muscles and trabeculation) by body height for short-axis bSSFP in girls according to reference [55].

	RVEDV							RVESV							RVSV						
Height (cm)	5^th^	10^th^	25^th^	50^th^	75^th^	90^th^	95^th^	5^th^	10^th^	25^th^	50^th^	75^th^	90^th^	95^th^	5^th^	10^th^	25^th^	50^th^	75^th^	90^th^	95^th^
120	26	32	42	54	65	75	81	21	22	22	23	24	24	25	15	18	23	28	34	38	41
130	43	49	60	71	83	94	100	26	27	29	31	33	35	36	24	27	32	38	45	50	53
140	60	66	77	89	102	112	119	29	31	35	39	43	47	50	32	36	42	49	56	62	66
150	77	83	95	107	120	131	138	30	33	40	47	54	61	65	40	44	51	59	67	74	78
160	94	101	112	125	138	150	157	36	40	47	55	63	70	75	48	53	61	69	78	86	91
170	110	118	130	143	156	168	176	48	51	57	63	70	75	79	56	61	70	80	90	98	104
180	127	135	147	161	175	187	195	62	64	67	71	75	79	81	63	69	79	90	101	111	117

*Data are presented in centiles. bSSFP* balanced steady-state free precession, *LV* left ventricular, *EDV* end-diastolic volume, *ESV* end-systolic volume, *SV* stroke volume, *RV* right ventricular

**Table 33:** Centiles (in g) of normal right ventricular end-diastolic mass (including papillary muscles) by body height for short-axis bSSFP in boys and girls according to reference [55].

	Boys							Girls						
Height (cm)	5^th^	10^th^	25^th^	50^th^	75^th^	90^th^	95^th^	5^th^	10^th^	25^th^	50^th^	75^th^	90^th^	95^th^
110	14	15	16	19	22	25	28	-	-	-	-	-	-	-
120	16	17	19	22	25	29	32	10	12	14	17	20	23	25
130	19	20	23	26	30	34	37	14	16	19	23	26	29	31
140	22	24	26	30	35	40	43	18	20	24	28	32	36	38
150	26	28	31	35	41	46	50	22	25	29	33	38	42	45
160	30	33	36	42	48	54	59	26	29	33	39	44	49	52
170	36	38	43	49	56	63	68	29	32	38	44	50	55	59
180	42	45	50	57	65	74	80	32	36	42	49	56	63	66
190	49	53	59	67	77	86	93	-	-	-	-	-	-	-
200	57	61	69	79	90	101	108	-	-	-	-	-	-	-

*Data are presented in centiles. bSSFP* balanced steady-state free precession

**Table 34:** Centiles (in g) of normal right ventricular end-diastolic mass (excluding papillary muscles) by body height for short-axis bSSFP in boys and girls according to reference [55].

	Boys							Girls						
Height (cm)	5^th^	10^th^	25^th^	50^th^	75^th^	90^th^	95^th^	5^th^	10^th^	25^th^	50^th^	75^th^	90^th^	95^th^
110	13	14	16	18	19	21	22	-	-	-	-	-	-	-
120	10	11	13	15	17	18	20	9	10	11	13	15	16	17
130	11	12	14	16	18	20	22	11	12	14	16	18	20	21
140	16	17	19	22	24	26	28	13	14	17	19	21	23	24
150	18	19	22	25	27	30	31	15	17	19	22	25	27	29
160	21	22	25	28	31	34	35	18	20	23	26	29	32	34
170	26	27	31	34	37	41	42	21	23	27	30	34	37	39
180	30	32	35	39	43	46	48	24	27	31	35	39	43	46
190	31	33	37	41	46	49	52	-	-	-	-	-	-	-
200	30	33	37	42	47	51	54	-	-	-	-	-	-	-

*Data are presented in centiles. bSSFP* balanced steady-state free precession

**Age:** All studies showed volumes and masses increase with age and body surface area. The change in volume decelerates as patients age [50], [52]. There was no relationship between LV or RV EDV with gestational age [53].

**Anthropometric measures:** Myocardial volumes, masses, and wall thickness are dependent on BSA, weight, and height [50], [52], [53], [54], [55]. Using a non-linear predictor or Z-score approach is better for normalization than simple BSA normalization. The Lambda-Mu-Sigma model can be used to model variables versus size metrics [52]. Using Z-scores improves the utility of normalization when indexing by BSA [50], [51]. In young children (mean age 14 years), height showed somewhat stronger associations with RV and LV cardiac parameters compared to BSA [55].

**Acquisition technique:** Using free-breathing acquisition versus breath holding may have important differences for volume and mass estimates, but has not been tested explicitly in any of these references [50].

**Post-processing:** Including the papillary muscles, trabeculations, and moderator band in the ventricular volume results in larger LV and RV volume estimates [55]. Conversely, including them in the mass estimate results in larger ED and ES mass and ejection fractions. Both methods showed similar excellent intraobserver and interobserver agreement [55].

## 8 Normal volumes of the left and right atrium in children

### 8.1 Influencing factors

**Gender:** Left atrial and right atrial volumes and function showed no sex difference [54]. One study found significantly larger left atrial volumes in males [56], but the indexed left atrial volume showed no difference. One study showed increased growth rate of the atria for males [56].

**Table 35:** References. Normal volumes of the left and right atrium in children.

First author, year n, male: female Age range Study population	CMR acquisition	Parameter	Post-processing
Sarikouch, 2011 [56] 56:59 4-20 years Caucasian volunteers, Germany	• 1.5T; axial bSSFP	• Maximum and minimum LA and RA volumes; LA and RA emptying fraction	• Simpson’s method using semi-automated contours drawn on a stack of axial images. • Pulmonary veins, SVC, suprahepatic IVC were excluded. • Atrial appendages and the AV valve recesses were included in the volume. • Reference centiles curves were calculated by the LMS method, with L = Lambda (skewness of the distribution), M = Mu (median), S = Sigma (variance)
Real, 2023 [54] (ref. only) 59:64 15-18 years Participants of the EnIGMA project without evidence or history of cardiovascular disease, Spain	• 3T; 4ch and 2ch bSSFP	• Maximum and minimum LA and RA volumes, LA pre-atrial contraction volume; LA emptying fraction, passive emptying fraction, and active emptying fraction	• LA: biplane area-length method. • RA: 4ch single plane area-length method. • LA contour excluded the pulmonary veins, appendage, and mitral valve recess.
Voges, 2023 [57] 103:52 4-18 years Children with scans for clinical reasons but without cardiovascular disease and normal CMR, UK and Germany	• 1.5T; 4ch and 2ch bSSFP	• Maximum and minimum LA and RA volumes	• LA and RA volumes calculated using 3 methods: 2ch and 4ch area-length and biplane. • LA contour excluding the pulmonary veins, appendage, and mitral valve recess.

*n* number of study subjects, *T* Tesla, *bSSFP* balanced steady-state free precession, *4ch* four chamber, *2ch* two chamber, *LA* left atrial, *RA* right atrial, *SVC* superior vena cava, *IVC* inferior vena cava, *AV* atrioventricular, *EnIGMA* early imaging markers of unhealthy lifestyles in adolescents

**Table 36:** Normal maximal left atrial volume^a^ normalized to BSA in boys and girls; LMS parameters to calculate z-scores and centiles by age according to reference [56].

	Boys										Girls									
	LMS-parameters			Centiles (mL/m^2^)							LMS-parameters			Centiles (mL/m^2^)						
Age^b^	L	M	S	3^rd^	10^th^	25^th^	50^th^	75^th^	90^th^	97^th^	L	M	S	3^rd^	10^th^	25^th^	50^th^	75^th^	90^th^	97^th^
4	-	-	-	-	-	-	-	-	-	-	−1.100	37.566	0.248	22	27	31	34	38	41	44
5	-	-	-	-	-	-	-	-	-	-	−0.956	38.333	0.242	23	28	32	36	40	43	46
6	1.378	36.715	0.263	14	23	30	37	43	49	55	−0.717	39.568	0.234	25	30	34	39	42	46	50
7	1.378	38.610	0.246	17	25	32	39	45	51	56	−0.478	40.739	0.225	26	32	36	41	45	49	53
8	1.378	40.291	0.229	20	27	34	40	46	52	57	−0.239	41.934	0.217	28	33	38	43	47	51	55
9	1.378	41.762	0.212	22	29	36	42	48	53	58	0.000	43.072	0.208	28	34	39	44	48	52	56
10	1.378	43.375	0.197	25	31	38	43	49	54	59	0.239	43.953	0.199	28	34	39	44	48	52	56
11	1.378	45.120	0.183	27	34	39	45	51	56	61	0.478	44.548	0.191	29	34	39	44	48	53	57
12	1.378	46.671	0.171	29	35	41	47	52	57	62	0.717	45.080	0.182	29	35	40	45	49	53	58
13	1.378	47.784	0.161	31	37	43	48	53	58	62	0.956	45.636	0.173	30	35	41	45	50	54	59
14	1.378	48.331	0.152	33	38	43	48	53	58	62	1.195	46.118	0.165	30	36	41	46	51	56	60
15	1.378	48.581	0.142	34	39	44	49	53	57	62	1.434	46.070	0.156	30	36	42	47	51	56	60
16	1.378	49.112	0.131	36	40	45	49	53	57	61	1.673	45.343	0.148	30	35	41	45	50	55	59
17	1.378	50.353	0.120	38	42	46	50	54	58	62	1.912	44.258	0.139	29	34	39	44	48	53	57
18	1.378	52.583	0.111	40	45	49	53	56	60	64	2.151	43.116	0.130	27	33	38	42	47	51	55
19	1.378	55.860	0.103	44	48	52	56	60	63	67	**-**	**-**	**-**	-	-	-	-	-	-	-
20	1.378	59.928	0.097	48	52	56	60	64	67	71	**-**	**-**	**-**	-	-	-	-	-	-	-

*Values represent LMS parameters and centiles. BSA* body surface area, *LMS* L = Lambda (skewness of the distribution), M = Mu (median), S = Sigma (variance)

^a^Quantified by a stack of axial cine balanced steady-state free precession images, excluding the pulmonary veins and including the left atrial appendage, ^b^age in years; Standard deviation score (SDS) = [(X/M)L − 1]/(L*S), where X is the measured atrial volume in mL/m^2^ and L, M and S are the values interpolated for the child’s age; lower and upper limits correspond to a score of −2 and 2 and to the 3rd and 97th percentile, respectively

**Table 37:** Centiles of normal maximal left atrial volume (in mL) by BSA in boys^a^ using the biplane area length method^b^ according to reference [57].

BSA (m^2^)	5^th^	10^th^	25^th^	50^th^	75^th^	90^th^	95^th^
0.8	16	17	19	22	26	29	31
0.9	17	18	21	25	29	32	35
1.0	18	20	23	27	32	36	39
1.1	19	21	25	30	36	41	44
1.2	21	23	28	33	40	46	49
1.3	22	25	30	37	44	51	55
1.4	24	28	34	41	49	56	61
1.5	27	30	37	45	54	62	67
1.6	30	34	41	50	59	68	74
1.7	33	38	46	55	65	75	80
1.8	38	43	51	61	71	81	87
1.9	44	49	57	67	78	88	94
2.0	50	56	64	75	85	95	101

*Data are presented in centiles. BSA* body surface area

^a^Values for girls are not provided in the original publication, ^b^based on the two- and four-chamber-balanced steady-state free precession, excluding the left atrial appendage and pulmonary veins

**Table 38:** Normal maximal right atrial volume^a^ normalized to BSA in boys and girls; LMS parameters to calculate z-scores and centiles by age according to reference [56].

	Boys										Girls									
	LMS-parameters			Centiles (mL/m^2^)							LMS-parameters			Centiles (mL/m^2^)						
Age^b^	L	M	S	3^rd^	10^th^	25^th^	50^th^	75^th^	90^th^	97^th^	L	M	S	3^rd^	10^th^	25^th^	50^th^	75^th^	90^th^	97^th^
4	-	-	-	-	-	-	-	-	-	-	0.889	47.196	0.328	18	27	37	47	58	68	79
5	-	-	-	-	-	-	-	-	-	-	0.774	47.386	0.318	20	28	38	47	58	68	80
6	1.806	33.342	0.191	20	25	32	39	47	57	68	0.587	47.733	0.302	23	30	39	48	58	69	80
7	1.806	48.385	0.203	22	28	36	43	52	61	71	0.421	48.181	0.284	25	32	40	48	58	68	80
8	1.806	51.247	0.205	24	31	39	47	55	64	73	0.266	48.837	0.265	28	34	41	49	58	68	80
9	1.806	51.742	0.205	26	34	42	49	58	66	74	0.106	49.868	0.244	30	36	42	50	59	69	80
10	1.806	52.579	0.204	28	36	44	52	60	68	75	−0.033	51.098	0.221	33	38	44	51	59	69	80
11	1.806	54.891	0.200	30	39	47	54	62	69	76	−0.071	52.283	0.197	35	40	46	52	60	68	78
12	1.806	56.348	0.197	32	41	49	57	64	71	77	0.029	53.388	0.175	38	42	48	53	60	67	76
13	1.806	57.830	0.193	33	43	51	59	66	72	78	0.262	54.329	0.157	39	44	49	54	60	67	73
14	1.806	59.473	0.188	34	45	53	61	67	74	79	0.595	55.205	0.147	40	45	50	55	61	66	72
15	1.806	61.042	0.181	35	47	56	63	69	75	80	0.991	55.815	0.145	40	45	50	56	61	67	72
16	1.806	63.114	0.171	37	49	58	65	71	76	81	1.419	56.153	0.148	38	45	50	56	62	67	72
17	1.806	64.322	0.161	38	51	60	67	73	78	82	1.852	56.470	0.155	36	43	50	56	62	67	72
18	1.806	66.227	0.145	40	53	62	69	74	79	84	2.276	57.000	0.164	31	42	50	57	63	68	73
19	1.806	72.157	0.110	43	56	64	71	76	81	85	-	-	-	-	-	-	-	-	-	-
20	1.806	77.498	0.064	45	58	66	72	78	82	86	-	-	-	-	-	-	-	-	-	-

*Values represent LMS parameters and centiles. BSA* body surface area, *LMS* L = Lambda (skewness of the distribution), M = Mu (median), S = Sigma (variance)

^a^Quantified by a stack of axial cine balanced steady-state free precession images, excluding the superior and inferior vena cava and including the left atrial appendage, ^b^age in years; standard deviation score (SDS) =[(X/M)^L^ − 1]/(L*S), where X is the measured atrial volume in mL/m^2^ and L, M and S are the values interpolated for the child’s age; lower and upper limits correspond to a score of −2 and 2 and to the 3rd and 97th percentile, respectively

**Table 39:** Centiles of normal maximal right atrial volume (in mL) by BSA in boys using the mono-plane area-length method^a^ according to reference [57].

	Boys							Girls						
BSA (m^2^)	5^th^	10^th^	25^th^	50^th^	75^th^	90^th^	95^th^	5^th^	10^th^	25^th^	50^th^	75^th^	90^th^	95^th^
0.8	14	16	20	25	30	36	40	-	-	-	-	-	-	-
0.9	16	18	22	27	33	39	43	-	-	-	-	-	-	-
1.0	17	19	24	30	37	43	47	10	13	18	23	29	34	37
1.1	18	21	27	33	40	47	51	13	16	22	27	33	38	42
1.2	18	23	29	37	44	51	55	17	20	26	32	38	43	47
1.3	19	24	33	41	49	56	60	20	24	29	36	42	48	52
1.4	21	27	36	45	54	61	65	23	27	33	40	47	53	57
1.5	23	30	40	51	60	67	71	27	31	37	44	51	58	62
1.6	26	33	45	56	66	74	78	30	34	41	48	56	63	67
1.7	29	38	50	62	73	81	85	33	37	45	53	60	68	72
1.8	34	43	56	69	80	89	94	36	41	48	57	65	73	77
1.9	39	48	63	77	89	98	103	39	44	52	61	70	78	82
2.0	44	55	71	86	98	107	113	42	47	56	65	74	83	88

*Data are presented in centiles. BSA* body surface area

^a^Based on a four-chamber-balanced steady-state free precession view, excluding the right atrial appendage

**Age:** The left atrial conduit function was higher and the booster function lower than the reported adult values [54].

**Anthropometric measures:** BSA was highly associated with LA and RA volumes [54], [56], [57].

**Acquisition technique:** Breath-holding is preferred but may not be feasible for very young children. Using the bi-plane analysis approach (below) with steady state-free precession images, slice thickness measurements used for left ventricular analysis (5-8 mm) are also acceptable for atrial size analysis [50].

**Post-processing:** Atrial volumes are most frequently assessed using the biplane or monoplane area-length formulas adapted from echocardiography [58]. For the biplane method, left atrial volume is modeled as an ellipse and calculated as [8/3π]*[(A1*A2)/(L)] where A1 and A2 are the areas of the left atrium in the four-chamber and two-chamber view. L is the shortest length of either A1 or A2. In this method, note that the left atrial appendage and the confluence of the pulmonary veins are excluded from the atrial areas.

Atrial area can also be estimate from a single view, typically the four-chamber view by assuming A1 = A2. In this case, the monoplane area-length formula is 8(A)^2^/3πL, where A is the area of the atrium and L is the long-axis dimension of the atria in the same view. The majority of prior research recommends the biplane (ellipsoid) method.

Alternatively, a 3D approach using a stack of short-axis slices (Simpson’s rule, sum of the cross-sectional areas) can be used for measurement of atrial volume. This approach is theoretically the most accurate approach. However, additional short-axis acquisitions through the atria are required that might otherwise be omitted in a routine cardiac acquisition. Further, due to their small size, thinner slices would be more accurate than relatively thick 6-8 mm slices used to image the ventricles. Nacif et al. compared the biplane and Simpson’s method [46]. While both methods were found to be reproducible, the resulting volumes were not interchangeable.

## 9 Normal volumes, mass, and function of the left and right ventricle in athletes

### 9.1 Influencing factors

Given the limited cardiac MRI data, the discussion addressing the influence of gender, age, ethnicity, sport type, and volume on athlete heart mainly relies on echocardiographic studies.

**Table 40:** References. Normal volumes, mass, dimensions, and function of the left and right ventricle in athletes.

First author, year n, male:female Age range Study population	CMR acquisition	Parameter	Post-processing
Prakken, 2010 [59] 143:93 18-39 years Regular (9-18 h sports activity/week) and elite (>18 h sports activity/week) endurance athletes	• 1.5T, short-axis bSSFP	• LV, RV volumes and EF • LV mass end-diastolic	• Semiautomated contour-tracing software (View Forum cardiac package version R5.1V1L1 2006^a^). • LV and RV were traced from the apical short-axis slice to the basal slice of, respectively, the tricuspid valve and mitral valve. The papillary muscles and trabeculae were excluded from the endocardial contour and included in the blood volume.
Czimbalmos, 2019 [60] 101:49 (24.2 ± 4.8)^b^ years Elite athletes, >18 training h/wk (various types of highly dynamic and at least moderately static sports)	• 1.5T, short-axis bSSFP	• LV, RV volumes and EF • LV mass end-diastolic • Trabeculae and papillary muscles mass	• Quantification of LVEF, LVEDV, LVESV, and LV mass were performed using two different quantification methods: conventional quantification method (Medis QMass 7.6^c^) and threshold-based quantification method (Medis QMass 7.6 MassK algorithm^c^). • Trabeculae and papillary muscles were quantified using threshold-based quantification method. • For the analysis, the SI threshold was set to 50% invariably. • Using conventional quantification, the papillary muscles and trabeculae were excluded from the endocardial contour and included in the blood volume.
Starekova, 2020 [61] (ref. only) 42:0 18-36 years Competitive and elite athletes (22 soccer players, 19 triathletes)	• 1.5T, short- and long-axes bSSFP	• LV, RV volumes and EF • LV mass end-diastolic • Strain • Diastolic function	• Evaluation of ventricular volumes and LV mass was performed in standard fashion on short-axis cine images using cvi42 software.^d^ The papillary muscles and trabeculae were excluded from the endocardial contour and included in the blood volume. • Strain was analyzed using cine images and Segment feature tracking software version 2.1.R.6108.^e^ • Diastolic function was assessed with CMRtools software.^f^

*n* number of study subjects, *T* Tesla, *bSSFP* balanced steady-state free precession, *LV* left ventricular, *RV* right ventricular, *EF* ejection fraction, *EDV* end-diastolic volume, *ESV* end-systolic volume, *CMR* cardiovascular magnetic resonance

^a^Koninklijke Philips N.V., Amsterdam, The Netherlands; ^b^mean ± SD (age range not provided in original publication); ^c^Medis Medical Imaging Systems, Leiden, The Netherlands; ^d^Circle Cardiovascular Imaging Inc., Calgary, Alberta, Canada; ^e^Medviso, Lund, Sweden; ^f^Cardiovascular Imaging Solutions, London, UK

**Table 41:** Absolute and indexed (BSA) parameters of left ventricular volume, mass, and function in adult athletes for bSSFP imaging (papillary muscles/trabeculations included in left ventricular volume) according to reference [59].

	Regular athletes^a^ [mean ± SD (LL-UL)]		Elite athletes^b^ [mean ± SD (LL-UL)]	
Parameter	Men (n = 83)	Women (n = 60)	Men (n = 46)	Women (n = 33)
LVEDV (mL)	250 ± 32 (186-314)	194 ± 27 (140-248)	261 ± 39 (183-339)	199 ± 31 (137-261)
LVEDV/BSA (mL/m^2^)	123 ± 13 (97-149)	107 ± 14 (79-135)	129 ± 17 (95-163)	107 ± 14 (79-135)
LVESV (mL)	108 ± 20 (68-148)	86 ± 15 (56-116)	117 ± 24 (69-165)	85 ± 20 (45-125)
LVESV/BSA (mL/m^2^)	53 ± 9 (35-71)	48 ± 8 (32-64)	58 ± 11 (36-80)	46 ± 11 (24-68)
LVM (g)	125 ± 22 (81-169)	84 ± 17 (50-118)	139 ± 28 (83-195)	92 ± 15 (62-122)
LVM/BSA (g/m^2^)	62 ± 11 (40-84)	46 ± 9 (28-64)	69 ± 13 (43-95)	50 ± 8 (34-66)
LVEF (%)	57 ± 5 (47-67)	55 ± 4 (47-63)	55 ± 5 (45-65)	58 ± 7 (44-72)

*Data are means ± standard deviation. BSA* body surface area, *bSSFP* balanced steady-state free precession, *SD* standard deviation, *LL* lower limit, *UL* upper limit, *n* number of study subjects, *LV* left ventricular, *EDV* end-diastolic volume, *ESV* end-systolic volume, *M* mass, *EF* ejection fraction

^a^9-18 h sports activity/week, ^b^>18 h sports activity/week

**Table 42:** Indexed (BSA) parameters of left ventricular volume, mass, and function in adult athletes^a^ for bSSFP imaging (papillary muscles/trabeculations included in left ventricular mass) according to reference [60].

Parameter	Men (n = 101) [mean ± SD (LL-UL)]	Women (n = 49) [mean ± SD (LL-UL)]
LVEDV/BSA (mL/m^2^)	101 ± 12 (77-125)	89 ± 10 (69-109)
LVESV/BSA (mL/m^2^)	35 ± 7 (21-49)	30 ± 7 (16-44)
LVSV/BSA (mL/m^2^)	66 ± 8 (50-82)	59 ± 7 (45-73)
LVM/BSA (g/m^2^)	113 ± 17 (79-147)	84 ± 12 (60-108)
LVEF (%)	66 ± 5 (56-76)	66 ± 6 (54-78)

*Data are means ± standard deviation. BSA* body surface area, *bSSFP* balanced steady-state free precession, *n* number of study subjects, *SD* standard deviation, *LL* lower limit, *UL* upper limit, *LV* left ventricular, *EDV* end-diastolic volume, *ESV* end-systolic volume, *SV* stroke volume, *M* mass, *EF* ejection fraction

^a^>18 training hours/week

**Table 43:** Absolute and indexed (BSA) parameters of right ventricular volume, mass, and function in adult athletes for bSSFP imaging (papillary muscles/trabeculations included in right ventricular volume) according to reference [59].

	Regular athletes^a^ [mean ± SD (LL-UL)]		Elite athletes^b^ [mean ± SD (LL-UL)]	
Parameter	Men (n = 83)	Women (n = 60)	Men (n = 46)	Women (n = 33)
RVEDV (mL)	277 ± 36 (205-349)	209 ± 29 (151-267)	291 ± 48 (195-387)	219 ± 35 (149-289)
RVEDV/BSA (mL/m^2^)	136 ± 16 (104-168)	115 ± 15 (85-145)	144 ± 20 (104-184)	118 ± 17 (84-152)
RVESV (mL)	135 ± 25 (85-185)	102 ± 17 (68-136)	148 ± 30 (88-208)	103 ± 24 (55-151)
RVESV/BSA (mL/m^2^)	66 ± 12 (42-90)	57 ± 9 (39-75)	73 ± 13 (47-99)	56 ± 13 (30-82)
RVM (g)	29 ± 6 (17-41)	23 ± 4 (15-31)	30 ± 6 (18-42)	25 ± 5 (15-35)
RVM/BSA (g/m^2^)	14 ± 3 (8-20)	13 ± 2 (9-17)	15 ± 2 (11-19)	14 ± 3 (8-20)
RVEF (%)	51 ± 4 (43-59)	51 ± 4 (43-59)	50 ± 4 (42-58)	53 ± 7 (39-67)

*Data are means ± standard deviation. BSA* body surface area, *bSSFP* balanced steady-state free precession, *SD* standard deviation, *LL* lower limit, *UL* upper limit, *n* number of study subjects, *RV* right ventricular, *EDV* end-diastolic volume, *ESV* end-systolic volume, *M* mass, *EF* ejection fraction

^a^9-18 h sports activity/week, ^b^>18 h sports activity/week

**Gender:** Male athletes’ hearts are larger than female athletes’ hearts before and after indexing for body surface area. However, it is inconclusive whether sports activity leads to gender specific changes in volume and mass [59], [60], [62], [63].

**Age:** Cardiac adaptation to exercise is age dependent [63]. Simplified age stratification used in athlete screening differs between young athletes (<35 years) and master athletes (≥35 years) [64]. Generally, the effects of training on cardiac structure and function are most pronounced in young athletes [65], [66]. While both groups show increased ventricular size and mass compared to sedentary individuals, master athletes exhibit larger left ventricular mass and left atrial size, and less ventricular dilation compared to their younger counterparts [65], [66]. This likely reflects the combined effects of cardiovascular aging, training patterns, and cardiovascular risk factors [65], [66]. One additional group that should be considered are adolescent athletes (12-17 years), in whom the increase in LV cavity size and maximal wall thickness is rather modest compared to sedentary controls [63]. Finally, cumulative years of active training or changes in sporting activity may further influence the aging athlete’s heart.

**Anthropometric measures:** It is established that heart size is positively correlated with body size, hence all measures should be index on body surface area [62], [63], [64].

**Sport discipline and training volume:** Sport discipline and exercise volume have impact on cardiac remodeling and function [59], [60], [61], [64]. In general, higher levels of training volume were shown to be associated with more pronounced remodeling (increased ventricular volumes and LV mass) [59]. Dynamic exercise involves contraction or large muscle groups and maximal aerobic activity. Static exercise refers to exercise with sustained muscular contraction, often repetitive, with maximum voluntary contraction. Dynamic exercise sports (e.g., endurance running) result in cardiac chamber and ventricular mass increase with eccentric hypertrophy [62], [63], [64]. Ejection fraction may be low normal or borderline [62]. Static exercise sports (e.g., weightlifting) typically results in minimal to no LV dilatation, and sometimes a concentric pattern of hypertrophy [62], [64]. With modern cross-training exercise regimes, cardiac responses are likely to overlap. The intensity level and cumulative sport exposure may vary among elite, regular or hobby athletes.

**The amount of papillary muscle or trabeculae:** Increased trabeculation may occur in athletes and should not be misinterpreted as LV non-compaction cardiomyopathy [64], [67]. Inclusion of papillary muscles and trabeculae in mass measurements significantly impacts results, potentially causing up to a 20% variation in mass or volumes [59] in athletes.

**Ethnicity:** Currently, most data regarding athletes were gathered from Caucasian, male-dominated populations [62], [63], [68]. Athletes of Black origin tend to have greater LV mass compared to other ethnicities. Greater trabeculation is reported to be more frequent in athletes of Black origin [62], [68] in addition to higher prevalence of concentric LV remodeling compared to White athletes. Mixed-race athletes exhibit a similar trend in LV wall thickening, but to a lesser extent than Black athletes [62], [68]. Further research is needed to understand how individual factors like geographical origin and mixed heritage influence sport-related cardiac adaptations.

## 10 Normal thickness of the compact left ventricular myocardium in adults

### 10.1 Influencing factors

**Gender:** The thickness of the compact left-ventricular myocardium is greater in men compared to women [10], [11], [19], [24], [69], [70].

**Table 44:** References. Normal thickness of the compact left ventricular myocardium in adults.

First author, year n, male:female Age range Study population	CMR acquisition	Parameter	Post-processing
Dawson, 2011 [69] 60:60 20-80 years Volunteers, UK	• 1.5T, short-axis bSSFP	• Wall thickness per segment for 16 segments (except segment 17). • Diastole and systole	• Measurements were obtained manually on 3 short-axis slices at the basal (2 cm down from the most basal short axis slice on the ventricular side of the atrioventricular junction), apical (2 cm up from the most apical slice showing LV myocardium) and midventricular (equidistant between basal and apical slice) level.
Kawel, 2012 [70] 131:169 54-91 years MESA, USA	• 1.5T, short- and long-axis (2- and 4ch) bSSFP	• Wall thickness per segment for 16 segments (except segment 17) on short-axis images and for 12 segments on long-axis images. • Diastole	• Measurements were obtained semi-automatically using QMass software version 7.2.^a^ • For measurements on long-axis images, endocardial and epicardial borders were contoured manually. Myocardial thickness was averaged over 33-34 measurements per segment that were automatically acquired by the software using the 2-dimensional centerline method. Minimum and maximum thickness per segment are also provided. • For measurements on short-axis images, endocardial and epicardial borders were contoured manually for all short-axis slices, where the myocardium was present as a complete circle. Each slice was divided into 6 sections and thickness was calculated as average of 16-17 measurements per section by means of the centerline method. Measurements were calculated for 16 segments. Minimum and maximum thickness per segment are also provided. • The contour of the epicardial surface was placed on the center of the “black line” caused by out-of-phase imaging on bSSFP images. • In cases of slightly blurred myocardial borders due to motion and limited spatial resolution, the contours were placed on the center of the blurred border region. • Papillary muscles and trabeculation were excluded from the compact myocardium.
Le Ven, 2015 [11] 196:238 18-36 years Volunteers, Canada	• 1.5T, short-axis bSSFP	• Wall thickness per segment for 16 segments (except segment 17) • Diastole	• Measurements were obtained semi-automatically using cvi42 software version 3.4.1.^b^ • LV endocardial and epicardial borders were traced semi-automatically with manual correction. Segmental wall thickness was quantified by the centerline method and averaged over 20-30 cords per segment.
Yeon, 2015 [10] 340:512 (61 ± 9) years^c^ FHS Offspring cohort, USA	• 1.5T, short-axis bSSFP	• Wall thickness of 2 segments (basal inferolateral and anteroseptal) • Diastole	• Single measurement obtained on the slice “immediately basal to the tips of the papillary muscles” for each of the two segments.
Meloni, 2021 [18] (ref. only) 50:50 20-70 years Caucasian volunteers, Italy	• 1.5T, short-axis bSSFP	• Wall thickness per segment for 16 segments (except segment 17) • Diastole	• Measurements were obtained manually at the thickest region of each segment, excluding papillary muscles and trabeculations. • Publication not included in calculation of weighted mean values since maximum wall thickness was measured compared to average wall thickness in the other publications.
Zhang, 2021 [19] 323:227 21-70 years Screening cohort, China	• 1.5T, short-axis bSSFP	• Wall thickness per segment for 16 segments (except segment 17) • Wall thickness calculated per level (basal, midventricular, apical) and global (average of all 16 segments).	• Measurements were obtained with a semi-automatic threshold-based segmentation software (cvi42 version 5.6.2^b^). • LV endocardial borders were traced by semi-automatic segmentation with manual correction. LV endocardial borders were traced manually.

*n* number of study subjects, *bSSFP* balanced steady-state free precession, *2ch* two-chamber view, 4ch four-chamber view, *MESA* Multi-Ethnic Study in Atherosclerosis, *FHS* Framingham Heart Study

^a^Medis Medical Imaging Systems, Leiden, The Netherlands; ^b^Circle Cardiovascular Imaging Inc., Calgary, Alberta, Canada; ^c^age range not provided in original publication

**Table 45:** Normal left ventricular myocardial thickness (in mm) in men and women measured on short-axis bSSFP at diastole.

			Men			Women		
Level	Segment	References	n	Mean ± SD	LL-UL	n	Mean ± SD	LL-UL
Basal	1	[11], [19], [69], [70]	710	8.1 ± 1.5	5-11	694	6.6 ± 1.3	4-9
	2	[10], [11], [19], [69], [70]	1050	9.0 ± 1.6	6-12	1206	7.4 ± 1.4	5-10
	3	[11], [19], [69], [70]	710	8.9 ± 1.3	6-11	694	7.3 ± 1.1	5-9
	4	[11], [19], [69], [70]	710	8.1 ± 1.3	6-11	694	6.6 ± 1.1	4-9
	5	[10], [11], [19], [69], [70]	1050	7.8 ± 1.4	5-11	1206	6.3 ± 1.2	4-9
	6	[11], [19], [69], [70]	710	7.8 ± 1.4	5-11	694	6.4 ± 1.1	4-9
Mid-cavity	7	[11], [19], [69], [70]	710	7.0 ± 1.4	4-10	694	5.8 ± 1.2	4-8
	8	[11], [19], [69], [70]	710	7.5 ± 1.4	5-10	694	6.2 ± 1.2	4-8
	9	[11], [19], [69], [70]	710	8.0 ± 1.3	6-11	694	6.7 ± 1.1	4-9
	10	[11], [19], [69], [70]	710	7.3 ± 1.5	4-10	694	6.1 ± 1.2	4-8
	11	[11], [19], [69], [70]	710	6.8 ± 1.5	4-10	694	5.6 ± 1.3	3-8
	12	[11], [19], [69], [70]	710	6.9 ± 1.5	4-10	694	5.8 ± 1.2	3-8
Apical	13	[11], [19], [69], [70]	710	6.7 ± 1.4	4-9	694	6.0 ± 2.0	2-10
	14	[11], [19], [69], [70]	710	6.9 ± 1.4	4-10	694	5.8 ± 1.2	3-8
	15	[11], [19], [69], [70]	710	6.3 ± 1.3	4-9	694	5.3 ± 1.1	3-7
	16	[11], [19], [69], [70]	710	6.5 ± 1.4	4-9	694	5.7 ± 1.1	4-8

*Data are means ± standard deviation. bSSFP* balanced steady-state free precession, *n* number of study subjects included in the weighted mean values, *LL* lower limit, *UL* upper limit, segments: *1* basal anterior, *2* basal anteroseptal, *3* basal inferoseptal, *4* basal inferior, *5* basal inferolateral, *6* basal anterolateral, *7* mid anterior, *8* mid anteroseptal, *9* mid inferoseptal, *10* mid inferior, *11* mid inferolateral, *12* mid, anterolateral, *13* apical anterior, *14* apical septal, *15* apical inferior, *16* apical lateral

**Table 46:** Normal left ventricular myocardial thickness (in mm) in men and women measured on long-axis bSSFP images according to reference [70].

		Men (n = 131)		Women (n = 169)	
Level	Region	Mean ± SD	LL-UL	Mean ± SD	LL-UL
Basal	Anterior	8.2 ± 1.3	6-11	7.0 ± 1.1	5-9
	Inferior	8.2 ± 1.3	6-11	6.7 ± 1.1	5-9
	Septal	9.1 ± 1.3	7-12	7.3 ± 1.1	5-10
	Lateral	7.6 ± 1.3	5-10	6.0 ± 1.1	4-8
	*Mean*	8.3 ± 1.0	6-10	6.8 ± 0.9	5-9
Mid-cavity	Anterior	6.0 ± 1.3	3-9	4.9 ± 1.1	3-7
	Inferior	7.7 ± 1.3	5-10	6.5 ± 1.1	4-9
	Septal	8.3 ± 1.3	6-11	6.8 ± 1.1	5-9
	Lateral	6.6 ± 1.3	4-9	5.3 ± 1.1	3-8
	*Mean*	7.2 ± 1.0	5-9	5.9 ± 0.9	4-8
Apical	Anterior	5.1 ± 1.3	3-8	4.2 ± 1.1	2-6
	Inferior	5.8 ± 1.3	3-8	5.0 ± 1.1	3-7
	Septal	5.8 ± 1.3	3-8	5.0 ± 1.1	3-7
	Lateral	5.5 ± 1.3	3-8	4.6 ± 1.1	2-7
	*Mean*	5.6 ± 1.0	4-8	4.7 ± 0.9	3-7

*Data are means ± standard deviation. bSSFP* balanced steady-state free precession, *n* number of study subjects, *SD* standard deviation, *LL* lower limit, *UL* upper limit

**Age:** Two studies with sample sizes of around 300 subjects revealed no relationship of left-ventricular myocardial thickness with age [24], [70]. Dawson et al. noted a smaller myocardial thickness for each segment in the fourth compared to the third decade and progressively greater myocardial thickness between each of the following age deciles in 120 subjects (10 subjects per decile and gender) [69]. Zhang et al. also found a greater global myocardial thickness in older compared to younger subjects of 550 healthy Chinese aged 21-70 years [19].

**Ethnicity:** In a subset of 300 healthy individuals of the MESA study population, multivariable analysis revealed a higher left ventricular myocardial thickness in Blacks compared to Whites and a lower thickness in participants of Hispanic ethnicity compared to Whites [70].

**Left ventricular volumes and function:** In MESA greater myocardial thickness was associated with a lower left-ventricular end-diastolic, end-systolic, and stroke volume in adjusted models, while there was no relationship with ejection fraction [70].

**Post-processing:** There is potential variation of left ventricular myocardial thickness with different contouring technique, measurement methods (single manual measurement versus semi-automatic quantification) and with different software algorithms.

## 11 Normal thickness of the compact left ventricular myocardium in children

### 11.1 Influencing factors

**Gender:** In linear regression analysis, male gender correlated with higher left-ventricular myocardial thickness [52].

**Table 47:** References. Normal thickness of the compact left ventricular myocardium in children.

First author, year n, male:female Age range Study population	CMR acquisition	Parameter	Post-processing
Krupickova, 2021 [52] 97:64 6-18 years Children with scans for clinical reasons but without cardiovascular disease and normal CMR, UK and Germany	• 1.5T, short-axis bSSFP	• Diastolic wall thickness per level (basal, midventricular, apical).	• Measurements were obtained on three short-axis slices at the basal, midventricular, and apical level. • The average of three measurements obtained per segment for each of the 16 segments was calculated. • The average myocardial thickness of segments 1-6 was calculated for the basal level, the average of segments 7-12 for the midventricular level, and the average of segments 13-16 for the apical level. • Since BSA was a strong determinant for left-ventricular myocardial thickness according to regression analysis, centiles were calculated by BSA.

*n* number of study subjects, *T* Tesla, *bSSFP* balanced steady-state free precession

**Table 48:** Centiles (in mm) of normal left ventricular myocardial thickness by BSA in boys for short-axis bSSFP at diastole according to reference [52].

	Basal level							Midventricular level							Apical level						
BSA (m^2^)	5^th^	10^th^	25^th^	50^th^	75^th^	90^th^	95^th^	5^th^	10^th^	25^th^	50^th^	75^th^	90^th^	95^th^	5^th^	10^th^	25^th^	50^th^	75^th^	90^th^	95^th^
0.8	2.4	2.6	3.0	3.4	3.9	4.3	4.5	2.3	2.5	2.9	3.4	3.8	4.3	4.5	2.4	2.4	2.6	2.8	3.0	3.2	3.4
0.9	2.6	2.9	3.2	3.7	4.1	4.5	4.7	2.5	2.7	3.2	3.6	4.1	4.5	4.7	2.4	2.5	2.6	2.8	3.1	3.4	3.6
1.0	2.9	3.1	3.5	3.9	4.4	4.7	5.0	2.7	2.9	3.4	3.8	4.3	4.7	5.0	2.4	2.5	2.7	2.9	3.2	3.6	3.9
1.1	3.1	3.4	3.8	4.2	4.6	5.0	5.2	2.9	3.1	3.6	4.1	4.5	5.0	5.2	2.4	2.5	2.7	3.0	3.4	3.8	4.2
1.2	3.4	3.6	4.0	4.4	4.8	5.2	5.4	3.1	3.4	3.8	4.3	4.8	5.2	5.5	2.4	2.5	2.8	3.2	3.6	4.1	4.4
1.3	3.7	3.9	4.3	4.7	5.1	5.4	5.7	3.3	3.6	4.0	4.5	5.0	5.4	5.7	2.5	2.6	2.9	3.3	3.8	4.3	4.7
1.4	3.9	4.1	4.5	4.9	5.3	5.7	5.9	3.5	3.8	4.2	4.7	5.2	5.7	5.9	2.6	2.7	3.1	3.5	4.0	4.5	4.8
1.5	4.2	4.4	4.8	5.2	5.6	5.9	6.1	3.8	4.0	4.5	5.0	5.4	5.9	6.1	2.7	2.9	3.3	3.7	4.2	4.7	5.0
1.6	4.4	4.7	5.0	5.4	5.8	6.2	6.4	4.0	4.3	4.7	5.2	5.7	6.1	6.3	2.9	3.2	3.5	4.0	4.4	4.9	5.1
1.7	4.7	4.9	5.3	5.7	6.0	6.4	6.6	4.3	4.5	4.9	5.4	5.9	6.3	6.5	3.2	3.4	3.8	4.2	4.6	5.0	5.3
1.8	5.0	5.2	5.5	5.9	6.3	6.6	6.8	4.5	4.8	5.2	5.6	6.1	6.5	6.7	3.4	3.6	4.0	4.4	4.8	5.2	5.4
1.9	5.2	5.4	5.8	6.2	6.5	6.9	7.1	4.8	5.1	5.4	5.9	6.3	6.7	6.9	3.5	3.8	4.1	4.5	4.9	5.2	5.4
2.0	5.5	5.7	6.0	6.4	6.8	7.1	7.3	5.1	5.3	5.7	6.1	6.5	6.8	7.0	3.6	3.8	4.2	4.5	4.9	5.2	5.3
2.1	5.7	5.9	6.3	6.6	7.0	7.3	7.5	5.4	5.6	5.9	6.3	6.7	7.0	7.2	3.6	3.8	4.1	4.4	4.7	5.0	5.1
2.2	6.0	6.2	6.5	6.9	7.3	7.6	7.8	5.7	5.9	6.2	6.5	6.9	7.2	7.4	3.6	3.8	4.0	4.3	4.6	4.8	4.9

*Data are presented in centiles. BSA* body surface area, *bSSFP* balanced steady-state free precession

**Table 49:** Centiles (in mm) of normal left ventricular myocardial thickness by BSA in girls for short-axis bSSFP at diastole according to reference [52].

	Basal level							Midventricular level							Apical level						
BSA (m^2^)	5^th^	10^th^	25^th^	50^th^	75^th^	90^th^	95^th^	5^th^	10^th^	25^th^	50^th^	75^th^	90^th^	95^th^	5^th^	10^th^	25^th^	50^th^	75^th^	90^th^	95^th^
0.8	2.1	2.3	2.8	3.3	3.9	4.3	4.6	3.3	3.4	3.5	3.6	3.7	3.9	4.0	1.9	2.1	2.4	2.6	2.9	3.2	3.4
0.9	2.3	2.6	3.0	3.5	4.0	4.4	4.7	3.3	3.4	3.5	3.6	3.8	4.0	4.1	2.0	2.2	2.4	2.7	3.0	3.3	3.5
1.0	2.5	2.8	3.2	3.6	4.1	4.5	4.7	3.3	3.4	3.5	3.7	3.9	4.1	4.3	2.1	2.3	2.5	2.8	3.1	3.4	3.6
1.1	2.8	3.0	3.4	3.8	4.2	4.6	4.8	3.3	3.4	3.5	3.7	4.0	4.3	4.5	2.2	2.3	2.6	2.9	3.2	3.5	3.7
1.2	3.0	3.2	3.6	4.0	4.4	4.7	4.9	3.3	3.4	3.5	3.8	4.1	4.4	4.7	2.3	2.4	2.7	3.0	3.3	3.6	3.8
1.3	3.3	3.5	3.8	4.2	4.5	4.8	5.0	3.3	3.4	3.6	3.9	4.3	4.7	4.9	2.4	2.6	2.8	3.2	3.5	3.7	3.9
1.4	3.6	3.8	4.1	4.4	4.7	5.0	5.2	3.3	3.5	3.7	4.1	4.5	5.0	5.3	2.5	2.7	3.0	3.3	3.6	3.9	4.1
1.5	3.9	4.1	4.3	4.7	5.0	5.2	5.4	3.4	3.6	3.9	4.3	4.8	5.3	5.6	2.7	2.8	3.1	3.5	3.8	4.1	4.2
1.6	4.2	4.4	4.6	4.9	5.2	5.5	5.6	3.5	3.7	4.2	4.6	5.1	5.6	5.9	2.8	3.0	3.3	3.6	4.0	4.3	4.4
1.7	4.6	4.7	4.9	5.2	5.5	5.7	5.9	3.7	4.0	4.5	5.0	5.5	5.9	6.2	3.0	3.2	3.5	3.8	4.2	4.5	4.7
1.8	4.9	5.0	5.3	5.5	5.8	6.0	6.1	4.0	4.3	4.8	5.3	5.8	6.2	6.4	3.2	3.4	3.7	4.1	4.4	4.7	4.9
1.9	5.3	5.4	5.6	5.8	6.1	6.3	6.4	4.4	4.7	5.2	5.7	6.1	6.4	6.6	3.5	3.6	3.9	4.3	4.6	4.9	5.1
2.0	5.7	5.8	6.0	6.2	6.4	6.6	6.7	5.0	5.3	5.7	6.1	6.5	6.8	6.9	3.7	3.9	4.2	4.5	4.9	5.2	5.4
2.1	6.1	6.2	6.4	6.6	6.8	6.9	7.1	5.6	5.8	6.2	6.6	6.9	7.2	7.3	3.9	4.1	4.4	4.8	5.1	5.4	5.6
2.2	6.5	6.6	6.8	7.0	7.1	7.3	7.4	6.3	6.5	6.8	7.1	7.4	7.6	7.8	4.1	4.3	4.6	5.0	5.3	5.7	5.9

*Data are presented in centiles. BSA* body surface area, *bSSFP* balanced steady-state free precession

**Age:** In multivariable models, age was a predictor of left-ventricular myocardial thickness only at the midventricular level in girls [52].

**Anthropometric measures:** In the study by Krupickova et al., body surface area was the strongest predictor of left-ventricular myocardial thickness at the basal and midventricular level [52]. Regarding myocardial thickness at the apical level body weight was a strong predictor in girls and body height in boys.

**Post-processing:** As for the adult, there is potential variation of left ventricular myocardial thickness with different contouring techniques, measurement methods (single manual measurement versus semi-automatic quantification), and with different software algorithms.

## 12 Normal values of left ventricular trabeculation in adults

### 12.1 Influencing factors

**Gender:** Dawson et al. did not find a difference in the thickness of left-ventricular trabeculation between 60 healthy men and 60 healthy women [69]. Other studies found a statistically significant higher left-ventricular trabeculation mass in males compared to females [13], [17]. Dawson et al. and Kawel et al. demonstrated no sex difference for (maximal) trabeculated/compact left ventricular wall thickness ratio [69], [71], while Tizon et al. found a higher ratio in women compared to men [73]. The ratio of trabeculated/compact myocardial mass did not show a gender difference [13]. In one study, trabecular complexity of the left ventricle quantified by the global fractal dimension was greater in men compared to women [77]. However, Captur et al. demonstrated that the positive association between maximal apical fraction dimension and male gender did not persist after adjustment for other influencing factors [76].

**Fig. 6:**
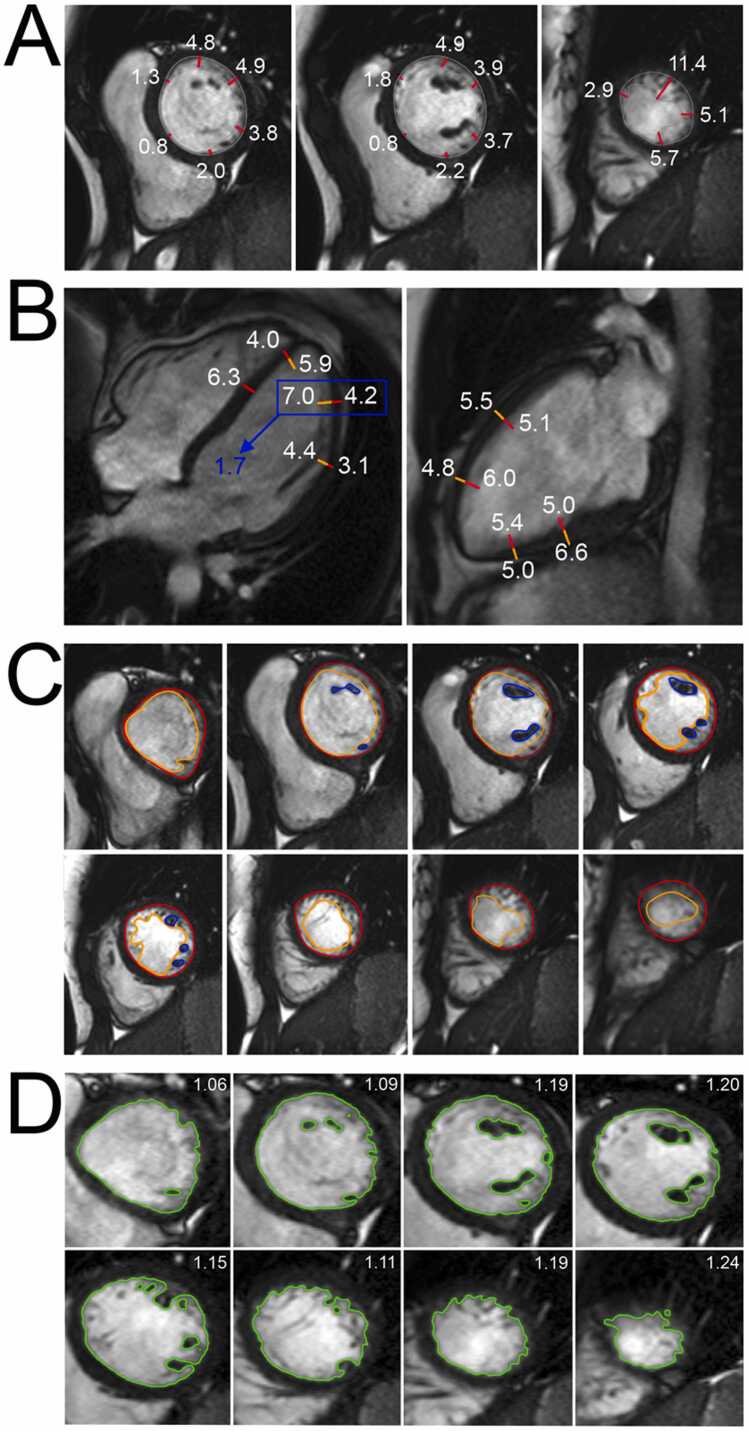
Example of measurement approaches for left ventricular trabeculation. (A) End-diastolic thickness (in mm) of trabeculation according to the methodology in [69]: three slices representing base, mid, and apex were selected from within the entire left ventricular stack; trabeculated myocardial thickness was measured per slice; segment 17 excluded from analysis; authors do not clarify whether papillary muscles had been included or excluded from the trabecular measurement—in this reproduction we have excluded papillary muscles. (B) Maximal non-compacted (NC, red lines)/compacted (C, orange lines) wall thickness ratio according to the methodology in [71]: papillary muscles that were clearly observed as compact tubular structures were not included in the measurements; measurements in mm are shown in white and the maximal NC/C parameter highlighted in blue. (C) Trabeculation mass according to the methodology in [13]: the endocardial contour (red) was manually drawn; the trabecular contour (orange) was automatically segmented and papillary muscles (blue) that were included in the compact myocardial mass, were semi-automatically segmented; all slices of the left ventricular short-axis stack were analyzed. (D) Fractal dimension according to the methodology in [80]: using a semi-automatic level-set segmentation with bias field correction; all slices of the left ventricular short-axis stack are analyzed except for the apical slice; fractal dimensions per slice reported in the top right corner

**Table 50:** References. Normal values of left ventricular trabeculation in adults.

First author, year n, male:female Age range Study population	CMR acquisition	Parameter	Post-processing (Fig. 6)
Dawson, 2011 [69] 60:60 20-80 years Volunteers, UK	• 1.5T, short-axis bSSFP • Diastole and systole	• Trabeculation thickness • Trabeculation/compact LV myocardial thickness ratio	• Measurements obtained on 3 short-axis slices at the basal (2 cm down from the most basal short axis sliceon the ventricular side of the atrioventricular junction), apical (2 cm up from the most apical slice showing LV myocardium), and midventricular (equidistant between basal and apical slice) level. • Trabeculation thickness measured manually per segment for 16 segments (segment 17, apex excluded) at the “peak of the most prominent trabeculae in each segment.”
Kawel, 2012 [71]^a^ 175:192 54-94 years MESA, USA	• 1.5T, long-axis bSSFP • Diastole	• Trabeculation/compact LV myocardial thickness ratio	• Thickness of trabeculated and compact myocardium measured perpendicular to the compact myocardium on horizontal and vertical long-axis images in the middle of each of 8 segments (7, 9, 10, 12, 13, 14, 15, 16). Maximum ratio of all 8 segments determined. • 50% of the chemical shift artifact (“black line”) on the epicardial surface was included in the compact myocardium. • Papillary muscles were excluded from trabeculated and compact myocardium.
Captur, 2013 [72] 40^b^ (total; trabeculation mass and ratio) 51:54 (FD) 18-85 years Black and White volunteers, UK	• 1.5T, long- and short-axis bSSFP • Diastole	• Trabeculation/compact LV myocardial thickness ratio • Trabeculation/compact LV myocardial mass ratio • Fractal dimension	*Thickness ratio* • Was calculated for a single segment with the maximal thickness of the trabeculation of all 16 segments (segment 17 excluded) on long-axis images. *Mass ratio* • Semi-automatic tracing of the epicardial and the endocardial border of the compact LV myocardium on short-axis images to determine the mass of the LV compact myocardium. Tracing of the epicardial and the trabeculation border to determine global LV mass (compact + trabeculated myocardium). • Trabeculation mass calculated as the difference between the global LV mass and the mass of the compact left ventricular myocardium (“Jacquier method”). • Blood between trabeculae was included in trabeculation mass. Papillary muscles were included in the mass of the compact LV myocardium. *Fractal dimension* • Automatic thresholding, binarization, edge-detection, and fractal analysis by means of a box-counting method on short-axis images using in-house software. • Global LV FD was calculated by averaging the FD from each short-axis slice. Maximal FD at the basal, midventricular, and apical level were determined. • Papillary muscles included in the endocardial complexity.
Tizón-Marcos, 2014 [73] 44:56 18-35 years Volunteers, Canada	• 1.5T, long- and short-axis bSSFP • Diastole and systole	• Trabeculation/compact LV myocardial thickness ratio • Trabeculation/total LV myocardial thickness ratio • Percent trabeculation/total myocardial area	• Measurements obtained on 3 short-axis slices (basal, mid, and apical left ventricle) and on horizontal and vertical long -xis -axis images. • After delineation of the epicardial border and the trabeculation border, the total thickness of the trabeculated and the compact myocardium was measured semi-automatically by the centerline method along 20-30 chords/segment. Then the inner border of the compact myocardium was delineated to calculate the thickness and area of the compact myocardium for each segment by subtracting the thickness of the trabeculated myocardium from the full thickness of the trabeculated and the compact myocardium. • Papillary muscles were excluded from trabeculated and compact myocardium.
Amzulescu, 2015 [74] 22:26 (60 ± 10) years^c^ Volunteers, Belgium	• 1.5T and 3T, long- and short-axis bSSFP • Diastole	• Trabeculation/compact LV myocardial thickness ratio • Trabeculation/compact LV myocardial mass ratio	*Trabeculation thickness ratio* • Calculated for a single segment with the maximal thickness of the trabeculation of all 16 segments (segment 17 excluded) measured on long-axis images. • Papillary muscles were excluded from trabeculated and compact myocardium. *Trabeculation mass ratio* • Semi-automatic tracing of the epicardial and the endocardial border of the compact LV myocardium on short-axis images to determine the mass of the LV myocardium. Tracing of the epicardial and the trabeculation border to determine global LV mass (compact + trabeculated myocardium). • Trabeculation mass calculated as the difference between the global LV mass and the mass of the compact LV myocardium (“Jacquier method”). • Papillary muscles were excluded from trabeculation and compact myocardial mass. • Blood between trabeculae was included in trabeculation mass.
André, 2015 [75] 58:59 20->50 years Volunteers, Germany	• 1.5T, long- and short-axis bSSFP • Diastole	• Trabeculation/compact LV myocardial thickness ratio • Trabeculation volume indexed	*Trabeculation thickness ratio* • Maximal NC/C thickness ratio of 16 segments measured on long-axis images. • Papillary muscles were excluded from trabeculated and compact myocardium. *Trabeculation volume* • Volume between the endocardial border and a line between the trabeculation-free left ventricular cavity and the ventricular volume containing trabeculation on short-axis images. • Blood between rabeculae was included, papillary muscles were excluded from trabeculation volume. • Trabeculation volume indexed to BSA.
Captur 2015 [76] 279:325 46-91 years MESA, USA	• 1.5T, short-axis bSSFP • Diastole	• Fractal dimension	• Automatic thresholding, binarization, edge-detection, and fractal analysis by means of a box-counting method on short-axis images using in-house software. • Papillary muscles included in the endocardial complexity. • Most apical slice of the LV short-axis stack was excluded. • Maximal apical FD was derived from the apical half of the LV short-axis stack (discounting the middle slice in unevenly numbered stacks).
Cai, 2017 [77] 91:89 20-69 years Volunteers, Singapore	• 3T, short-axis bSSFP • Diastole	• Fractal dimension	• Automatic thresholding, binarization, edge-detection, and fractal analysis by means of a box-counting method on short-axis images using in-house software. • Papillary muscles included in the endocardial complexity. • Most apical slice of the LV short-axis stack was excluded. • Global LV FD was calculated by averaging the FD from each short-axis slice. Maximal apical FD was derived from the apical half of the LV short-axis stack (discounting the middle slice in unevenly numbered stacks).
Bentatou, 2018 [13] 70:70 20-69 years Volunteers, France	• 1.5T, short-axis bSSFP • Diastole	• Trabeculation mass indexed • Trabeculation mass indexed/compact LV myocardial mass indexed ratio	• Semiautomatic contouring of the border of trabeculations and the endocardial and epicardial border of the compact myocardium to determine trabeculation mass (between trabeculation and endocardial contour) and compact left ventricular myocardial mass (between endocardial and epicardial contour) (“Bricq method”). • Papillary muscles and blood between trabeculae were excluded from trabeculation mass. Papillary muscles were included in the mass of the compact left ventricular myocardium. • Trabeculation mass and compact myocardial mass indexed to body surface area. Ratio expressed as percentage.
Gregor, 2021 [17] 100:100 20->50 Volunteers, Hungary	• 1.5T, short-axis bSSFP • Diastole	• Trabeculation mass indexed • Trabeculation mass indexed/compact LV myocardial mass indexed ratio • Trabeculation mass indexed/end-diastolic volumen indexed ratio	• Semiautomatic threshold based technique used to differentiate between blood and myocardium. Compact left ventricular myocardium between endo- and epicardial contour. Trabeculation mass calculated as voxels below a chosen threshold within the epicardial contour. • Papillary muscles included in trabeculation mass. Blood between trabeculae excluded from trabeculation mass. • Trabeculation mass and compact myocardial mass indexed to body surface area.
Wang, 2021 [78] 45:55 36-59 years Volunteers, China	• 3T, short-axis bSSFP • Diastole	• Fractal dimension	*Fractal dimension* • After manual delineation of the endocardial and epicardial contour, a Matlab-based software (MathWorks, Natick, Massachusetts, USA) calculated the FD. • Papillary muscles included in the endocardial complexity. • Global FD was defined as the average FD of all slices. Maximum and mean FD were calculated for the basal and apical halves.
Aung, 2024 [79] 2655:4729 45-80 years UK Biobank, UK	• 1.5T, short-axis bSSFP • Diastole	• Trabeculation mass • Trabeculation mass/BSA • Trabeculation mass/total myocardial mass	*Trabeculation mass* • Automatic segmentation software based on a deep-learning model. • Blood between trabeculae and papillary muscles was excluded from trabeculation mass. • Total myocardial mass calculated as the sum of the compact myocardial mass, the trabeculation mass, and the papillary muscle mass.

*n* number of study subjects, *bSSFP* balanced steady-state free precession, *LV* left ventricle, *MESA* Multi-Ethnic Study in Atherosclerosis, *FD* fractal dimension

^a^Additional calculations performed for this review, ^b^male:female ratio not provided in original publication, ^c^age range not provided in original publication

**Table 51:** Normal values for trabeculation thickness and trabeculation/compact myocardial thickness ratio in adults measured on short- and long-axis bSSFP images at diastole.

Segment	Trabeculation thickness (mm) on SAX (mean ± SD or median, IQR)^a^ according to ref. [69] (n = 120)	Trabeculation/compact myocardial thickness ratio on SAX (mean ± SD) according to ref. [69] (n = 120)	Trabeculation/compact myocardial thickness ratio on SAX (mean ± SD) according to ref. [73] (n = 100)	Trabeculation/compact myocardial thickness ratio on LA (mean ± SD) according to ref. [73] (n = 100)
1	3.0 (0, 4.6)	0.38 ± 0.04	1.60 ± 1.30	0.50 ± 0.38
2	0	0	0.84 ± 0.86	0.36 ± 0.21
3	0	0	0.37 ± 0.33	0.36 ± 0.21
4	0	0.06 ± 0.02	0.80 ± 0.85	0.52 ± 0.32
5	0 (0, 3.9)	0.32 ± 0.04	1.15 ± 1.10	0.36 ± 0.25
6	0 (0, 4.1)	0.29 ± 0.03	1.17 ± 0.88	0.36 ± 0.25
7	5.6 ± 2.8	0.88 ± 0.04	2.21 ± 1.65	0.75 ± 0.73
8	0	0.08 ± 0.03	1.48 ± 1.48	0.37 ± 0.25
9	0	0	0.59 ± 0.99	0.37 ± 0.25
10	0 (0, 2.1)	0.20 ± 0.04	1.03 ± 1.00	0.82 ± 0.56
11	4.2 ± 2.5	0.70 ± 0.04	1.60 ± 1.57	0.35 ± 0.22
12	4.4 ± 2.7	0.76 ± 0.05	1.51 ± 1.78	0.35 ± 0.22
13	5.6 ± 2.7	0.91 ± 0.05	2.23 ± 1.84	0.91 ± 0.56
14	0	0.11 ± 0.03	1.45 ± 1.45	0.84 ± 0.38
15	0 (0, 4.5)	0.40 ± 0.05	1.79 ± 1.56	0.97 ± 0.72
16	7.1 ± 2.4	1.19 ± 0.04	2.02 ± 1.36	0.95 ± 0.71

*Data are presented as means ± standard deviation or median and interquartile range, as indicated. bSSFP* balanced steady-state free precession, *n* number of study subjects, *SAX* short axis, *LA* long axis (two- and four-chamber view), *IQR* interquartile range, *SD* standard deviation, segments: *1* basal anterior, *2* basal anteroseptal, *3* basal inferoseptal, *4* basal inferior, *5* basal inferolateral, *6* basal anterolateral, *7* mid anterior, *8* mid anteroseptal, *9* mid inferoseptal, *10* mid inferior, *11* mid inferolateral, *12* mid anterolateral, *13* apical anterior, *14* apical septal, *15* apical inferior, *16* apical lateral.

^a^Depending on data distribution

**Table 52:** Maximal trabeculation/compact myocardial thickness ratio in adults measured on long axis bSSFP at diastole.

Reference	n		
[71]	323	5th, 50th, 95th percentile	1.0, 2.2, 4.6
[72]	20 White 20 Black	Mean ± SD	1.4 ± 0.4 (White) 1.4 ± 0.5 (Black)
[74]	48	Median (IQR)	1.5 (1.1-2.0)
[75]	58 men 59 women	Mean ± SD	2.2 ± 0.7 (men) 2.5 ± 0.9 (women)

*Data are presented as percentiles (5th, 50th, 95th), means ± standard deviation or median and interquartile range, as indicated. bSSFP* balanced steady-state free precession, *n* number of study subjects, *SD* standard deviation, *IQR* interquartile range

**Table 53:** Normal values for mass and volume of trabeculated left ventricular myocardium in adults measured on short-axis bSSFP images.

Parameter	Post-processing	Men		Women	
		n	Mean ± SD	n	Mean ± SD
Trabeculation mass (mass of the trabeculated LV myocardium) per BSA (g/m^2^) [13]	Papillary muscles and blood between trabeculae excluded	70	5.4 ± 2.3	70	4.0 ± 2.3
Trabeculation volume (volume of the trabeculated LV myocardium) per BSA (mL/m^2^) [75]	Papillary muscles excluded, blood between trabeculae included	58	43.1 ± 8.7	59	38.1 ± 5.9
Trabeculation mass (mass of the trabeculated LV myocardium) per BSA (g/m^2^) [17]	Papillary muscles included, blood between trabeculae excluded	100	23.0 ± 4.7	100	18.2 ± 3.1
Trabeculation mass (mass of the trabeculated LV myocardium) (g) [79]	Papillary muscles and blood between trabeculae excluded	2655	6.6 ± 2.7	4729	4.7 ± 1.9
Trabeculation mass (mass of the trabeculated LV myocardium) per BSA (g/m^2^) [79]	Papillary muscles and blood between trabeculae excluded	2655	3.4 ± 1.3	4729	2.8 ± 1.1

*bSSFP* balanced steady-state free precession, *n* number of study subjects, *SD* standard deviation, *LV* left ventricle, *BSA* body surface area

**Table 54:** Normal fractal dimension (unitless) of left ventricular trabeculation by ethnicity.

Parameter	Reference	Ethnicity	n	Mean ± SD
Global FD	[72]	Black	30	1.246 ± 0.005
	[78]	Chinese	100	1.192 ± 0.032
	[77]	Singaporean Chinese	180	1.205 ± 0.031
	[72]	White	75	1.228 ± 0.002
Maximal apical FD	[72]^a^	Black	30	1.253 ± 0.025
	[78]^b^	Chinese	100	1.264 ± 0.044
	[77]^b^	Singaporean Chinese	180	1.278 ± 0.045
	[72]^a^	White	75	1.235 ± 0.03

*Data are means ± standard deviation. FD* fractal dimension, *n* number of study subjects, *SD* standard deviation

^a^Measured for the apical third of the left ventricle, ^b^measured for the apical half of the left ventricle

**Table 55:** Normal fractal dimension (unitless) of left ventricular trabeculation in adults stratified by BMI according to reference [76].

	BMI ≥ 30 kg/m^2^			BMI ≥ 25 to < 30 kg/m^2^			BMI < 25 kg/m^2^		
Parameter	All (n = 163)	Men (n = 71)	Women (n = 92)	All (n = 206)	Men (n = 108)	Women (n = 98)	All (n = 235)	Men (n = 100)	Women (n = 135)
Max. apical FD^a^ (mean ± SD)	1.203 ± 0.06	1.212 ± 0.07	1.196 ± 0.06	1.194 ± 0.06	1.197 ± 0.05	1.190 ± 0.07	1.169 ± 0.07	1.177 ± 0.06	1.162 ± 0.05

*Data are means ± standard deviation. BMI* body mass index, *n* number of study subjects, *FD* fractal dimension, *SD* standard deviation, *Max.* maximal

^a^Measured for the apical half of the left ventricle

**Age:** Dawson et al. demonstrated a higher trabeculation thickness in the 3rd compared to the 2nd decade followed by a lower trabeculation thickness in each of the following decades till the 7th decade. This trend was significant in the anterior (1, 7, 13) and apical inferior segments, but not in the remainder of segments [69].

For trabeculation mass, no differences were found between age groups in men and women [17].

There is no relationship between ratio of trabeculated/compact myocardial thickness and age [69], [71]. In 140 healthy volunteers between 20 and 69 years, Bentatou et al. showed a significantly greater trabeculation mass and trabeculated/ compact myocardial mass ratio in younger compared to older subjects [13].

In a study by Cai et al., older subjects had higher global fractal dimension [77], while Captur et al. did not find an association between maximal apical fractal dimension and age [76].

**Anthropometric measures:** In a subset of 367 healthy individuals of the MESA cohort, maximal trabeculation/compact thickness ratio was not associated with height or weight [71].

Captur et al. found no significant correlation between the left-ventricular fractal dimension and body-surface area in a multiethnic cohort of 604 healthy individuals [76]. In another study, global fractal dimension was positively associated with height, weight, and BSA in 180 healthy Chinese [77].

**Ethnicity:** In MESA, maximal trabeculation thickness was higher in Blacks and Chinese compared to Whites, while there was no relationship between maximal trabeculation/compact thickness ratio and ethnicity [71].

Using the fractal dimension, ethnicity was shown to influence LV trabeculation parameters, with greater endocardial complexity (i.e. higher fractal dimension) demonstrated in healthy Blacks as opposed to healthy Whites, and greater complexity demonstrated in Whites, African American, and Hispanics compared to Chinese Americans [76].

**Left ventricular volumes and function:** In MESA, participants without cardiac disease or hypertension, lower ejection fraction, and higher left ventricular end-diastolic volume were associated with a greater maximum trabeculation/compact myocardial thickness ratio in multivariable analysis [71]. This is in line with Bentatou et al., who demonstrated that trabeculation mass and trabeculated/compact myocardial mass ratio were negatively correlated to ejection fraction [13].

Regarding endocardial complexity, Captur et al. found no significant correlation between fractal dimension and ejection fraction and left-ventricular end-diastolic and end-systolic volumes after adjustment for age, sex, ethnicity, and BSA [72], [76]. In 180 healthy Chinese, the fractal dimension was associated with indexed left-ventricular end-diastolic and end-systolic volumes independent of age, sex, and BMI, but no association with left-ventricular ejection fraction was found [77].

**Post-processing:** No widely accepted methods has been used for analyzing trabeculation. Several different measurement approaches have been described (Table 50, Fig. 6). Principally these methods measure trabeculation of the left ventricle either in terms of the trabeculated layer’s thickness, mass, volume, or fractal complexity, with or without adjusting for the thickness, mass or volume of the adjacent compacted myocardium.

Reference ranges are influenced by the cardiac phase used to obtain measurements (systole versus diastole), the inclusion or exclusion of papillary muscles in the trabeculated or compact myocardium and the imaging plane (short axis versus long axis).

Where semi-automated segmentation of trabecular contours is undertaken, the type of algorithm used may impact subsequent results. Therefore, methods must specify the algorithm in detail [81].

## 13 Cardiac valves and quantification of flow

### 13.1 Influencing factors

**Gender:** *Aorta peak systolic velocities:* Women had lower aorta systolic velocities across all age groups in the study by Garcia et al. [82] and in the 18-30 years age category in the study by Scott et al. [84]. The differences in aorta peak systolic velocities were insignificant in other age groups in the study by Scott et al. [84].

**Fig. 7:**
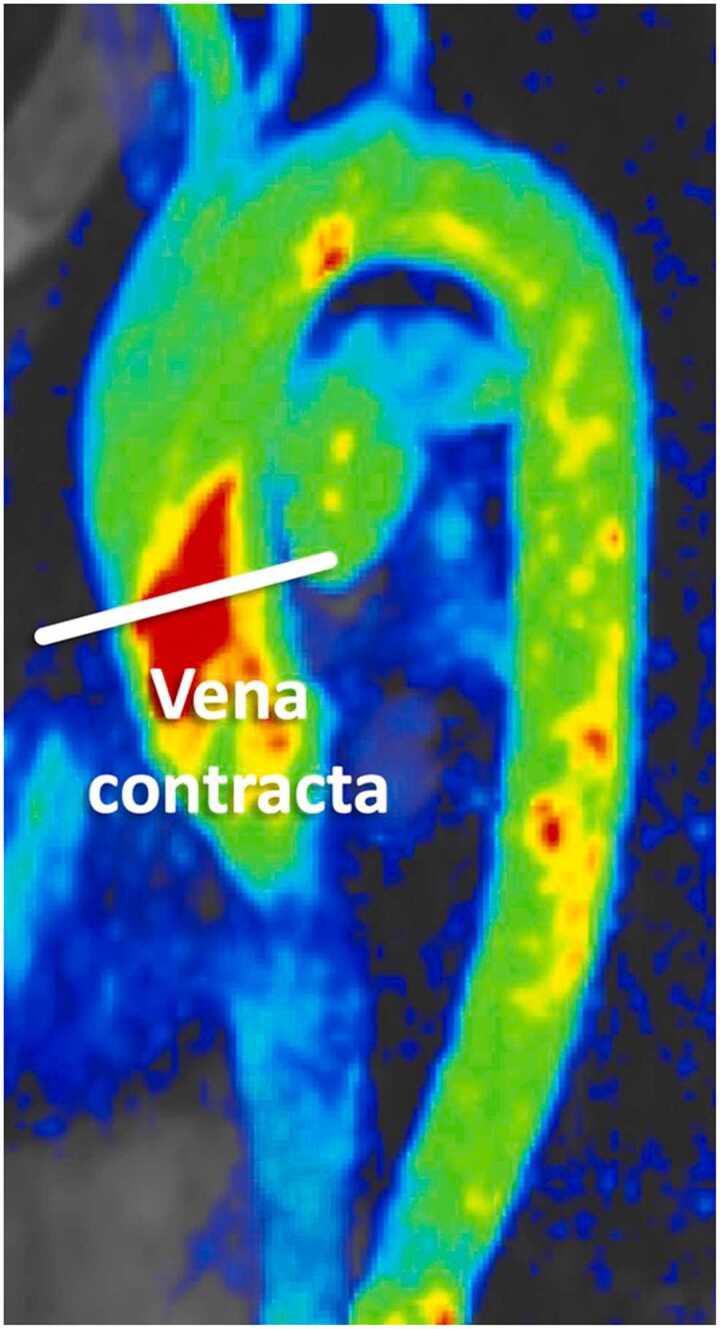
Image of a 4D flow sequence illustrating the site of measurement of peak systolic velocity at the vena contracta level according to reference [82]. *4D* four-dimensional

**Fig. 8:**
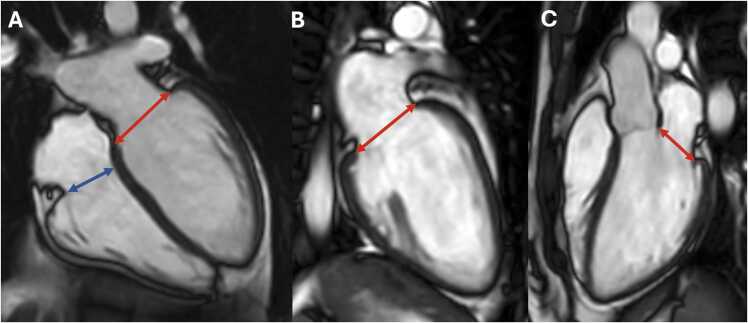
Measurements of mitral valve annulus diameter (red arrow) obtained on a four-chamber view (A), two-chamber view (B), and three-chamber view (C) and tricuspid valve annulus diameter (blue arrow) obtained on a four-chamber view (A) at diastole according to reference [83]

**Table 56:** References. Cardiac valves and quantification of flow.

First author, year n, male: female Age range Study population	CMR acquisition	Parameter, Post-processing (Fig. 7 and 8)
Garcia, 2018 [82] 57:41 9-78 years Volunteers, Ethnicity/Nationality NS	• 1.5T (n = 60), 3.0T (n = 38) • 4D Flow MRI acquired sagittal oblique 3D volume covering aorta with prospective ECG-triggering and respiratory navigator • Spatial resolution = 1.6-2.5 × 1.6-2.5 × 2.2-3.4 mm^3^ • Temporal resolution = 38-43 ms • TE/TR = 2.3-2.8/4.8-5.4 ms • Flip angle = 7-15 degrees • Venc 1.5 m/s • 4D Flow MRI data pre-processing conducted using custom software in MATLAB^a^ to correct Eddy currents, Maxwell terms, and velocity aliasing	• *Aorta peak velocity* measured at the vena contracta, determined from the velocity magnitude maximum intensity projection visualization, downstream from the aortic valve (Fig. 7). • Semiautomatic 3D segmentation of the thoracic aorta was performed with MIMICS*.*^b^
Ricci, 2020 [83] 328:393 45-74 years Caucasian volunteers, UK Biobank	• 1.5T • cine bSSFP • 2ch, 3ch, 4ch	• *Mitral valve annulus diameter* measurements were made by two reviewers at end-systole (frame with largest LA) and end-diastole (frame with smallest LA) on horizontal long-axis (4-chamber), vertical long-axis (2-chamber), and left ventricular outflow tract (three-chamber) views (Fig. 8). • *Anterior mitral valve leaflet length, Alpha and Beta angles, and tenting area* measurements were made by two reviewers at mid-systole on 3-chamber views. • *Tricuspid valve annulus diameter* measurements were made by two reviewers at end-systole and end-diastole on 4-chamber views (Fig. 8). • *Tricuspid to mitral annulus diameter ratio* was calculated from end-diastole measurements.
Scott, 2020 [84] 50:50 18-80 years Volunteers, USA	• 1.5T and 3.0T • 4D Flow MRI acquired sagittal oblique 3D volume covering aorta with prospective ECG-triggering and respiratory navigator • Spatial resolution = 1.7-3.1 × 1.7-3.1 × 2.2-3.3 mm^3^ • Temporal resolution = 38.4-43.2 ms • TE/TR = 2.32-2.85/4.8-5.4 ms • Flip angle = 7-15 degrees • Venc 1.5-2.5 m/s • 4D Flow MRI data pre-processing conducted using custom software in MATLAB^a^ to correct Eddy currents, Maxwell terms, and velocity aliasing	• *Aorta peak velocity* was determined from the maximum velocity at peak systole in the ascending aorta, aortic arch, and descending aorta. • *Aorta peak systolic wall shear stress* was calculated over the entire surface of the aorta. • *Aorta helicity* was calculated during systole. • *Aorta diameter* was calculated from the time-averaged phase contrast angiogram from the 4D Flow MRI data.

*n* number of study subjects, *NS* not specified, *T* Tesla, *TE* echo time, *TR* repetition time, *venc* velocity encoding, *bSSFP* balanced steady-state free precession, *LA* left atrium, *2ch* two-chamber view, *3ch* three-chamber view, *4ch* four-chamber view, *ECG* electrocardiogram

^a^MathWorks, Natick, Massachusetts, USA; ^b^Materialise, Leuven, Belgium

**Table 57:** Stages of valvular heart disease in adults (adapted from echocardiography according to references [92], [93]).

Valve disease	Parameter	Stage Progressive	Severe
Aortic stenosis	Maximum velocity (m/s)	Mild: 2.0-2.9 Moderate: 3.0-3.9	Severe: ≥4 Very severe: ≥5 Low-flow/low-gradient: <4 m/s (at rest)
	Orifice area (cm^2^)		≤1.0
	Orifice area/BSA (cm^2^/m^2^)		≤0.6
Aortic regurgitation	Regurgitant volume (mL/beat)	Mild: <30 Moderate: 30-59	≥60
	Regurgitant fraction (%)	Mild: <30 Moderate: 30-49	≥50
	Effective regurgitant orifice (cm^2^)	Mild: <0.10 Moderate 0.10-0.29	≥0.30
Mitral stenosis	Transmitral flow velocity (m/s)	Increased	
	Orifice area (cm^2^)	>1.5	Severe: ≤1.5 Very severe: ≤1.0
Primary mitral regurgitation	Regurgitant volume (mL)	<60	≥60
	Regurgitant fraction (%)	<50	≥50
	Effective regurgitant orifice (cm^2^)	<0.40	≥0.40
Secondary mitral regurgitation	Regurgitant volume (mL)	<30	≥30
	Regurgitant fraction (%)	<50	≥50
	Effective regurgitant orifice (cm^2^)	<0.20	≥0.20
Pulmonic stenosis	Peak velocity (m/s)		>4
Tricuspid stenosis	Orifice area (cm^2^)		≤1.0

*BSA* body surface area

**Table 58:** Mitral valve flow for determination of diastolic left ventricular function according to reference [94].

Parameter	Normal	Type 1 (impaired relaxation)	Type 2 (pseudonormal)	Type 3 (restrictive, partially reversible)	Type 3 (restrictive, fixed)
MDT (ms)	150-220	Increased	Normal	Decreased	Decreased
E/A ratio	1-2	<1	1-2	>2	>2

*MDT* mitral deceleration time, *E/A ratio* ratio of the mitral early (E) and atrial (A) components of the mitral inflow velocity profile

**Table 59:** Normal mean aortic valve peak velocity (m/s) by 4D-flow.

Reference	Measurement location	Age (years)	n	Mean ± SD
[82]	Where the transvalvular velocity reaches its maximum during peak systole (Fig. 7).	9-15	9	1.3 ± 0.1
		16-20	13	1.3 ± 0.2
		21-39	27	1.1 ± 0.2
		40-59	40	1.3 ± 0.2
		>60	9	1.4 ± 0.3
[84]	Ascending aorta (between aortic valve and takeoff of the innominate artery)	18-30	20	1.5 ± 0.2
		31-40	20	1.4 ± 0.2
		41-50	20	1.5 ± 0.3
		51-60	20	1.5 ± 0.3
		61-80	20	1.6 ± 0.3

*Data are means ± standard deviation. n* number of study subjects, *SD* standard deviation

**Table 60:** Normal absolute and indexed (BSA) mitral valve annulus diameter for men and women measured on two-, three-, and four-chamber cine bSSFP at diastole and systole according to reference [83].

			Men (n = 328)		Women (n = 393)	
Parameter	Cardiac phase	Imaging plane	Mean ± SD	LL-UL	Mean ± SD	LL-UL
MVAD (cm)	End-diastole	4ch	3.2 ± 0.4	2.4-4.0	2.9 ± 0.4	2.1-3.7
	End-systole	4ch	3.3 ± 0.5	2.3-4.3	3.1 ± 0.4	2.3-3.9
	End-diastole	3ch	2.9 ± 0.4	2.1-3.7	2.6 ± 0.4	1.8-3.4
	End-systole	3ch	3.3 ± 0.4	2.5-4.1	3.0 ± 0.4	2.2-3.8
	End-diastole	2ch	3.6 ± 0.4	2.8-4.4	3.2 ± 0.3	2.6-3.8
	End-systole	2ch	3.7 ± 0.4	2.9-4.5	3.3 ± 0.4	2.5-4.1
MVAD/BSA (cm/m^2^)	End-diastole	4ch	1.6 ± 0.2	1.2-2.0	1.8 ± 0.2	1.4-2.2
	End-systole	4ch	1.7 ± 0.3	1.1-2.3	1.8 ± 0.3	1.2-2.4
	End-diastole	3ch	1.5 ± 0.2	1.1-1.9	1.6 ± 0.2	1.2-2.0
	End-systole	3ch	1.7 ± 0.2	1.3-2.1	1.8 ± 0.2	1.4-2.2
	End-diastole	2ch	1.8 ± 0.2	1.4-2.2	1.9 ± 0.2	1.5-2.3
	End-systole	2ch	1.9 ± 0.2	1.5-2.3	1.9 ± 0.2	1.5-2.3

*Data are means ± standard deviation. BSA* body surface area, *bSSFP* balanced steady-state free precession, *n* number of study subjects, *SD* standard deviation, *MVAD* mitral valve annulus diameter, *LL* lower limit, *UL* upper limit, *4ch* four-chamber view, *3ch* three-chamber view, *2ch* two-chamber view

**Table 61:** Normal absolute and indexed (BSA) tricuspid valve annulus diameter for men and women measured on four-chamber cine bSSFP at diastole and systole according to reference [83].

		Men (n = 328)		Women (n = 393)	
Parameter	Cardiac phase	Mean ± SD	LL-UL	Mean ± SD	LL-UL
TVAD (cm)	End-diastole	3.2 ± 0.5	2.2-4.2	2.9 ± 0.4	2.1-3.7
	End-systole	3.2 ± 0.5	2.2-4.2	2.8 ± 0.4	2.0-3.6
TVAD/BSA (cm/m^2^)	End-diastole	1.6 ± 0.3	1.0-2.2	1.7 ± 0.2	1.3-2.1
	End-systole	1.7 ± 0.3	1.1-2.3	1.7 ± 0.3	1.1-2.3

*Data are means ± standard deviation. BSA* body surface area, *bSSFP* balanced steady-state free-precession, *n* number of study subjects, *SD* standard deviation, *LL* lower limit, *UL* upper limit, *TVAD* tricuspid valve annulus diameter

*Mitral annulus:* Women had smaller absolute mitral annulus diameters (2ch-, 3ch-, and 4ch-view) and anterior mitral valve leaflet length than men [83].

*Tricuspid annulus:* Women had smaller absolute tricuspid annulus diameters and end-diastole tricuspid annulus to mitral annulus diameter ratios than men [83].

**Age:** *Aorta peak systolic velocities:* In the study by Garcia et al. [82], peak ascending aorta systolic velocities decrease between 9 and 15 years to the 21 and 39 years groups and then increases with age in each of the subsequent age groups. In the study by Scott et al. [84], peak ascending aorta systolic velocities decrease between 18 and 30 years to 31 and 40 years groups and then increase with age up to 80 years.

*Mitral annulus:* Mitral annulus diameters were not influenced by age [83].

*Tricuspid annulus:* Tricuspid annulus and end-diastole tricuspid annulus to mitral annulus diameter ratios increase with age in men and women [83].

**Anthropometric measures:** Body size and heart rate can influence the velocity-to-noise ratio (VNR) of flow-sensitive MRI acquisitions [85] and should be adjusted to optimize the VNR in each individual patient.

*Mitral annulus:* After indexing for body surface area, women had larger indexed mitral annulus diameters and anterior mitral valve leaflet length than men [83]. After indexing for height, women had smaller indexed mitral annulus diameters (2ch-view only) than men [83].

*Tricuspid annulus:* After indexing for body surface area, women had larger indexed tricuspid annulus diameters than men [83]. After indexing for height, women had smaller indexed tricuspid annulus diameters and end-diastole tricuspid annulus to mitral annulus diameter ratios than men [83].

**CMR acquisition and post-processing:** A thorough description of the acquisition and post-processing factors influencing velocity-encoding MRI is beyond the scope of this article and can be found in numerous previously published review articles on this topic. Briefly, the image quality, therefore the accuracy of flow measurements, of flow MRI is affected by body size, anatomy, velocity encoding limit, presence of intravenous contrast, acquisition parameters (e.g., spatial resolution, flip angle, repetition time, and echo time). It is recommended that at least six voxels cover the vessel diameter to improve the accuracy of flow measurements [86].

Peak velocities obtained with different MRI sequences vary, with some studies showing higher velocities obtained with 4D Flow MRI relative to 2D Flow MRI and transthoracic echocardiography and other studies showing lower velocities obtained with 4D Flow MRA relative to 2D Flow MRI and transthoracic echocardiography. Peak velocities in the ascending aorta measured with 4D Flow MRI were higher than those acquired with one-directional velocity-encoded 2D Flow MRI in healthy volunteers [87] and patients with aortic valve stenosis [88]. Peak velocities in the ascending aorta measured with 4D Flow MRI were higher than those acquired with one-directional velocity-encoded 2D Flow MRI in patients with aortic valve stenosis [89].

In a multi-vendor, multi-center study in 15 healthy volunteers, Demir et al. found no differences in forward flow or peak systolic velocity at the level of the sinotubular junction between three manufacturers’ scanners [90]. Significant differences in forward flow and peak systolic velocities were observed at other locations in the ascending and descending thoracic aorta. In scan-rescan testing, Demir et al. found no differences in peak velocity or forward flow volumes in the aorta [90].

Before post-processing of 4D Flow MRI, data should be pre-processed for background correction and correction of phase wraps, if necessary.

Oechtering et al. analyzed 4D Flow MRI data acquired in eight healthy volunteers scanned twice on two different vendors’ 3T scanner with four different 4D Flow analysis software programs. Intra- and inter-reader variability in flow volumes and velocities differed between software programs [91].

## 14 Normal dimensions of the thoracic aorta in adults

### 14.1 Influencing factors

**Gender:** The included studies have different CMR acquisition and post-processing methods (Table 62). In all studies, males consistently have larger absolute aortic root (annulus, sinus, and sinotubular junction) and thoracic aortic dimensions (ascending and proximal/distal descending aorta) compared to females [26], [27], [95], [96], [97], [98]. Wall area and thickness were similar in both sexes [27]. After accounting for the effects of BSA, indexed annular diameters were similar between sexes [26] but males have similar or smaller aortic sinus and sinotubular junction compared to females [26], [95], [96].

**Table 62:** References. Normal dimensions of the thoracic aorta in adults.

First author, year n, male:female Age range Study population	CMR acquisition	Post-processing (Fig. 9, Fig. 10, Fig. 11)
Burman, 2008 [95] 60:60 20-80 years Volunteers, UK	• 1.5T, cine bSSFP • Systole and diastole • Cross-sectional images at the level of the aortic sinus and oblique sagittal and oblique coronal LVOT images.	• Luminal diameter calculated as the average of 3 cusp-commissure and 3 cusp-cusp diameters, respectively, measured on cross-sectional images of the aortic sinus (Fig. 10). • Luminal diameter at the level of the anulus, sinus, and the sinotubular junction measured on the oblique sagittal and oblique coronal LVOT plane (Fig. 11).
Davis, 2014 [96] 208:239 19-70 years Volunteers, UK	• 1.5T, cine bSSFP • Diastole • Cross-sectional images at 3 levels (ascending aorta, proximal, and distal descending aorta) and sagittal LVOT plane	• Maximal luminal diameter in mm calculated as 2 * √Area(mm^2^)/π based on measurements of the area on cross-sectional images using an in-house Matlab-based automated edge definition software. • Luminal diameter measured at 3 levels (annulus, sinus, sinotubular junction) on the sagittal LVOT plane (Fig. 11).
Turkbey, 2014 [97] 770:842 45-84 years MESA, USA	• 1.5T, phase contrast (magnitude image) • Axial plane at the level of the right pulmonary artery	• Mean luminal diameter of the ascending aorta calculated as the average of the maximum and the minimum diameter based on automatically traced, manually corrected contours to determine maximum and minimum cross-sectional area using QFLOW software.^a^
Eikendal, 2016 [27] 59:65 25-35 years AMBITYON study, Netherlands	• 3T, fat suppressed 3D-T1-black blood VISTA • Sagittal plane	• Luminal and total vessel diameter and area, vessel wall area, mean and maximal wall thickness, and ratio of wall area/total vessel area of the descending thoracic aorta were automatically calculated by Vessel Mass software^b^ after manual tracing of the luminal and outer aortic wall on axial reformatted images with a slice thickness of 1.2 mm.
Le 2016 [26] 91:89 20-69 years Volunteers, Singapore	• 3T, cine bSSFP • Diastole • Sagittal LVOT plane	• Luminal diameter measured at 3 levels (annulus, sinus, and sinotubular junction) (Fig. 11).
Li 2021 [98] 100:100 20-70 years Volunteers, China	• 3T • HASTE, axial plane at the level of the widest diameter of the main pulmonary artery • Cine bSSFP, systole, sagittal LVOT plane	• Diameter of the ascending and descending aorta measured on axial HASTE images. • Luminal diameter of the aortic sinus measured on the sagittal LVOT plane at systole (Fig. 11).

*n* number of study subjects, *bSSFP* balanced steady-state free precession, *LVOT* left ventricular outflow tract, *MESA* Multi-Ethnic Study of Atherosclerosis, *VISTA* volume isotropic turbo spin echo acquisition, *AMBITYON* Atherosclerosis Monitoring and Biomarker measurements in the Young, *HASTE* half Fourier single-shot turbo spin-echo

^a^Medis Medical Imaging Systems, Leiden, The Netherlands; ^b^Laboratory for Clinical and Experimental Image Processing, The Netherlands

**Fig. 9:**
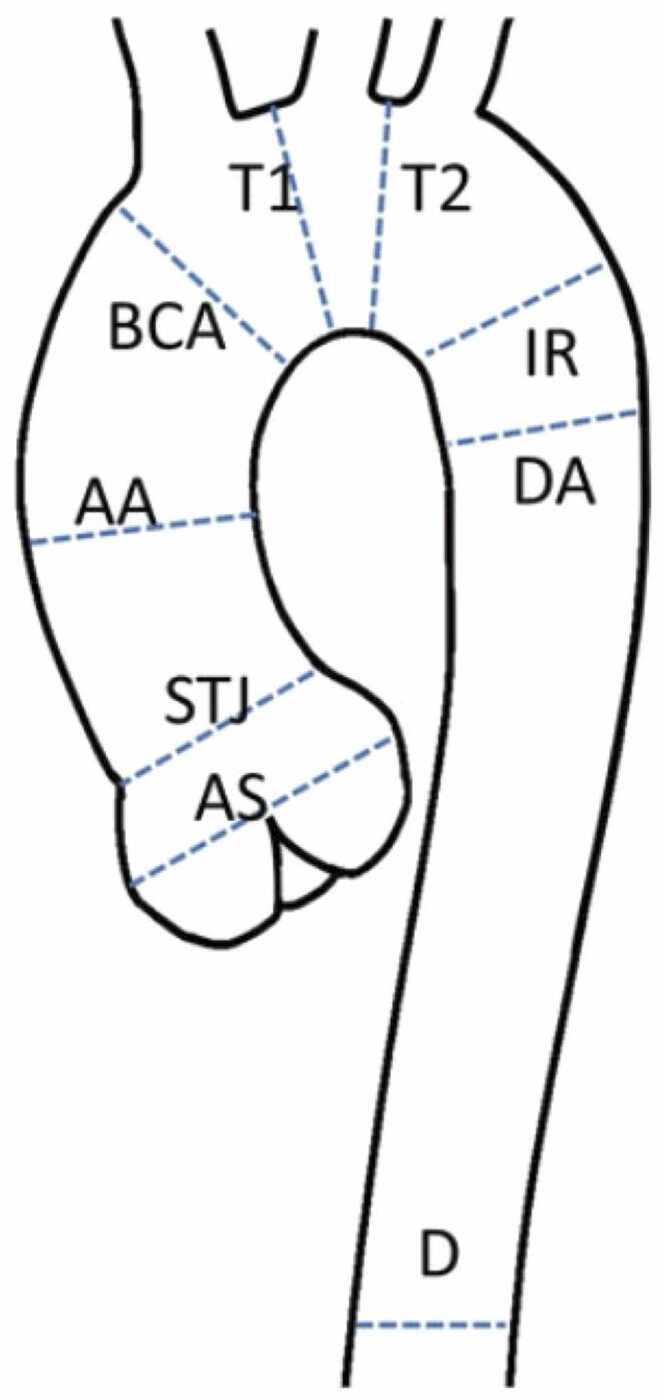
Sites of measurement of the thoracic aorta. *AS* aortic sinus, *STJ* sinotubular junction, *AA* ascending aorta, *BCA* proximal to the origin of the brachiocephalic artery, *T1* between the origin of the brachiocephalic artery and the left common carotid artery, *T2* between the origin of the left common carotid artery and the left subclavian artery, *IR* isthmic region, *DA* descending aorta, *D* thoracoabdominal aorta at the level of the diaphragm

**Fig. 10:**
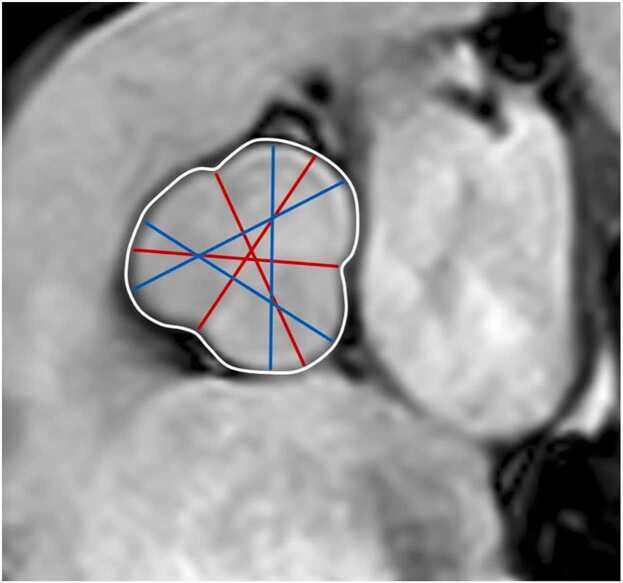
Cusp-commissure (red lines) and cusp-cusp (blue lines) measurements at the level of the aortic sinus according to reference [95]

**Fig. 11:**
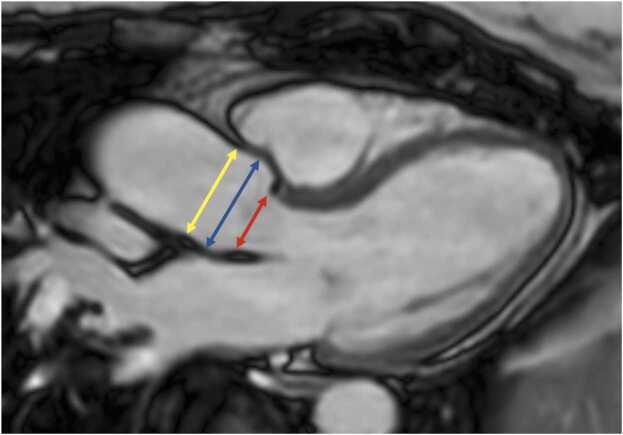
Measurements of the diastolic diameters of the aortic annulus (red arrow), the aortic sinus (blue arrow), and the sinotubular junction (yellow arrow) on a left ventricular outflow tract view obtained with a balanced steady-state free precession sequence

**Table 63:** Normal absolute and indexed (BSA) aortic sinus luminal diameters and area for men and women on cross-sectional bSSFP at diastole according to [95].^a^

		Men		Women	
Parameter	Age (years)	n	Mean ± SD (LL-UL)	n	Mean ± SD (LL-UL)
Aortic sinus diameter (cusp-commissure) (mm)	20-29	10	30.4 ± 3.3	10	26.3 ± 3.9
	30-39	10	29.7 ± 3.5	10	26.8 ± 2.8
	40-49	10	31.6 ± 1.6	10	30.0 ± 2.1
	50-59	10	32.7 ± 4.8	10	28.4 ± 1.8
	60-69	10	33.5 ± 2.3	10	29.5 ± 2.0
	70-79	10	33.9 ± 3.0	10	29.6 ± 1.4
	*All*	*60*	*32.0* ± *3.5* *(25-39)*	*60*	*28.4* ± *2.8* *(23-34)*
Aortic sinus diameter (cusp-commissure)/BSA (mm/m^2^)	20-29	10	15.6 ± 1.7	10	15.3 ± 2.0
	30-39	10	15.1 ± 1.6	10	16.4 ± 1.3
	40-49	10	15.3 ± 1.0	10	16.8 ± 2.3
	50-59	10	16.6 ± 1.9	10	17.2 ± 1.4
	60-69	10	17.2 ± 1.7	10	17.1 ± 1.4
	70-79	10	17.4 ± 1.2	10	17.8 ± 0.9
	*All*	*60*	*16.2* *±* *1.8* *(12.6-19.8)*	*60*	*16.8* *±* *1.7* *(13.4-20.2)*
Aortic sinus diameter (cusp-cusp) (mm)	20-29	10	32.8 ± 3.8	10	28.4 ± 4.7
	30-39	10	32.0 ± 3.9	10	28.7 ± 3.0
	40-49	10	34.1 ± 2.3	10	32.8 ± 2.5
	50-59	10	35.2 ± 5.7	10	30.6 ± 2.6
	60-69	10	36.2 ± 2.5	10	32.0 ± 2.2
	70-79	10	37.0 ± 3.5	10	32.0 ± 1.6
	*All*	*60*	*34.6* *±* *4.0* *(27-43)*	*60*	*30.7* *±* *3.3* *(24-37)*
Aortic sinus diameter (cusp-cusp)/BSA (mm/m^2^)	20-29	10	16.9 ± 1.9	10	16.6 ± 2.3
	30-39	10	16.2 ± 1.9	10	17.6 ± 1.3
	40-49	10	16.5 ± 1.3	10	18.4 ± 2.5
	50-59	10	17.9 ± 2.3	10	18.5 ± 1.8
	60-69	10	18.6 ± 2.0	10	18.6 ± 1.6
	70-79	10	19.0 ± 1.3	10	19.3 ± 1.0
	*All*	*60*	*17.5* ± *2.0* *(13.5-21.5)*	*60*	*18.1* ± *2.0* *(14.1-22.1)*
Aortic sinus area (cm^2^)	20-29	10	7.5 ± 1.8	10	5.6 ± 1.8
	30-39	10	7.1 ± 1.9	10	5.7 ± 1.2
	40-49	10	8.2 ± 1.1	10	7.3 ± 1.2
	50-59	10	8.8 ± 2.9	10	6.5 ± 1.0
	60-69	10	9.1 ± 1.3	10	7.0 ± 1.0
	70-79	10	9.7 ± 1.7	10	7.0 ± 0.8
	*All*	*60*	*8.4* ± *2.0* *(4.4-12.4)*	*60*	*6.5* ± *1.3* *(3.9-9.1)*
Aortic sinus area/BSA (cm^2^/m^2^)	20-29	10	3.9 ± 0.8	10	3.3 ± 0.9
	30-39	10	3.6 ± 0.9	10	3.5 ± 0.6
	40-49	10	3.9 ± 0.5	10	4.1 ± 0.9
	50-59	10	4.5 ± 1.2	10	3.9 ± 0.6
	60-69	10	4.7 ± 0.8	10	4.0 ± 0.6
	70-79	10	4.9 ± 0.6	10	4.2 ± 0.4
	*All*	*60*	*4.2* ± *0.9* *(2.4-6.0)*	*60*	*3.8* ± *0.8* *(2.2-5.4)*

*Data are means ± standard deviation. BSA* body surface area, *bSSFP* balanced steady-state free precession, *n* number of study subjects, *SD* standard deviation, *LL* lower limit, *UL* upper limit

^a^Measurements obtained according to Fig. 10

**Table 64:** Normal luminal thoracic aortic diameters for men and women on cross-sectional bSSFP at diastole according to [96].

Level	Men (n = 208) Mean ± SD (LL-UL)	Women (n = 239) Mean ± SD (LL-UL)
Ascending aorta diameter (mm)	26.7 ± 3.9 (19-35)	25.5 ± 3.7 (18-33)
Proximal descending aorta diameter (mm)	20.6 ± 2.8 (15-26)	18.9 ± 2.0 (15-23)
Distal descending aorta diameter (mm)	17.6 ± 2.6 (12-23)	16.4 ± 2.0 (12-20)

*Data are means ± standard deviation. bSSFP* balanced steady-state free-precession, *n* number of study subjects, *SD* standard deviation, *LL* lower limit, *UL* upper limit

**Table 65:** Normal absolute and indexed (BSA) ascending aorta luminal diameter^a^ for men and women by age according to [97].

Parameter	Age (years)	Men (n = 770) Median (5^th^-95^th^ percentile)	Women (n = 842) Median (5^th^-95^th^ percentile)
Ascending aorta diameter (mm)	45-54	32 (27-37)	29 (25-34)
	55-64	33 (28-41)	30 (26-36)
	65-74	34 (29-41)	31 (26-36)
	75-84	35 (29-41)	31 (27-37)
Ascending aorta diameter/BSA (mm/m^2^)	45-54	16 (13-20)	17 (14-21)
	55-64	17 (14-21)	18 (15-22)
	65-74	18 (14-22)	18 (15-22)
	75-84	19 (15-23)	20 (15-28)

*Data are presented as median and 5th and 95th percentiles. BSA* body surface area, *n* number of study subjects

^a^Measurements obtained on cross-sectional magnitude images of a phase contrast sequence

**Table 66:** Normal descending thoracic aortic diameter, area, and wall thickness^a^ in young men and women (25-35 years) according to [27].

Parameter	Men (n = 59) Median (10^th^-90^th^ percentile)	Women (n = 65) Median (10^th^-90^th^ percentile)
Luminal diameter (mm)	19 (17-21)	17 (16-19)
Total diameter (mm)	22 (20-24)	20 (19-22)
Luminal area (cm^2^)	2.9 (2.2-3.5)	2.3 (2.0-2.8)
Total area (cm^2^)	3.9 (3.1-4.6)	3.3 (2.8-3.9)
Wall area (cm^2^)	1.0 (0.8-1.2)	1.0 (0.8-1.1)
Wall thickness (mm)	1.5 (1.4-1.8)	1.5 (1.4-1.9)

*Data are presented as median and 5th and 95th precentile. n* number of study subjects

^a^Measurements obtained on axial reformatted images of a fat suppressed three-dimensional-T1-black blood volume isotropic turbo spin echo acquisition sequence

**Table 67:** Normal aortic root diameters in men and women by age measured on sagittal left ventricular outflow tract bSSFP at diastole.^a^

			Men		Women	
Parameter	Age (years)	References	n	Mean ± SD (LL-UL)	n	Mean ± SD (LL-UL)
Aortic annulus diameter (mm)	20-29	[26], [95]	29	22.3 ± 2.2	26	19.1 ± 1.7
	30-39	[26], [95]	27	21.9 ± 2.3	28	19.0 ± 1.8
	40-49	[26], [95]	29	21.9 ± 1.6	27	20.0 ± 1.6
	50-59	[26], [95]	30	22.1 ± 2.0	30	19.4 ± 1.6
	60-69	[26], [95]	26	23.2 ± 1.7	28	19.7 ± 1.7
	70-79	[95]	10	23.3 ± 2.7	10	20.2 ± 1.5
	*All*	[26], [95], [96]	*359*	*23.0* ± *4.4* *(14-32)*	*388*	*20.0* ± *3.1(14-26)*
Aortic annulus diameter/BSA (mm/m^2^)	20-29	[26]	19	12.0 ± 1.3	16	12.0 ± 1.2
	30-39	[26]	17	12.0 ± 1.2	18	12.0 ± 0.8
	40-49	[26]	19	12.0 ± 0.9	17	13.0 ± 0.8
	50-59	[26]	20	12.0 ± 1.2	20	12.0 ± 0.8
	60-69	[26]	16	13.0 ± 0.9	18	13.0 ± 1.0
	*All*	[26]	*91*	*12.2* ± *1.1* *(10-14)*	*89*	*12.0* ± *1.0* *(10-14)*
Aortic sinus diameter (mm)	20-29	[26], [95]	29	30.1 ± 3.0	26	25.0 ± 3.5
	30-39	[26], [95]	27	30.7 ± 3.2	28	25.3 ± 2.9
	40-49	[26], [95]	29	32.0 ± 3.2	27	29.2 ± 3.8
	50-59	[26], [95]	30	31.4 ± 4.0	30	28.0 ± 2.8
	60-69	[26], [95]	26	33.3 ± 3.4	28	28.6 ± 3.9
	70-79	[95]	10	35.1 ± 3.7	10	30.2 ± 2.0
	*All*	[26], [95], [96]	*359*	*31.9* ± *6.3* *(20-44)*	*388*	*27.5* ± *5.1(18-38)*
Aortic sinus diameter/BSA (mL/m^2^)	20-29	[26], [95]	29	15.9 ± 2.1	26	15.1 ± 1.9
	30-39	[26], [95]	27	15.6 ± 1.8	28	15.7 ± 1.8
	40-49	[26], [95]	29	16.3 ± 1.8	27	17.9 ± 2.1
	50-59	[26], [95]	30	17.7 ± 2.1	30	17.1 ± 1.5
	60-69	[26], [95]	26	17.7 ± 1.8	28	17.8 ± 1.8
	70-79	[95]	10	18.0 ± 1.2	10	18.2 ± 0.9
	*All*	[26], [95]	151	*16.7* ± *1.9* *(13-20)*	*149*	*16.9* ± *1.8(13-20)*
Sinotubular junction diameter (mm)	20-29	[26], [95]	29	22.3 ± 2.6	26	20.3 ± 2.7
	30-39	[26], [95]	27	22.9 ± 3.1	28	20.3 ± 2.8
	40-49	[26], [95]	29	25.5 ± 2.8	27	23.3 ± 4.1
	50-59	[26], [95]	30	26.1 ± 2.4	30	23.0 ± 2.8
	60-69	[26], [95]	26	26.6 ± 3.5	28	23.9 ± 3.1
	70-79	[95]	10	28.3 ± 2.7	10	25.0 ± 2.0
	*All*	[26], [95], [96]	*359*	*24.9* ± *5.9* *(13-37)*	*388*	*22.2* ± *4.7(13-32)*
Sinotubular junction diameter/BSA (mm/m^2^)	20-29	[26]	19	12.0 ± 1.2	16	13.0 ± 1.4
	30-39	[26]	17	12.0 ± 1.3	18	12.0 ± 1.2
	40-49	[26]	19	14.0 ± 1.1	17	14.0 ± 1.5
	50-59	[26]	20	15.0 ± 1.4	20	14.0 ± 1.7
	60-69	[26]	16	15.0 ± 1.8	18	15.0 ± 1.6
	*All*	[26]	91	*13.4* ± *1.8* *(10-17)*	89	*13.6* ± *1.8* *(10-17)*

*Data are means ± standard deviation. bSSFP* balanced steady-state free-precession, *n* number of study subjects included, *SD* standard deviation, *LL* lower limit, *UL* upper limit, *BSA* body surface area

Reference [26] provided additional data for analysis

^a^Measurements obtained as shown in Fig. 11

**Age:** Aortic root and ascending aortic dimensions increase with greater age [26], [95], [96]. Indexing aortic diameters to BSA attenuated the association between age and aortic dimensions [26], [95], [96].

**Ethnicity:** In the MESA study, small differences in aortic dimensions were observed in Chinese and African Americans compared to Caucasians [97]. These differences between ethnicities were unlikely to be clinically significant.

**CMR acquisition and post-processing:** Aortic measurements vary with different MR acquisition techniques [99] and are larger in systole than diastole [100]. For quantitative analysis, the same sequence and post-processing approaches should be taken to track disease progression. The SCMR recommends measurement on double oblique Multiplanar Reconstruction of images perpendicular to the vessel centerline. Electrocardiogram (ECG)-gated acquisitions are necessary [97]. Measurement of outer diameter is recommended in the case of aortic dilatation (e.g., aneurysms) and inner diameter measurement is recommended in areas of narrowing (e.g., coarctation). For the aortic sinus, absolute and indexed cusp-cusp measurements were larger than cusp-commissure measurements. Similar findings were observed in either sex [95]

## 15 Normal dimensions of the thoracic aorta in children

### 15.1 Influencing factors

Three studies were included in the review with different acquisition protocols and post-processing methodologies (Table 68). Aortic diameters and areas did not vary with sex [102], [103] but varied with BSA [102], [103]. Reference ranges in children are typically presented in z-score or LMS parameters to calculate z-score (Tables 69 and 70). Regression formulas for aortic diameters and area in children were also given to account for BSA (Tables 71 and 72). Similar to adults, measurement of the aorta is influenced by acquisition and post-processing methodologies. A consistent approach is therefore essential to follow-up individuals for disease progression.

**Table 68:** References. Normal dimensions of the thoracic aorta in children.

First author, year n, male:female Age range Study population	CMR acquisition	Post-processing
Kaiser, 2008 [101] 30:23 2-20 years Children with previous history of malignancy, normal CV anatomy, no CV disease, and normal body size, Switzerland	• 1.5T; contrast enhanced MR-angiography (3D FSPGR)	• Shortest diameter measured on cross-sectional reformatted images at 9 locations (Fig. 9).
Kutty, 2012 [102] 55:50 4-20 years Young volunteers, Germany	• 1.5T; magnitude image of a through-plane free-breathing phase contrast sequence	• Cross-sectional area calculated as πr_1_r_2_ (r = radii) based on semiautomatic measurement of the maximal external aortic diameter perpendicular to the vessel and perpendicular to the maximal diameter in systole 1-2 cm distal to the sinotubular junction.
Voges, 2012 [103] 30:41 2-28 years Young volunteers + 5 children with neurologic disorders but free from cardiovascular disease, Germany	• 3T; cine gradient echo • Cross-sectional images at 4 levels (ascending aorta, aortic arch, aortic isthmus, descending aorta above the diaphragm)	• Cross-sectional area measured at maximal distension of the aorta.

*n* number of study subjects, *CV* cardiovascular, *FSPGR* fast spoiled gradient echo sequence

**Table 69:** LMS parameters to calculate z-scores for aortic cross-sectional area^a^ relative to age for boys according to reference [103].

	Ascending aorta			Aortic arch			Aortic isthmus			Descending aorta^b^		
Age^c^	L	M	S	L	M	S	L	M	S	L	M	S
<1	0.3091	91.5360	0.1207	0.8668	80.1737	0.1898	0.1267	53.0050	0.1987	1.5823	44.6080	0.1100
1	0.3091	120.6960	0.1274	0.8668	101.7001	0.1897	0.1267	68.7198	0.1974	1.5823	57.0317	0.1115
2	0.3091	149.8560	0.1341	0.8668	123.2265	0.1895	0.1267	84.4347	0.1960	1.5823	69.4554	0.1129
3	0.3091	179.0160	0.1408	0.8668	144.7529	0.1894	0.1267	100.1495	0.1946	1.5823	81.8791	0.1143
4	0.3091	208.1812	0.1475	0.8668	166.2791	0.1893	0.1267	115.8653	0.1932	1.5823	94.3035	0.1158
5	0.3091	238.3791	0.1542	0.8668	187.7555	0.1891	0.1267	131.7743	0.1918	1.5823	106.8833	0.1172
6	0.3091	272.8715	0.1604	0.8668	208.8732	0.1890	0.1267	148.2790	0.1904	1.5823	119.9057	0.1186
7	0.3091	311.2493	0.1660	0.8668	229.2411	0.1888	0.1267	164.9648	0.1891	1.5823	133.0488	0.1201
8	0.3091	346.8686	0.1707	0.8668	248.8676	0.1887	0.1267	180.7624	0.1877	1.5823	145.5984	0.1215
9	0.3091	380.0230	0.1748	0.8668	268.0557	0.1886	0.1267	195.7825	0.1863	1.5823	157.5124	0.1229
10	0.3091	413.8181	0.1782	0.8668	287.2956	0.1884	0.1267	210.6578	0.1849	1.5823	169.3366	0.1244
11	0.3091	446.7220	0.1812	0.8668	306.7317	0.1883	0.1267	225.5414	0.1835	1.5823	181.3951	0.1258
12	0.3091	476.5703	0.1841	0.8668	326.2205	0.1881	0.1267	240.3324	0.1822	1.5823	193.8192	0.1272
13	0.3091	501.7973	0.1870	0.8668	345.4511	0.1880	0.1267	254.6975	0.1808	1.5823	206.4812	0.1287
14	0.3091	524.0769	0.1902	0.8668	364.2701	0.1879	0.1267	268.8289	0.1794	1.5823	219.2939	0.1301
15	0.3091	546.3695	0.1937	0.8668	382.7610	0.1877	0.1267	282.9653	0.1780	1.5823	232.0152	0.1316
16	0.3091	569.8955	0.1972	0.8668	400.9805	0.1876	0.1267	296.9424	0.1766	1.5823	244.3629	0.1330
17	0.3091	594.7536	0.2003	0.8668	418.9724	0.1875	0.1267	310.5833	0.1752	1.5823	256.2294	0.1344
18	0.3091	620.9611	0.2025	0.8668	436.7805	0.1873	0.1267	323.7094	0.1739	1.5823	267.5155	0.1359
19	0.3091	647.1204	0.2034	0.8668	454.4484	0.1872	0.1267	336.0814	0.1725	1.5823	278.0681	0.1373
20	0.3091	670.2706	0.2030	0.8668	472.0177	0.1871	0.1267	347.4348	0.1711	1.5823	287.6962	0.1387
21	0.3091	690.0681	0.2014	0.8668	489.5219	0.1869	0.1267	357.7775	0.1697	1.5823	296.3958	0.1402
22	0.3091	706.8583	0.1990	0.8668	506.9924	0.1868	0.1267	367.1860	0.1683	1.5823	304.2102	0.1416
23	0.3091	720.9831	0.1960	0.8668	524.4603	0.1866	0.1267	375.7366	0.1670	1.5823	311.1823	0.1430
24	0.3091	732.2902	0.1926	0.8668	541.9124	0.1865	0.1267	383.4824	0.1656	1.5823	317.3075	0.1445
25	0.3091	740.4053	0.1889	0.8668	559.3076	0.1864	0.1267	390.6086	0.1642	1.5823	322.5658	0.1459
26	0.3091	747.1815	0.1849	0.8668	576.7470	0.1862	0.1267	397.7409	0.1628	1.5823	327.1568	0.1473
27	0.3091	754.8518	0.1805	0.8668	594.3196	0.1861	0.1267	405.3735	0.1614	1.5823	331.4000	0.1488
28	0.3091	763.4054	0.1758	0.8668	611.9863	0.1860	0.1267	413.3799	0.1601	1.5823	335.4719	0.1502
29	0.3091	772.1960	0.1711	0.8668	629.6783	0.1858	0.1267	421.4867	0.1587	1.5823	339.4979	0.1516
30	0.3091	780.9891	0.1663	0.8668	647.3706	0.1857	0.1267	429.5945	0.1573	1.5823	343.5234	0.1531

Values represent LMS parameters. *LMS* L = Lambda (skewness of the distribution), M = Mu (median), S = Sigma (variance)

z-score = [(X/M)L − 1]/(L*S), where X is the measured aortic area in mm^2^ and L, M, and S are the values interpolated for the child’s age; lower and upper limits correspond to a z-score of −2 and 2

^a^Aortic area measured at maximum distension of the aorta on cross sectional cine gradient echo images acquired perpendicular to the aortic axis (n = 30), ^b^measured above the diaphragm, ^c^ age in years

**Table 70:** LMS parameters to calculate z-scores for aortic cross-sectional area^a^ relative to age for girls according to reference [103].

	Ascending aorta			Aortic arch			Aortic isthmus			Descending aorta^b^		
Age^c^	L	M	S	L	M	S	L	M	S	L	M	S
<1	−0.7876	121.1903	0.2152	−2.1750	73.6299	0.2114	0.1033	60.0696	0.1621	0.9371	41.0795	0.1398
1	−0.7876	145.9923	0.2140	−2.1750	92.7307	0.2089	0.1033	72.6142	0.1617	0.9371	52.4930	0.1398
2	−0.7876	170.7944	0.2127	−2.1750	111.8315	0.2064	0.1033	85.1587	0.1613	0.9371	63.9065	0.1398
3	−0.7876	195.5999	0.2114	−2.1750	130.9296	0.2039	0.1033	97.7032	0.1609	0.9371	75.3185	0.1398
4	−0.7876	220.4539	0.2102	−2.1750	149.9904	0.2013	0.1033	110.2465	0.1605	0.9371	86.7100	0.1398
5	−0.7876	245.4281	0.2089	−2.1750	168.9588	0.1988	0.1033	122.7870	0.1601	0.9371	98.0510	0.1398
6	−0.7876	270.5738	0.2076	−2.1750	187.8089	0.1963	0.1033	135.3263	0.1597	0.9371	109.3784	0.1398
7	−0.7876	295.9027	0.2064	−2.1750	206.5696	0.1938	0.1033	147.8724	0.1593	0.9371	120.8531	0.1398
8	−0.7876	321.3290	0.2051	−2.1750	225.2367	0.1913	0.1033	160.3915	0.1588	0.9371	132.5201	0.1398
9	−0.7876	346.5367	0.2038	−2.1750	243.7024	0.1887	0.1033	172.7395	0.1584	0.9371	144.0843	0.1398
10	−0.7876	371.3379	0.2026	−2.1750	261.8643	0.1862	0.1033	184.8049	0.1580	0.9371	155.3776	0.1398
11	−0.7876	395.6874	0.2013	−2.1750	279.6207	0.1837	0.1033	196.5286	0.1576	0.9371	166.4608	0.1398
12	−0.7876	419.5583	0.2000	−2.1750	296.8402	0.1812	0.1033	207.8452	0.1572	0.9371	177.3057	0.1398
13	−0.7876	442.8024	0.1988	−2.1750	313.4236	0.1787	0.1033	218.7232	0.1568	0.9371	187.8984	0.1398
14	−0.7876	465.1326	0.1975	−2.1750	329.2852	0.1761	0.1033	229.1136	0.1564	0.9371	198.1163	0.1398
15	−0.7876	486.2071	0.1962	−2.1750	344.3674	0.1736	0.1033	238.9630	0.1560	0.9371	207.7776	0.1398
16	−0.7876	505.7398	0.1950	−2.1750	358.6387	0.1711	0.1033	248.2461	0.1556	0.9371	216.7982	0.1398
17	−0.7876	523.5836	0.1937	−2.1750	372.0983	0.1686	0.1033	256.9723	0.1552	0.9371	225.1710	0.1398
18	−0.7876	539.7165	0.1924	−2.1750	384.7434	0.1661	0.1033	265.1479	0.1547	0.9371	232.7857	0.1398
19	−0.7876	554.1764	0.1912	−2.1750	396.5833	0.1635	0.1033	272.7929	0.1543	0.9371	239.5830	0.1398
20	−0.7876	567.1207	0.1899	−2.1750	407.6567	0.1610	0.1033	279.9469	0.1539	0.9371	245.6109	0.1398
21	−0.7876	578.7817	0.1886	−2.1750	418.0442	0.1585	0.1033	286.6730	0.1535	0.9371	251.0496	0.1398
22	−0.7876	589.4770	0.1873	−2.1750	427.8971	0.1560	0.1033	293.0630	0.1531	0.9371	256.1671	0.1398
23	−0.7876	599.5300	0.1861	−2.1750	437.3887	0.1534	0.1033	299.2101	0.1527	0.9371	261.1406	0.1398
24	−0.7876	609.3164	0.1848	−2.1750	446.7229	0.1509	0.1033	305.2232	0.1523	0.9371	266.1367	0.1398
25	−0.7876	619.1593	0.1835	−2.1750	456.0570	0.1484	0.1033	311.2003	0.1518	0.9371	271.2515	0.1398
26	−0.7876	629.1747	0.1822	−2.1750	465.4360	0.1459	0.1033	317.2057	0.1514	0.9371	276.5640	0.1398
27	−0.7876	639.3019	0.1810	−2.1750	474.8382	0.1433	0.1033	323.2314	0.1510	0.9371	281.9813	0.1398
28	−0.7876	649.4860	0.1797	−2.1750	484.2530	0.1408	0.1033	329.2650	0.1506	0.9371	287.4341	0.1398
29	−0.7876	659.6776	0.1784	−2.1750	493.6694	0.1383	0.1033	335.2995	0.1502	0.9371	292.8916	0.1398
30	−0.7876	669.8691	0.1772	−2.1750	503.0858	0.1358	0.1033	341.3341	0.1498	0.9371	298.3491	0.1398

Values represent LMS parameters. *LMS* L = Lambda (skewness of the distribution), M = Mu (median), S = Sigma (variance)

z-score = [(X/M)L − 1]/(L*S), where X is the measured aortic area in mm^2^ and L, M, and S are the values interpolated for the child’s age; lower and upper limits correspond to a z-score of −2 and 2

^a^Aortic area measured at maximum distension of the aorta on cross sectional cine gradient echo images acquired perpendicular to the aortic axis (n = 30), ^b^measured above the diaphragm, ^c^ age in years

**Table 71:** Normal aortic diameters in children measured on a contrast enhanced 3D-MR angiography^a^ according to reference [101].

Site	Predicted diameter (mm)	SD of residuals (mm)
Aortic sinus	0.57 + 19.37*BSA^0.5^	2.38
Sinotubular junction	−0.03 + 16.91*BSA^0.5^	1.92
Ascending aorta	−1.33 + 18.6*BSA^0.5^	1.99
Proximal to the origin of the brachiocephalic artery	−3.38 + 20.07*BSA^0.5^	1.69
First transverse segment	−3.52 + 18.66*BSA^0.5^	1.63
Second transverse segment	−2.63 + 16.5*BSA^0.5^	1.31
Isthmic region	−3.37 + 16.52*BSA^0.5^	1.46
Descending aorta	−1.12 + 14.42*BSA^0.5^	1.64
Thoracoabdominal aorta at the level of the diaphragm	1.27 + 9.89*BSA^0.5^	1.34

*BSA* body surface area, *SD* standard deviation

^a^Shortest diameter measured on cross-sectional reformatted images (n = 53). Sites of measurement are shown in Fig. 9

z-score = (measured diameter − predicted diameter)/SD of residuals; lower and upper limits correspond to a z-score of −2 and 2

**Table 72:** Normal aortic area in children on phase contrast cine images^a^ according to reference [102].

Site	Predicted area (cm^2^)
Ascending aorta	−0.0386 + 2.913*BSA

*BSA* body surface area

^a^Cross-sectional area calculated based on measurement of the maximal external aortic diameter perpendicular to the vessel and perpendicular to the maximal diameter in systole 1-2 cm distal to the sinotubular junction on the magnitude image of a phase contrast cine sequence (n = 105)

## 16 Normal distensibility and pulse wave velocity of the thoracic aorta in adults

### 16.1 Influencing factors

**Gender:** Differences in aortic stiffness between men and women are subtle, with studies showing either no difference or slightly stiffer aortas in men [104], [105]. However, arterial stiffness in women progresses faster with age compared to men, possibly due to menopausal changes in women [105], [106].

**Fig. 12:**
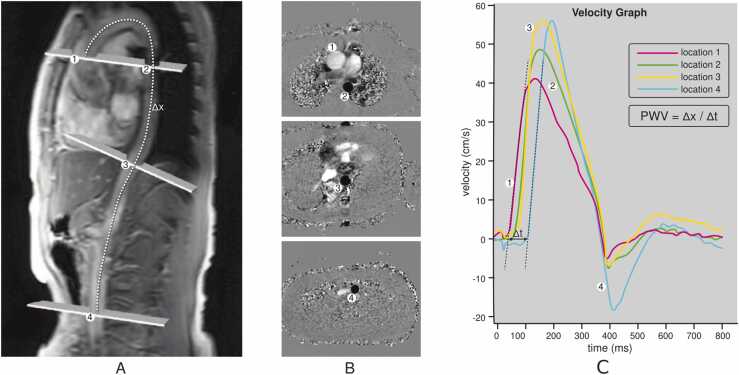
Measurement of aortic pulse wave velocity (PWV) according to reference [105]. Through plane flow-measurements at the level of the pulmonary trunk cutting across both the ascending and the proximal descending aorta, just below the diaphragm perpendicular to the descending aorta and just above the bifurcation of the abdominal aorta (A). Corresponding velocity encoded images (B). PWV is subsequently calculated from the distance along the aortic centerline between measurement locations (Δx) and the foot-to-foot transit time from the resulting velocity waveforms (Δt) (C).

**Table 73:** References. Normal distensibility and pulse wave velocity of the thoracic aorta in adults.

First author, year n, male:female Age range Study population	CMR acquisition	Post-processing (Fig. 12)
Kim, 2013 [104] 61:63 20-79 years Adult volunteers, Korea	• 1.5T; multi-slice through-plane phase contrast and cine imaging at 4 levels: ascending aorta, upper descending thoracic aorta at the level of the bifurcation of the pulmonary artery, lower descending aorta at the level of the diaphragm, and abdominal aorta just above the bifurcation.	• PWV = Δx/Δt; Δx = length of the centerline between the sites of measurement and Δt = transit time of the flow curves over this segment. Transit time was determined from the midpoint of the systolic up-slope on the flow versus time curve. PWV of the 3 segments as well as total PWV was calculated. • Distensibility: (Amax − Amin)/[Amin × (Pmax − Pmin)], where Amax and Amin represent the maximal and minimal cross-sectional area of the aorta, and Pmax and Pmin represent the systolic and diastolic blood pressure: calculated at the 4 levels from systolic and diastolic area measured by manual contouring.
Eikendal, 2016 [27] 57:61 25-35 years AMBITYON study, Netherlands	• 3T; multi-slice through-plane phase contrast at the level of the pulmonary trunk to obtain through-plane flow velocity in the ascending and proximal descending aorta and at the level of the dome of the liver to obtain the distal descending thoracic aorta.	• PWV. Transit time: intersection point of the constant horizontal diastolic flow and upslope of the systolic wave front, modeled by linear regression along the upslope (using values between 20% and 80%). Proximal, distal, and total PWV was calculated.
van Hout, 2021 [105] 259:138 45-65 years NEO study, Netherlands	• 1.5T, multi-slice through-plane phase contrast at the level of the pulmonary trunk perpendicular to the ascending/descending aorta, just below the diaphragm and just above the bifurcation.	• PWV. Transit time: intersection point of the constant horizontal diastolic flow and upslope of the systolic wave front, modeled by linear regression along the upslope (using values between 20% and 80%). Aortic arch, descending thoracic, abdominal, and total PWV was calculated. The total PWV was calculated as weighted mean by averaging the PWV of 4 segments (between the ascending, proximal and distal descending and abdominal aorta above the bifurcation, respectively) in proportion to their segment length.

*n* number of study subjects, *PWV* pulse wave velocity, *AMBITYON* Atherosclerosis Monitoring and Biomarker measurements in the Young, *NEO* Netherlands Epidemiology of Obesity

**Table 74:** Normal aortic distensibility (10^−^^3^ mm/Hg) by age for Korean men and women according to [104].

			Men (n = 61)		Women (n = 63)
Level	Age (years)	n	Mean ± SD	n	Mean ± SD
Ascending aorta^a^	20-29	12	5.6 ± 1.5	14	7.9 ± 3.4
	30-39	16	3.6 ± 1.4	12	6.5 ± 3.0
	40-49	11	3.5 ± 1.5	13	5.3 ± 1.2
	50-59	12	3.2 ± 1.6	13	3.6 ± 1.1
	60-69	10	2.1 ± 1.3	11	2.7 ± 1.0
Proximal descending aorta^a^	20-29	12	4.2 ± 0.9	14	6.0 ± 1.4
	30-39	16	3.8 ± 1.3	12	5.5 ± 1.9
	40-49	11	3.3 ± 0.6	13	4.2 ± 1.2
	50-59	12	2.9 ± 1.1	13	3.7 ± 1.3
	60-69	10	2.3 ± 0.9	11	3.1 ± 0.9
Distal descending aorta	20-29	12	5.8 ± 0.9	14	7.7 ± 1.7
	30-39	16	4.9 ± 1.7	12	6.6 ± 1.2
	40-49	11	5.0 ± 1.6	13	5.9 ± 1.3
	50-59	12	4.0 ± 2.0	13	4.0 ± 1.2
	60-69	10	3.2 ± 1.3	11	3.8 ± 1.4

*Data are means ± standard deviation. n* number of study subjects

^a^Measurements obtained at the level of the bifurcation of the pulmonary artery

**Table 75:** Normal aortic pulse wave velocity (m/s) by age in Korean adults according to [104].

Parameter	Age (years)	n	Median (5^th^-95^th^ percentile)
Regional PWV from the ascending to the upper descending thoracic aorta	20-29	26	3.7 (3.4-4.0)
	30-39	28	3.8 (3.5-6.0)
	40-49	24	4.3 (3.7-5.0)
	50-59	25	5.6 (5.4-7.2)
	60-69	21	9.0 (7.4-12.4)
Total PWV from the ascending aorta to the bifurcation	20-29	26	4.2 (4.0-4.5)
	30-39	28	4.5 (4.4-5.0)
	40-49	24	4.9 (4.6-5.3)
	50-59	25	5.8 (5.6-6.4)
	60-69	21	6.5 (6.2-7.7)

*Data are medians and 5th and 95th* percentile. *n* number of study subjects, *PWV* pulse wave velocity

**Table 76:** Normal aortic pulse wave velocity (m/s) by age in men and women according to [105].

		Men (n = 259)		Women (n = 138)	
Parameter	Age (years)	n	Median (10^th^-90^th^ percentile)	n	Median (10^th^-90^th^ percentile)
Regional PWV^a^	45-49	37	5.5 (4.9-6.8)	66	5.2 (4.6-6.1)
	50-54	31	5.8 (5.1-6.5)	71	5.6 (5.0-6.5)
	55-59	29	6.1 (5.3-7.8)	59	5.8 (5.0-7.0)
	60-64	41	6.8 (5.4-8.0)	63	6.8 (5.7-7.9)
Total PWV^b^	45-49	37	5.9 (4.7-6.9)	66	5.1 (4.0-7.5)
	50-54	31	5.5 (4.0-8.1)	71	5.8 (4.5-7.9)
	55-59	29	6.3 (4.7-7.4)	59	6.3 (4.7-8.9)
	60-64	41	6.1 (5.1-7.9)	63	6.0 (4.4-9.2)

*Data are medians and 10th and 90th precentile. n* number of study subjects, *PWV* pulse wave velocity

^a^From the ascending to the upper descending thoracic aorta, ^b^calculated as weighted mean by averaging the PWV of 4 segments (between the ascending, proximal and distal descending and abdominal aorta above the bifurcation, respectively) in proportion to their segment length

**Table 77:** Normal aortic pulse wave velocity (m/s) in young men and women (25-35 years) according to [27].

	Men (n = 57)	Women (n = 61)
Parameter	Median (10^th^-90^th^ percentile)	Median (10^th^-90^th^ percentile)
Regional PWV from the ascending to the upper descending thoracic aorta	4.6 (3.9-5.6)	4.5 (3.6-6.0)
Total PWV from the ascending to the distal descending thoracic aorta	4.4 (3.9-5.2)	4.5 (3.6-5.4)

*Data are medians and 10th and 90th precentile n* number of study subjects, *PWV* pulse wave velocity

**Table 78:** Normal total pulse wave velocity^a^ (m/s) by age in men and women according to [105].

	Men (n = 259)		Women (n = 138)	
Age (years)	n	Median (10^th^-90^th^ percentile)	n	Median (10^th^-90^th^ percentile)
45 to <50	37	5.5 (4.9-6.8)	66	5.2 (4.6-6.1)
50 to <55	31	5.8 (5.1-6.5)	71	5.6 (5.0-6.5)
55 to <60	29	6.1 (5.3-7.8)	59	5.8 (5.0-7.0)
60 to <65	41	6.8 (5.4-8.0)	63	6.8 (5.7-7.9)

*Data are medians and 10th and 90th percentile. n* number of study subjects

^a^Total pulse wave velocity from the ascending aorta to the aortic bifurcation

Reference [105] provided additional data

**Age:** Greater ascending aorta diameter and changes in aortic arch geometry with greater age were associated with increased regional stiffness of the aorta, especially of the ascending portion. The relationship of age with measures of aortic stiffness is non-linear and the decrease of aortic distensibility occurs particularly before the fifth decade of life [105], [107].

**Blood pressure:** Arterial stiffness is highly correlated with blood pressure, values represented in this article are normal values (e.g., normotensive participants). PWV in hypertensive individuals increases faster with aging as compared to normotensive individuals [105].

**Cardiovascular risk factors:** Besides hypertension, other cardiovascular risk factors such as smoking, diabetes, and hypercholesterolemia increase arterial stiffness [108].

**CMR acquisition:** 2D through-plane or in-plane phase-contrast or 4D flow PWV [109], [110]. 2D through-plane and in-plane phase-contrast PWV have been validated against invasive intra-aortic pressure measurements [110]. 4D flow is limited in temporal resolution, limiting its ability to measure high PWV values [109]. Acquisition of PWV over a short region of the aorta also requires higher temporal resolution and may be less accurate compared to PWV of the entire aorta. However, aortic arch PWV is more widely used as it has the advantage that its measurements can be obtained simultaneously on a single 2D acquisition at the level of the bifurcation of the pulmonary artery. Therefore, in the tables both arch and total aortic PWV are provided.

**Post processing:** Temporal shift: Time-to-peak is calculated from a single data point and therefore highly error prone. Time-to-foot, time-to-upstroke, and cross-correlation are reliable methods to determine temporal shift [109].

## 17 Normal distensibility and pulse wave velocity of the thoracic aorta in children

### 17.1 Influencing factors

**Gender:** Aortic distensibility and PWV did not vary by gender [103].

**Table 79:** References. Normal distensibility and pulse wave velocity of the thoracic aorta in children.

First author, year n, male:female Age range Study population	CMR acquisition	Post-processing (Fig. 12)
Voges, 2012 [103] 30:41 2-28 years Young volunteers + 5 children with neurologic disorders but free from cardiovascular disease, Germany	• 3.0T; gradient echo cine images for distensibility at the ascending aorta, transverse aortic arch, aortic isthmus, and descending aorta above the diaphragm and phase-contrast cine CMR at the level of the pulmonary artery bifurcation for aortic arch PWV.	• Distensibility at the 4 locations: (Amax − Amin)/[Amin × (Pmax − Pmin)], where Amax and Amin represent the maximal and minimal cross-sectional area of the aorta, and Pmax and Pmin represent the systolic and diastolic blood pressure. • PWV Δx/Δt, Δx = length of the centerline between the sites of measurement and Δt = transit time of the flow curves over this segment. Aortic arch PWV was calculated.

*n* number of study subjects, *PWV* pulse wave velocity

**Table 80:** LMS parameters to calculate z-scores for distensibility of the ascending aorta^a^ relative to age in children according to reference [103].

	Male (n = 30)			Female (n = 41)		
Age (years)	L	M	S	L	M	S
<1	−0.1879	12.3602	0.3680	−0.0721	12.7303	0.2388
1	−0.1879	11.9220	0.3680	−0.0721	12.5028	0.2396
2	−0.1879	11.4838	0.3680	−0.0721	12.2753	0.2403
3	−0.1879	11.0456	0.3680	−0.0721	12.0477	0.2411
4	−0.1879	10.6075	0.3680	−0.0721	11.8176	0.2419
5	−0.1879	10.1700	0.3680	−0.0721	11.5817	0.2427
6	−0.1879	9.7343	0.3680	−0.0721	11.3421	0.2435
7	−0.1879	9.2990	0.3680	−0.0721	11.1121	0.2443
8	−0.1879	8.8602	0.3680	−0.0721	10.9051	0.2451
9	−0.1879	8.4151	0.3680	−0.0721	10.7290	0.2459
10	−0.1879	7.9776	0.3680	−0.0721	10.5679	0.2467
11	−0.1879	7.5683	0.3680	−0.0721	10.3851	0.2474
12	−0.1879	7.2051	0.3680	−0.0721	10.1582	0.2482
13	−0.1879	6.9030	0.3680	−0.0721	9.8884	0.2490
14	−0.1879	6.6697	0.3680	−0.0721	9.5911	0.2498
15	−0.1879	6.5089	0.3680	−0.0721	9.2905	0.2506
16	−0.1879	6.4138	0.3680	−0.0721	9.0033	0.2514
17	−0.1879	6.3729	0.3680	−0.0721	8.7345	0.2522
18	−0.1879	6.3745	0.3680	−0.0721	8.4850	0.2529
19	−0.1879	6.4062	0.3680	−0.0721	8.2574	0.2537
20	−0.1879	6.4551	0.3680	−0.0721	8.0546	0.2545
21	−0.1879	6.5111	0.3680	−0.0721	7.8749	0.2553
22	−0.1879	6.5646	0.3680	−0.0721	7.7106	0.2561
23	−0.1879	6.6062	0.3680	−0.0721	7.5479	0.2569
24	−0.1879	6.6277	0.3680	−0.0721	7.3842	0.2577
25	−0.1879	6.6242	0.3680	−0.0721	7.2113	0.2584
26	−0.1879	6.5975	0.3680	−0.0721	7.0343	0.2592
27	−0.1879	6.5577	0.3680	−0.0721	6.8647	0.2600
28	−0.1879	6.5116	0.3680	−0.0721	6.6951	0.2608
29	−0.1879	6.4643	0.3680	−0.0721	6.5250	0.2616
30	−0.1879	6.4170	0.3680	−0.0721	6.3550	0.2624

*Values represent LMS parameters. n* number of study subjects, *LMS* L = Lambda (skewness of the distribution), M = Mu (median), S = Sigma (variance)

^a^Distensibility was calculated based on measurements of the aortic area at systole and diastole on cross-sectional cine gradient echo images obtained perpendicular to the axis of the ascending thoracic aorta

z-score = [(X/M)^L^ − 1]/(L*S), where X is the measured aortic distensibility in 10^−^^3^ mmHg^−^^1^ and L, M, and S are the values interpolated for the child’s age; lower and upper limits correspond to a z-score of −2 and 2

**Table 81:** LMS parameters to calculate z-scores for pulse wave velocity (PWV)^a^ relative to age in children according to reference [103].

	Male (n = 30)			Female (n = 41)		
Age (years)	L	M	S	L	M	S
<1	1.4844	3.4147	0.2122	−1.5196	2.7808	0.1468
1	1.4844	3.4367	0.2122	−1.5196	2.8144	0.1469
2	1.4844	3.4587	0.2122	−1.5196	2.8481	0.1469
3	1.4844	3.4808	0.2122	−1.5196	2.8817	0.1469
4	1.4844	3.5028	0.2122	−1.5196	2.9154	0.1470
5	1.4844	3.5248	0.2122	−1.5196	2.9490	0.1470
6	1.4844	3.5469	0.2122	−1.5196	2.9827	0.1470
7	1.4844	3.5689	0.2122	−1.5196	3.0163	0.1470
8	1.4844	3.5909	0.2122	−1.5196	3.0499	0.1471
9	1.4844	3.6129	0.2122	−1.5196	3.0836	0.1471
10	1.4844	3.6350	0.2122	−1.5196	3.1172	0.1471
11	1.4844	3.6570	0.2122	−1.5196	3.1509	0.1471
12	1.4844	3.6790	0.2122	−1.5196	3.1845	0.1472
13	1.4844	3.7011	0.2122	−1.5196	3.2182	0.1472
14	1.4844	3.7231	0.2122	−1.5196	3.2518	0.1472
15	1.4844	3.7451	0.2122	−1.5196	3.2855	0.1473
16	1.4844	3.7672	0.2122	−1.5196	3.3192	0.1473
17	1.4844	3.7892	0.2122	−1.5196	3.3528	0.1473
18	1.4844	3.8112	0.2122	−1.5196	3.3865	0.1473
19	1.4844	3.8333	0.2122	−1.5196	3.4201	0.1474
20	1.4844	3.8553	0.2122	−1.5196	3.4538	0.1474
21	1.4844	3.8773	0.2122	−1.5196	3.4875	0.1474
22	1.4844	3.8994	0.2122	−1.5196	3.5211	0.1475
23	1.4844	3.9214	0.2122	−1.5196	3.5548	0.1475
24	1.4844	3.9434	0.2122	−1.5196	3.5885	0.1475
25	1.4844	3.9655	0.2122	−1.5196	3.6221	0.1476
26	1.4844	3.9875	0.2122	−1.5196	3.6558	0.1476
27	1.4844	4.0096	0.2122	−1.5196	3.6895	0.1476
28	1.4844	4.0316	0.2122	−1.5196	3.7231	0.1476
29	1.4844	4.0536	0.2122	−1.5196	3.7568	0.1477
30	1.4844	4.0757	0.2122	−1.5196	3.7905	0.1477

*Values represent LMS parameters. n* number of study subjects, LMS, L = Lambda (skewness of the distribution), M = Mu (median), S = Sigma (variance)

^a^Pulse wave velocity calculated by phase contrast MR for the distance between the sinotubular junction and the proximal descending aorta. Transit time calculated from the midpoint of the systolic up-slope on the flow versus time curve (Fig. 12)

z-score = [(X/M)^L^ − 1]/(L*S), where X is the measured pulse wave velocity in m/s and L, M, and S are the values interpolated for the child’s age; lower and upper limits correspond to a z-score of −2 and 2

**Age and body composition:** Aortic distensibility decreases with age and correlates with height, body weight, and BSA. PWV increases with age [103].

**CMR acquisition/post-processing:** Similar as in adults.

## 18 Normal dimensions and distension of the pulmonary arteries in adults

### 18.1 Influencing factors

**Gender:** Areas and mean diameters of the pulmonary arteries (main and left and right pulmonary artery) are greater in men compared to women [98], [111]. Distension of the pulmonary arteries does not vary by gender [111].

**Fig. 13:**
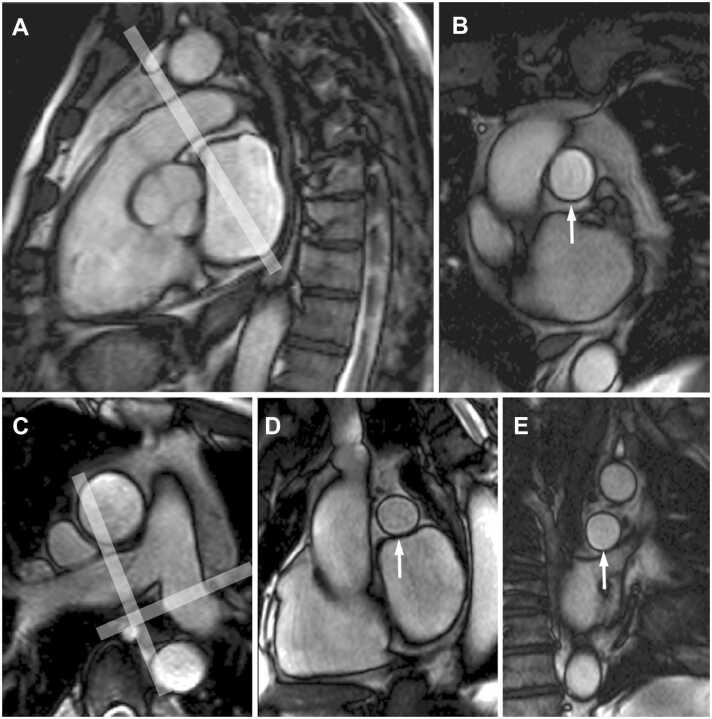
Measurement of the dimensions of the pulmonary arteries on balanced steady-state free precession images from reference [111]. Oblique sagittal image of the main pulmonary artery (A). The pale band in (A) shows the acquisition plane of the cross-sectional image of the main pulmonary artery in (B). Right and left pulmonary arteries on the scout image (C) with band indicating the location of cine acquisitions transecting the right (D) and left (E) pulmonary artery

**Table 82:** References. Normal dimensions and distension of the pulmonary arteries in adults.

First author, year n, male:female Age range Study population	CMR acquisition	Post-processing (Fig. 13)
Burman, 2016 [111] 60:60 20-79 years Volunteers, UK	• 1.5T, bSSFP • Cross-sectional image of the main pulmonary artery based on an oblique sagittal image of the right ventricular outflow tract/pulmonary trunk. • Cross-sectional images of the right and left pulmonary artery based on an axial image acquired at the level of the bifurcation of the main pulmonary artery.	• Luminal area and mean diameters measured at systole and diastole. • Diameter calculated as mean of the greatest diameter and the lesser diameter orthogonal to the greater diameter on cross sectional images. • Percent systolic distension calculated as [(maximum area − minimum area)*100/minimum area].
Li, 2021 [98] 100:100 20-70 years Volunteers, China	• 3T, HASTE • Axial slices with a thickness of 6-8 mm, no gap	• Luminal diameter

*n* number of study subjects, *bSSFP* balanced steady-state free precession, *HASTE* half Fourier single-shot turbo spin-echo

**Table 83:** Normal dimensions and distension of the pulmonary arteries in adult men and women by age measured on cross-sectional bSSFP images according to [111].

Vessel	Age^a^	n^b^	Systolic diameter (mm) Mean ± SD (LL-UL)		Diastolic diameter (mm) Mean ± SD (LL-UL)		Systolic area (cm^2^) Mean ± SD (LL-UL)		Diastolic area (cm^2^) Mean ± SD (LL-UL)		Distension (%) Mean ± SD (LL-UL)	
			Men	Women	Men	Women	Men	Women	Men	Women	Men	Women
MPA	20-29	10	28.3 ± 1.8	26.4 ± 3.3	22.4 ± 1.8	21.6 ± 2.9	6.3 ± 0.8	5.5 ± 1.4	3.9 ± 0.6	3.7 ± 1.0	61.5 ± 18.4	50.7 ± 19.6
	30-39	10	27.3 ± 2.7	25.5 ± 2.8	21.9 ± 1.8	20.4 ± 2.0	5.8 ± 1.2	5.1 ± 1.1	3.8 ± 0.6	3.3 ± 0.6	53.2 ± 14.9	54.8 ± 13.0
	40-49	10	28.2 ± 3.1	26.5 ± 1.6	23.5 ± 2.8	21.5 ± 1.6	6.2 ± 1.3	5.4 ± 0.6	4.4 ± 1.0	3.7 ± 0.5	41.4 ± 13.5	46.2 ± 9.6
	50-59	10	27.0 ± 2.7	24.4 ± 2.7	22.9 ± 3.0	20.7 ± 2.1	5.7 ± 1.2	4.7 ± 0.9	4.2 ± 1.0	3.4 ± 0.7	40.0 ± 12.6	38.5 ± 13.4
	60-69	10	26.5 ± 2.2	25.0 ± 2.7	23.3 ± 2.1	21.6 ± 2.0	5.5 ± 0.8	5.0 ± 1.0	4.3 ± 0.7	3.7 ± 0.7	29.6 ± 5.8	33.9 ± 10.4
	70-79	10	27.1 ± 2.7	24.0 ± 1.9	23.6 ± 2.6	21.4 ± 1.9	5.8 ± 1.2	4.6 ± 0.7	4.4 ± 0.9	3.6 ± 0.6	30.7 ± 11.6	26.8 ± 7.9
	*All*	*60*	*27.4* ± *2.6* *(22-33)*	*25.3* ± *2.6* *(20-31)*	*22.9* ± *2.4* *(18-28)*	*21.2* ± *2.1* *(17-25)*	*5.9* ± *1.1* *(3.7-8.1)*	*5.0* ± *1.0* *(3.0-7.0)*	*4.2* ± *0.8* *(2.6-5.8)*	*3.6* ± *0.7* *(2.2-5.0)*	*42.7* ± *17.2* *(8-77)*	*41.8* ± *15.7* *(10-73)*
RPA	20-29	10	18.6 ± 2.0	17.0 ± 1.5	14.3 ± 1.8	14.0 ± 2.0	2.8 ± 0.6	2.3 ± 0.4	1.7 ± 0.4	1.5 ± 0.3	69.1 ± 21.9	53.6 ± 18.0
	30-39	10	18.8 ± 2.1	16.5 ± 1.3	15.2 ± 1.5	13.3 ± 1.3	2.8 ± 0.6	2.2 ± 0.3	1.8 ± 0.4	1.4 ± 0.3	54.2 ± 15.9	55.7 ± 15.5
	40-49	10	20.3 ± 2.4	18.6 ± 2.0	16.4 ± 2.3	15.3 ± 1.8	3.3 ± 0.8	2.8 ± 0.6	2.2 ± 0.6	1.9 ± 0.5	53.1 ± 6.4	46.1 ± 7.5
	50-59	10	20.2 ± 2.4	16.8 ± 2.2	16.6 ± 2.1	14.0 ± 1.9	3.3 ± 0.8	2.3 ± 0.6	2.2 ± 0.6	1.6 ± 0.4	48.2 ± 11.6	44.9 ± 12.7
	60-69	10	20.2 ± 2.7	19.0 ± 2.4	17.1 ± 2.2	15.8 ± 1.7	3.3 ± 0.9	2.9 ± 0.7	2.4 ± 0.6	2.0 ± 0.4	37.2 ± 9.2	48.2 ± 17.6
	70-79	10	23.4 ± 3.6	18.8 ± 3.3	19.8 ± 3.5	15.9 ± 3.2	4.4 ± 1.2	2.9 ± 1.1	3.2 ± 1.0	2.1 ± 0.9	42.1 ± 14.0	40.4 ± 10.3
	*All*	*60*	*20.2* ± *2.9* *(14-26)*	*17.8* ± *2.4* *(13-23)*	*16.6* ± *2.8* *(11-22)*	*14.7* ± *2.2* *(10-19)*	*3.3* ± *1.0* *(1.3-5.3)*	*2.6* ± *0.7* *(1.2-4.0)*	*2.2* ± *0.8* *(0.6-3.8)*	*1.8* ± *0.6* *(0.6-3.0)*	*50.6* ± *16.9* *(17-84)*	*48.2* ± *14.5* *(19-77)*
LPA	20-29	10	18.8 ± 1.6	17.6 ± 1.2	15.8 ± 1.2	14.9 ± 1.4	2.8 ± 0.4	2.5 ± 0.3	2.0 ± 0.3	1.8 ± 0.3	39.6 ± 6.8	38.7 ± 7.9
	30-39	10	18.2 ± 1.9	16.9 ± 1.3	15.5 ± 1.7	14.7 ± 1.4	2.6 ± 0.5	2.3 ± 0.3	1.9 ± 0.4	1.8 ± 0.3	37.5 ± 11.0	33.2 ± 13.0
	40-49	10	19.8 ± 1.4	18.7 ± 1.4	16.7 ± 1.3	15.9 ± 1.1	3.2 ± 0.5	2.8 ± 0.4	2.2 ± 0.3	2.0 ± 0.3	42.2 ± 7.8	40.4 ± 9.9
	50-59	10	20.0 ± 2.0	18.1 ± 1.6	17.2 ± 1.7	15.9 ± 1.5	3.2 ± 0.6	2.7 ± 0.5	2.4 ± 0.5	2.1 ± 0.4	35.2 ± 11.3	30.3 ± 10.7
	60-69	10	21.0 ± 1.5	19.1 ± 2.1	18.4 ± 1.1	16.4 ± 1.7	3.5 ± 0.5	3.0 ± 0.6	2.7 ± 0.3	2.2 ± 0.5	30.2 ± 6.9	37.2 ± 9.6
	70-79	10	23.1 ± 2.7	20.1 ± 3.1	20.4 ± 3.4	17.6 ± 3.0	4.3 ± 1.0	3.3 ± 1.0	3.4 ± 1.1	2.5 ± 0.9	29.2 ± 11.0	31.2 ± 7.5
	*All*	*60*	*20.1* ± *2.4* *(15-25)*	*18.4* ± *2.1* *(14-23)*	*17.3* ± *2.5* *(12-22)*	*15.9* ± *2.0* *(12-20)*	*3.3* ± *0.8* *(1.7-4.9)*	*2.8* ± *0.6* *(1.6-4.0)*	*2.4* ± *0.7* *(1.0-3.8)*	*2.1* ± *0.5* *(1.1-3.1)*	*35.6* ± *10.1* *(15-56)*	*35.2* ± *10.3* *(15-56)*

*Data are means ± standard deviation. bSSFP* balanced steady state free precession, *n* number of study subjects, *SD* standard deviation, *LL* lower limit, *UL* upper limit, *MPA* main pulmonary artery, *RPA* right pulmonary artery, *LPA* left pulmonary artery

^a^In years, ^b^per gender

**Table 84:** Normal dimensions of the main pulmonary artery by age in adult Chinese measured on axial HASTE images according to [98].

			Men	Women
Parameter	Age	n	Mean ± SD (LL-UL)	Mean ± SD (LL-UL)
Diameter (mm)	20-30	20	22.5 ± 3.1	20.4 ± 2.2
	30-40	20	22.6 ± 2.4	20.3 ± 1.5
	40-50	20	23.1 ± 2.9	21.7 ± 1.6
	50-60	20	23.4 ± 2.9	22.9 ± 2.6
	60-70	20	24.1 ± 2.2	23.1 ± 2.8
	*All*	*100*	*23.1* ± *2.6* *(18-28)*	*21.7* ± *2.5* *(17-27)*
Diameter/BSA (mm/m^2^)	20-30	20	12.1 ± 1.6	13.1 ± 1.7
	30-40	20	12.3 ± 1.4	13.2 ± 1.0
	40-50	20	12.2 ± 0.9	13.4 ± 1.6
	50-60	20	12.4 ± 1.4	13.8 ± 1.5
	60-70	20	13.3 ± 1.4	14.2 ± 1.6
	*All*	*100*	*12.4* ± *1.4* *(10-15)*	*13.4* ± *1.6* *(10-17)*

*Data are means ± standard deviation. HASTE* half Fourier single-shot turbo spin-echo, *n* number of study subjects, *SD* standard deviation, *LL* lower limit, *UL* upper limit, *BSA* body surface area

**Age:** According to Burman et al., multivariable regression analysis showed a statistically significant greater systolic and diastolic area and diameter of the pulmonary arteries in men and women with greater age (except area and diameter of the main pulmonary artery at systole in men and at diastole in women) [111]. Also, Li et al. showed a larger diameter of the main pulmonary artery with greater age in men and women [98]. Distension of the pulmonary arteries was lower with higher age in men and women (except for the left pulmonary artery in women).

**BSA:** Distension of the pulmonary arteries in men and women was not related to body surface area. The majority of systolic and diastolic areas and diameters of the pulmonary arteries were greater with higher BSA in men and women [98], [111].

**Cardiac phase:** Areas and mean diameters of the pulmonary arteries are greater in systole compared to diastole [111].

**CMR acquisition:** Similar to the aorta, measurements of the pulmonary artery are expected to vary by the sequence type and might not be comparable [99].

## 19 Normal dimensions of the pulmonary arteries in children

### 19.1 Influencing factors

**Gender:** Kutty et al. did not find a significant gender difference of the size of the main pulmonary artery [102].

**Fig. 14:**
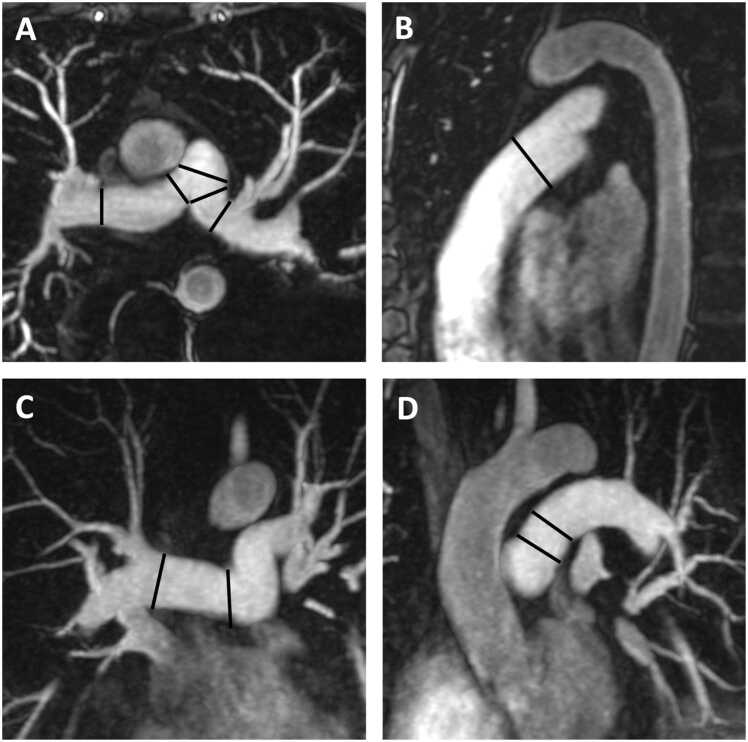
Measurement of the diameters of the pulmonary arteries according to reference [112]. Diameters were measured perpendicular to the vessel on maximum-intensity projection images. The diameters of the main pulmonary artery were obtained on an axial (A) and sagittal oblique (B) view and the diameters of the proximal and distal right and left pulmonary artery were obtained on axial (A) and right and left anterior oblique (paracoronal) views (C and D), respectively

**Table 85:** References. Normal dimensions and distension of the pulmonary arteries in children.

First author, year n, male:female Age range Study population	CMR acquisition	Post-processing
Knobel, 2011 [112] 41:28 2-20 years Patients with clinical indication for CMR, without cardiovascular disease	• 1.5T, contrast enhanced MRA (3D fast spoiled gradient echo sequence)	• Diameters were measured on maximum intensity projection images reconstructed perpendicular to the vessel (Fig. 14). Diameters of the main PA were obtained on an axial and sagittal oblique view and the diameters of the proximal and distal right and left PA were obtained on axial and right and left anterior oblique (paracoronal) views, respectively.
Kutty, 2012 [102] 55:50 4-20 years “normal controls” (not further specified)	• 1.5T, magnitude image of a through-plane free-breathing phase contrast sequence	• The cross-sectional area was calculated based on measurements of the maximal external PA diameter perpendicular to the vessel and perpendicular to the maximal diameter obtained midway between the level of the pulmonary valve and the bifurcation of the branch PAs.
Kutty, 2012 [102] (ref. only) 210:168 8-59 years Patients with repaired TOF	• See above	• See above
Voges, 2021 [113] (ref. only) 56:13 5-18 years Patients with TGA after arterial switch	• 3T, sagittal oblique bSSFP or cine GRE sequence, and 4D-MRA	• Systolic and diastolic diameters of the main PA were measured perpendicular to the vessel at the middle between the pulmonary valve and the PA bifurcation on sagittal oblique bSSFP or cine GRE images. • Systolic and diastolic diameters of the right and left PA were measured at three locations each, on cine bSSFP images acquired perpendicular to the vessels. • Cross-sectional area of the main PA and the PA branches was measured on 4D-MRA.

*n* number of study subjects, *MRA* magnetic resonance angiography, *PA* pulmonary artery, *TOF* tetralogy of Fallot, *bSSFP* balanced steady-state free-precession, *GRE* gradient echo, *TGA* transposition of the great arteries

**Table 86:** Normal diameters of the pulmonary arteries in children measured on a contrast enhanced MR-angiography according to reference [112].

Site	Predicted diameter (mm)	SD of residuals (mm)
Main pulmonary artery (axial)	4.85 + 13.43*BSA^0.5^	2.72
Main pulmonary artery (sagittal)	1.04 + 17.07*BSA^0.5^	2.01
Proximal right pulmonary artery (axial)	2.63 + 9.19*BSA^0.5^	1.65
Distal right pulmonary artery (axial)	3.9 + 6.25*BSA^0.5^	1.49
Proximal right pulmonary artery (RAO)	−0.69 + 14.3*BSA^0.5^	1.76
Distal right pulmonary artery (RAO)	−1.08 + 14.62*BSA^0.5^	1.6
Proximal left pulmonary artery (axial)	1.7 + 11.27*BSA^0.5^	1.37
Distal left pulmonary artery (axial)	−0.1 + 11.89*BSA^0.5^	1.51
Proximal left pulmonary artery (LAO)	−2.13 + 16.82*BSA^0.5^	1.88
Distal left pulmonary artery (LAO)	−2.08 + 13.64*BSA^0.5^	1.5

*SD* standard deviation, *BSA* body surface area, *RAO* right anterior oblique view (paracoronal, parallel to right pulmonary artery; Fig. 14), *LAO* left anterior oblique view (paracoronal, parallel to left pulmonary artery; Fig. 14), *MR* magnetic resonance

Fitting model for regression: diameter = a + b*BSA^0.5^

z-score = (measured diameter − predicted diameter)/SD of residuals; lower and upper limits correspond to a z-score of −2 and 2

**Table 87:** Normal pulmonary artery area measured on phase contrast cine images according to reference [102].

Site	Predicted area (cm^2^)
Main pulmonary artery	−0.2880 + 3.386*BSA

*BSA* body surface area

**Age:** The area of the main pulmonary artery was higher in older, compared to younger volunteers in the study by Kutty et al. [102].

**BSA:** Kutty et al. and Knobel et al. described a positive relationship between pulmonary artery diameter and BSA [102], [112].

**Congenital heart disease:** After repair of congenital heart disease, measurements of the pulmonary arteries might not be compared to normal values obtained in healthy children or adults. Therefore, dedicated reference ranges have been published for children and adults, respectively, after repair of tetralogy of Fallot (TOF) and after arterial switch of a transposition of the great arteries (TGA) [102], [113]. Studies are listed in Table 85. Reference ranges can be found in the original publications.

**CMR acquisition:** Measurements of the pulmonary artery are expected to vary by the sequence type and might not be comparable [99].

## 20 Normal native myocardial T1-relaxation time and extracellular volume (ECV) in adults

### 20.1 Influencing factors

**Gender:** Women had a longer (higher) native T1 compared to men across all ages in some studies [128], [147], but only up to the age of 45 years in another study [114]. Women had higher ECV than men [128].

**Fig. 15:**
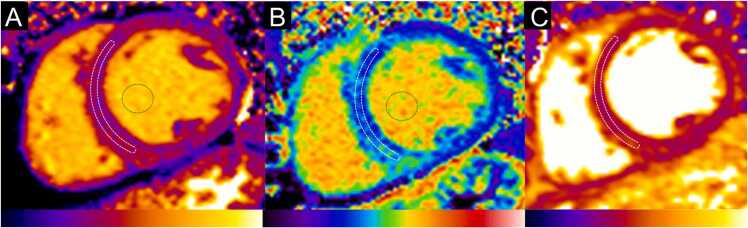
Example of measuring myocardial T1-relaxation time with a region of interest (ROI) in a MOLLI 5s(3s)3s native T1 map (A), a post-GBCA (gadolinium-based contrast agent) (B) T1 map and an extracellular volume map (C). According to the SCMR guidelines for post-processing in cardiovascular magnetic resonance, for global assessment of T1-relaxation time, the ROI was drawn in the septum of a mid-cavity short-axis map, avoiding inclusion of adjacent tissue [3]. A blood ROI was also drawn in the native T1 map and copied onto the post-GBCA map. *MOLLI* modified look-locker inversion-recovery, *SCMR* Society for Cardiovascular Magnetic Resonance

**Age:** Native T1 was found to shorten slightly with healthy aging using some sequences [128] but this was not replicated in other studies [147], [148] including a comprehensive meta-analysis [149]. Another study only found age-dependent T1 shortening in females but not in males [114]. ECV appeared to be consistently independent of age [128], [147], [148].

**Anthropometric measures:** ECV was positively associated with body mass index (BMI) [150].

**Substrates:** T1 and ECV were longer/higher in the presence of fibrosis, edema, or amyloid deposition and shorter/reduced in the presence of intramyocardial fat or iron deposition [151]. ECV was positively associated with myocardial steatosis [150].

**CMR acquisition:** Native and post-GBCA T1 times for blood and myocardium vary significantly by field strength [152], [153], [154]. ECV was independent of field strength in vivo provided the same contrast agent at equimolar doses was used [153] and this has also been independently confirmed in phantoms [155]. T1 values obtained from the same T1 mapping phantoms have also been shown to differ according to room temperature [155], magnet vendor, and software systems (including before and after major software upgrades [155]). Post-GBCA T1 is affected by the dose and relaxivity of the contrast agent used, rate of GBCA clearance, and the time between injection and measurement. Different T1 mapping sequences and within these, different prototypes, will have different susceptibilities to artifact/error and will consequently measure different native and post-GBCA T1 times [155], [156]. For MOLLI pulse sequences, the number of inversions, number of images following each inversion, the number of recovery beats between inversion pulses, and whether these are in seconds or heart beats, as well as the flip angle, have all been shown to affect the T1 measurements [149], [155].

**Post-processing:** T1 and ECV in myocardial segments free of late gadolinium enhancement (LGE) have been shown to be longer/higher in septal compared to non-septal segments [153], [157]. Conflicting data exist for myocardial T1 and ECV differences between systole and diastole with healthy volunteer studies showing shorter native T1, longer post-GBCA T1, and lower ECV at systole compared to diastole, but a larger disease cohort study showing no significant phase differences [153], [158], [159], [160]. It has been shown that T1-relaxation time varies by region of measurement (septal versus non septal) [153]. According to the SCMR guidelines for post-processing in CMR, for global assessment of T1-relaxation time a region of interest should be drawn in the septum of a mid-cavity short-axis map to reduce the impact of susceptibility artifacts from adjacent tissues [3]. Therefore, only values from publications in which the measurement was obtained in the midventricular septum in accordance with this recommendation were included in this review. The remaining publications, e.g., with measurements of T1-times of the entire left ventricular myocardium, are only listed in Table 88.

**Table 88:** References. Normal native myocardial T1-relaxation time and extracellular volume (ECV) in adults.

First author, year n, male:female Age range Study population	CMR acquisition	Parameter	Post-processing (Fig. 15)
Piechnik, 2013 [114] (ref. only) 169:173 11-69 years Volunteers, UK and Netherlands	• 1.5T, Siemens^a^ (Avanto) • ShMOLLI 5b(1b)1b(1b)1b • Basal, midventricular, and apical short-axis	• Native T1	• Global T1 calculated for the entire myocardium on all three short-axis slices.
Liu, 2014 [115] (ref. only) 38:54 37 (27-44) years^b^ Cohort study in African-Americans, USA	• 3T, Siemens^a^ (Avanto) ShMOLLI 5b(1b)1b(1b)1b • Four-chamber view	• Native T1	• T1 measured in the interventricular septum of a four-chamber view
Reiter, 2014 [116] 20:20 20-35 years Volunteers, Austria	• 1.5T, Siemens^a^ (Espree) • MOLLI 5b(4b)2b • Basal, midventricular, and apical short-axis • Systole and diastole	• Native T1	• T1 measured per segment for all 16 segments in systole and diastole with and without blood pool normalization • Global T1 calculated as the average of all 16 segments and average T1 per slice.
aus dem Siepen, 2015 [117] (ref. only) 37:19 (52 ± 9)^c^ years Volunteers, Germany	• 1.5T, Philips^d^ (Achieva) • MOLLI 3b(3s)3b(3s)5b • Midventricular short-axis	• Native T1 • ECV	• T1 calculated as the mean T1 of all segments of the midventricular short-axis slice.
Edwards, 2015 [118] 24:19 57 ± 10 years Volunteers, UK	• 1.5T, Siemens^a^ (Avanto) • MOLLI 3b(3s)3b(3s)5b • Basal and midventricular short-axis	• Native T1 • ECV	• T1 calculated as the average of two measurements at the basal and midventricular interventricular septum.
Goebel, 2016 [119] (ref. only) 31:23 18-63 years Subjects referred for “preventive check-up” with normal CMR and no clinical cardiac abnormality, Germany	• 1.5T, Siemens^a^ (Avanto) • MOLLI 5b(3s)3b • Midventricular short-axis	• Native T1	• T1 measured for the entire midventricular short-axis slice.
Gormeli, 2016 [120] 26:15 (24 ± 4)^c^ years Volunteers, Turkey	• 3T, Siemens^a^ (Skyra) • MOLLI 5b(3s)3b • Mid ventricular short-axis	• Native T1	• T1 measured for the entire myocardium of a midventricular short-axis slice and the interventricular septum.
Hinojar, 2016 [121] 9:37 42 ± 15 years Control subjects (country/ethnicity not specified)	• 3T, Philips^d^ (Achieva) • MOLLI 3b(3s)3b(3s)5b • Mid ventricular short-axis	• Native T1	• T1 measured in the interventricular septum at midventricular short axis
Rauhalammi, 2016 [122] 43:41 (45 ± 18)^c^ years Volunteers, UK	• 1.5T, Siemens^a^ (Avanto) • 3T, Siemen (Verio) • MOLLI 3b(3s)3b(3s)5b • Basal, midventricular and apical short-axis	• Native T1	• T1 measured per segment for all 16 segments. • Average T1 of all interventricular septal and lateral segments calculated.
Costello, 2017 [123] 29:28 (48 ± 15)^c^ years Volunteers, Australia	• 3T, Siemens^a^ (Prisma) • ShMOLLI 5b(1b)1b(1b)1b • SASHA • Midventricular short axis	• Native T1 • ECV	• T1 measured for the entire myocardium of a midventricular short-axis slice and the interventricular septum.
Avitzur, 2018 [124] (ref. only) 83:57 (54 ± 9)^c^ years Volunteers, Canada	• 3T, Siemens^a^ (Prisma) • ShMOLLI 5b(1b)1b(1b)1b • Basal and midventricular short-axis	• Native T1	• T1 measured per segment for all 12 segments of a basal and midventricular short-axis slice.
Doerner, 2018 [125] 30:20 (39 ± 17)^c^ years Volunteers, Germany	• 1.5T, Philips^d^ (Ingenia) • MOLLI 3b(3s)3b(3s)5b • Midventricular short-axis	• Native T1 • ECV	• T1 measured in the interventricular septum at midventricular short-axis.
Guo, 2018 [126] (ref. only) 18:32 (36 ± 16)^c^ years Volunteers, China	• 3T, Philips^d^ (Ingenia) • MOLLI 5s(3s)3s • Basal, midventricular, and apical short-axis	• Native T1 • ECV	• Global T1 calculated for the entire myocardium of all three short-axis slices.
Ridouani, 2018 [127] (ref. only) 20:20 (40 ± 12)^c^ years Healthy controls, France	• 1.5T, Siemens^a^ (Avanto) • MOLLI 3b(3s)3b(3s)5b • Midventricular short-axis and four-chamber view	• Native T1	• T1 calculated as the average T1 of the entire myocardium of a midventricular short-axis slice and a 4Chview 4ch-view.
Rosmini, 2018 [128] 49:45 20-76 years Volunteers, UK	• 1.5T, Siemens^a^ (Avanto) • MOLLI 5s(3s)3s (native) • MOLLI 4s(1s)3s(1s)2s (post GBCA) • ShMOLLI 5b(1b)1b(1b)1b • SASHA • Midventricular short-axis	• Native T1 • ECV	• T1 measured in the interventricular septum at midventricular short-axis.
Shang, 2018 [129] (ref. only) 45 (total) -^e^ Healthy controls, China	• 3T, Siemens^a^ (Trio) • MOLLI 5b(3b)3b (native) • MOLLI 4b(1b)3b(1b)2b (post GBCA) • Basal, midventricular, and apical short-axis	• ECV	• ECV calculated for all 16 segments. • Average ECV calculated for the entire midventricular short-axis slice and the interventricular septum.
Yang, 2018 [130] (ref. only) 18:26 (33 ± 16)^b^ years Volunteers, China	• 3T, Siemens^a^ (Tim Trio) • MOLLI 5b(3b)3b(3b)3b (native) • MOLLI 4b(1b)3b(1b)2b (post GBCA)	• Native T1 • ECV	• Global T1 calculated for the entire myocardium of all three short-axis slices. • Interventricular septal T1 calculated (NS if all septal segments or midventricular septum).
Granitz, 2019 [131] (ref. only) 26:32 (male: 42 ± 13 years, female: 40 ± 14 years)^c^ Volunteers, Austria	• 1.5T Philips^d^ (Ingenia) • 3T Philips^d^ (Achieva) • MOLLI 5b(3s)3b • Basal, midventricular, and apical short-axis	• Native T1	• T1 measured per segment for all 16 segments. • Global T1 calculated as the average of all segments.
Imran, 2019 [132] 26:25 (46 ± 14)^c^ years Healthy controls, Australia	• 1.5T, Philips^d^ (Achieva) • MOLLI 3b(2s)5b • Midventricular short-axis	• Native T1	• T1 measured for the entire myocardium of a midventricular short-axis slice and the interventricular septum.
Lehmonen, 2019 [133] (ref. only) 46 (total) (46 ± 9)^c^ years Healthy controls, Finland	• 1.5T, Siemens^a^ (Avanto) • ShMOLLI 5b(1b)1b(1b)1b • Basal, midventricular, and apical short-axis	• Native T1	• T1 measured per segment for all 16 segments (results not shown). • Global T1 calculated as the average of all segments.
Emrich, 2020 [134] (ref. only) 32:26 (50 ± 15)^c^ years Volunteers, Germany	• 1.5T Siemens^a^ (Avanto) • MOLLI 5s(3s)3s • Basal, midventricular, and apical short-axis	• Native T1	• T1 measured per segment for all 16 segments. • Global T1 calculated as the average of all segments.
Puyol-Anton, 2020 [135] 2299:1849 (61 ± 7)^c^ years UK Biobank, UK	• 1.5T Siemens^a^ (Aera) • ShMOLLI 5b(1b)1b(1b)1b • Mid-ventricular Midventricular short-axis	• Native T1	• T1 measured for the entire myocardium of a midventricular short-axis slice, the interventricular septum, and the left ventricular free wall.
Boettcher, 2021 [136] (ref. only) 21:29 (39 ± 14)^c^ years Volunteers, Germany	• 1.5T Siemens^a^ (Avanto) • MOLLI 5s(3s)3s • Basal, midventricular, and apical short-axis	• Native T1	• T1 measured per segment for all 16 segments. • Global T1 calculated as the average of all segments.
Graham-Brown, 2021 [137] (ref. only) 40 (total) -^e^ Volunteers, UK	• 3T, Siemens^a^ (Skyra) • MOLLI 5s(3s)3s • Midventricular short-axis	• Native T1	• T1 measured for the entire myocardium of a midventricular short-axis slice.
Lin, 2021 [138] (ref. only) 21:21 (44 ± 10)^c^ years Volunteers, China	• 3T, Siemens^a^ (Verio) • MOLLI 5s(3s)3s • Midventricular short-axis	• Native T1	• Region of measurement not specified.
Meloni, 2021 [18] 50:50 20-70 years Volunteers, Italy	• 1.5T, GE^f^ (Signa Artist) • MOLLI 3b(3s)3b(3s)5b • Basal, midventricular, and apical short-axis	• Native T1	• T1 measured per segment for all 16 segments. • Global T1 calculated as the average of all segments.
Cavus, 2022 [139] 50:79 59 (52-68)^b^ years HCHS, Germany	• 3T, Siemens^a^ (Skyra) • MOLLI 5b(3b)3b (native) • MOLLI 4b(1b)3b(1b)2b (post GBCA) • Basal, midventricular, and apical short-axis	• Native T1 • ECV	• T1 measured in the interventricular septum at midventricular short-axis.
Kersten, 2022 [140] (ref. only) 49:26 18-70 years Caucasian volunteers, Germany	• 1.5T Siemens^a^ (Aera) • MOLLI 5b(3s)3b • Midventricular short-axis	• Native T1	• T1 measured for the entire myocardium of a midventricular short-axis slice.
Li, 2022 [141] (ref. only) 21:59 (44 ± 15)^c^ years Volunteers, China	• 3T, Philips^d^ (Achieva) • MOLLI 5b(3b)3b • 1-3 short-axis slices	• Native T1	• Region of measurement not specified.
Shaw, 2022 [142] 36:14 18-60 years Volunteers, India	• 1.5T, Siemens^a^ (Aera) • MOLLI 5b(3b)5b(3b)5b(3b) • Basal, midventricular, and apical short-axis at two different flip angles (35° and 50°)	• Native T1	• T1 measured per segment for all 16 segments.
Snel, 2022 [143] (ref. only) 44:47 18-45 years Volunteers, Netherlands	• 3T, Siemens^a^ (Prisma) • MOLLI 5s(3s)3s (native) • 4s(1s)3s(1s)2s (post-GBCA) • Basal, midventricular, and apical short-axis	• Native T1 • ECV	• T1 measured per segment for all 16 segments (results not shown). • Global T1 calculated as the average of all segments.
Suh, 2022 [144] 69 (total) 20-79 years Volunteers, Korea	• 3T, Siemens^a^ (Prisma) • MOLLI 5s(3s)3s (native) • MOLLI 4s(1s)3s(1s)2s (post-GBCA) • Basal, midventricular, and apical short-axis	• Native T1 • ECV	• Global T1 calculated for the entire myocardium of all three short axis slices. • T1 measured in the interventricular septum at midventricular short-axis.
Xu, 2023 [145] (ref. only) 515:500 19-87 years Volunteers, China	• 3T, Siemens^a^ (Prisma or Skyra) • MOLLI 5s(3s)3s • Basal, midventricular, and apical short-axis, 2-, 3-, and 4-chamber view	• Native T1	• T1 measured per segment for all 16 segments. • T1 calculated per slice. • Interventricular septal T1 calculated (not specified if all septal segments or midventricular septum).
Yamagata, 2023 [146] 24:27 19-59 years Volunteers, Malta	• 3T, Siemens^a^ (Vida) • MOLLI 5s(3s)3s, 3 short-axis slices • Basal, midventricular, and apical short-axis	• Native T1	• T1 measured in the interventricular septum at midventricular short-axis.

*n* number of study subjects, *ref.* reference, *T* Tesla, *MOLLI* modified look-locker inversion-recovery, *ShMOLLI* shortened modified look-locker inversion-recovery, *ECV* extracellular volume fraction, *s* seconds, *b* beats, *GBCA* gadolinium-based contrast agent, *SASHA* saturation recovery single-shot acquisition, *NS* not specified, *HCHS* Hamburg City Health study

^a^Siemens Healthineers AG, Erlangen, Germany; ^b^median (interquartile range) (complete age range not provided in original publication); ^c^mean ± SD (age range not provided in original publication); ^d^Koninklijke Philips N.V., Amsterdam, The Netherlands; ^e^not provided in original publication; ^f^GE Healthcare Technologies, Chicago, Illinois, USA

Arrhythmias may interfere with ECG gating that is needed for robust T1 mapping such that longer native myocardial T1 times have been reported in patients with atrial fibrillation [161]. The type of motion correction algorithm used can significantly affect the quality of derived T1 and ECV maps [162]. For ECV map generation, even small differences in respiratory position or changes in patient position due to movement will cause significant misregistration of the native and post-GBCA images [162]. The method used for blood pool segmentation (manual contour versus automatic segmentation mask) does not appear to significantly affect T1-estimates, but when applying manual contours, the size and location of the blood pool region of interest (ROI), whether in the left as opposed to the right ventricular lumen and in terms of avoiding papillary muscles and subvalvular structures, does affect T1-estimates. ECV maps are contingent on the establishment of a dynamic equilibrium so the timing of post-GBCA T1 maps at least 10 min after native mapping is key and earlier acquisitions risk confounding ECV results [163]. ECV measurements may be affected by the nonstandardized acquisition of blood samples for hematocrit (Hct) so collecting these systematically after the CMR examination, when the patient is still in the supine position, has been shown to increase the precision of ECV measurements [164]. Equally significant ECV errors are known to occur in patients with severe anemia [165].

Since it has been shown that the native T1 time of blood is closely correlated with the hematocrit, in clinical situations in which an additional blood sample to determine the hematocrit would be too laborious, the hematocrit can be derived from the native T1 time of the blood for calculation of a synthetic ECV. However, quantification of T1-relaxation time depends on several factors, including the field strength and other scanner properties and sequence characteristics. Differences exist between published regression formulas for synthetic ECV calculation [166], so that scanner and pulse sequence specific should be used [167].

The following tables with reference ranges for T1 time and ECV are for guidance only. Due to the various influencing factors described above, site-specific reference ranges must be established in accordance with the current SCMR consensus guidelines [167].

**Table 89:** Normal native myocardial T1-relaxation time (ms) in adults.^a^

Reference(s)	FS	Vendor	Sequence	n	Mean ± SD	LL-UL
[18]	1.5	GE^b^	MOLLI 3b(3s)3b(3s)5b	100	1027 ± 36^c^	956-1098
[132]	1.5	Philips^d^	MOLLI 3b(2s)5b	51	974 ± 45	884-1064
[125]	1.5	Philips^d^	MOLLI 3b(3s)3b(3s)5b	50	967 ± 28	911-1023
[116]	1.5	Siemens^e^	MOLLI 5b(4b)2b	40	996 ± 32^c^	933-1059
[128]	1.5	Siemens^e^	MOLLI 5s(3s)3s	94	1024 ± 39	946-1102
[142]	1.5	Siemens^e^	MOLLI 5b(3b)5b(3b)5b(3b)	50	928 ± 25^c^	879-977
[118], [122]	1.5	Siemens^e^	MOLLI 3b(3s)3b(3s)5b	127	952 ± 35	883-1020
[128], [135]	1.5	Siemens^e^	ShMOLLI 5b(1b)1b(1b)1b	4242	954 ± 41	874-1034
[128]	1.5	Siemens^e^	SASHA	94	1144 ± 45	1054-1234
[121]	3	Philips^d^	MOLLI 3b(3s)3b(3s)5b	46	1057 ± 23	1011-1103
[144], [146]	3	Siemens^e^	MOLLI 5s(3s)3s	120	1214 ± 39	1138-1289
[120]	3	Siemens^e^	MOLLI 5b(3s)3b	41	1180 ± 27	1126-1234
[139]	3	Siemens^e^	MOLLI 5b(3b)3b	129	1182 (1162-1200)^f^	
[123]	3	Siemens^e^	ShMOLLI 5b(1b)1b(1b)1b	57	1125 ± 45	1035-1215
[123]	3	Siemens^e^	SASHA	57	1494 ± 43	1408-1580

*Data are means ± standard deviation or median and interquartile range, as indicated. FS* field strength in Tesla, *n* number of subjects, *SD* standard deviation, *LL* lower limit, *UL* upper limit, *MOLLI* modified look-locker inversion-recovery, *ShMOLLI* shortened modified look-locker inversion-recovery, *SASHA* saturation recovery single-shot acquisition

^a^Measured in the interventricular septum of a mid-ventricular short-axis slice (Fig. 15); ^b^ GE Healthcare Technologies, Chicago, Illinois, USA; ^c^calculated as the average of segments 8 and 9; ^d^Koninklijke Philips N.V., Amsterdam, The Netherlands; ^e^Siemens Healthineers AG, Erlangen, Germany; ^f^median, first and third quartile (mean ± SD not provided in original publication)

**Table 90:** Normal ECV (extracellular volume fraction in %) in adults.^a^

Reference (s)	FS	Vendor	Sequence	n	Mean ± SD	LL-UL
[125]	1.5	Philips^b^	MOLLI 3b(3s)3b(3s)5b	50	27.7 ± 5.9	15.9-39.5
[128]	1.5	Siemens^c^	MOLLI (native) 5s(3s)3s MOLLI (post GBCA) 4s(1s)3s(1s)2s	94	27.3 ± 2.7	21.9-32.7
[118]	1.5	Siemens^c^	MOLLI 3b(3s)3b(3s)5b	43	25.0 ± 3.0	19.0-31.0
[128]	1.5	Siemens^c^	ShMOLLI 5b(1b)1b(1b)1b	94	28.4 ± 3.0	22.4-34.4
[128]	1.5	Siemens^c^	SASHA	94	24.1 ± 2.9	18.3-29.9
[144]	3	Siemens^c^	MOLLI (native) 5s(3s)3s MOLLI (post GBCA) 4s(1s)3s(1s)2s	69	27.3 ± 2.4	22.5-32.1
[139]	3	Siemens^c^	MOLLI (native) 5b(3b)3b MOLLI (post GBCA) 4b(1b)3b(1b)2b	129	28 (26-29)^d^	
[123]	3	Siemens^c^	ShMOLLI 5b(1b)1b(1b)1b	57	24.6 ± 2.5	19.6-29.6
[123]	3	Siemens^c^	SASHA	57	19.8 ± 1.8	16.2-23.4

*Data are means ± standard deviation or median and interquartile range, as indicated. FS* field strength in Tesla, *n* number of subjects, *LL* lower limit, *UL* upper limit, *MOLLI* modified look-locker inversion-recovery, *GBCA* gadolinium-based contrast agent, *ShMOLLI* shortened modified look-locker inversion-recovery, *SASHA* saturation recovery single-shot acquisition

^a^For ECV calculation, T1 measured in the interventricular septum of a mid-ventricular short-axis slice (Fig. 15); ^b^Koninklijke Philips N.V., Amsterdam, The Netherlands; ^c^Siemens Healthineers AG, Erlangen, Germany; ^d^median, first, and third quartile (mean ± SD not provided in original publication)

## 21 Normal native myocardial T1-relaxation time and extracellular volume fraction (ECV) in children

### 21.1 Influencing factors

**Gender:** Female children had a higher native T1 than males in some studies [54] but not in others [173] and there was no consistent sex difference in ECV [173], [174].

**Table 91:** References. Normal native myocardial T1-relaxation time and extracellular volume (ECV) in children.

First author, year n, male:female Age range Study population	CMR acquisition	Parameter	Post-processing (Fig. 15)
Barczuk-Falęcka, 2020 [168] 18:20 9-18 years Volunteers, Poland	• 3T, Siemens^a^ (Skyra) • MOLLI 5s(3s)3s • Basal, midventricular, and apical short-axis	• Native T1	• T1 measured in the interventricular septum at midventricular short‐axis
Burkhardt, 2020 [169] 14:20 8-18 years Volunteers, Switzerland	• 1.5T, GE^b^ (Discovery MR 450) • MOLLI 3b(3s)3b(3s)5b • SmartT1map (single point saturation recovery)	• Native T1	• T1 measured for the entire myocardium of each slice and for the interventricular septum of each slice.
Wang, 2020 [170] 9:6 6-13 years Volunteers, China	• 3T, Siemens^a^ (Skyra) • MOLLI 3s(3s)5s • horizontal long-axis and basal, midventricular, and apical short-axis	• Native T1 • ECV	• Global T1 calculated for the entire myocardium of all three short-axis slices.
Alsaied, 2021 [171] (ref. only) 87:15 5-19 years Patients with pectus excavatum but no cardiac abnormalities	• 1.5T, Philips^c^ (Ingenia) • MOLLI 5s(3s)3s • Midventricular short-axis	• Native T1	• T1 measured for the entire midventricular short-axis slice
Mawad, 2021 [172] 25:21 (14.2 ± 2.4)^d^ years Patients with a family history of cardiomyopathy or for coronary artery imaging in non-specific chest pain and negative entire work-up	• 1.5T, Siemens^a^ (Avanto) • MOLLI 5b(3s)3b • Midventricular short-axis	• Native T1	• T1 measured for the entire myocardium of a midventricular short-axis slice, the interventricular septum and the left ventricular free wall.
Real, 2023 [54] 58:61 15-18 years Participants of the EnIGMA project without evidence or history of cardiovascular disease, Spain	• 3T, Philips^c^ (Elition X) • MOLLI 5s(3s)3s • Midventricular short-axis	• Native T1	• T1 measured per segment for all 6 segments. • T1 calculated as the average of all 6 segments and septal T1 as the average of segments 8 and 9.

*n* number of study subjects, *T* Tesla, *MOLLI* modified look-locker inversion-recovery, *EnIGMA* early imaging markers of unhealthy lifestyles in adolescents

^a^Siemens Healthineers AG, Erlangen, Germany; ^b^GE Healthcare Technologies, Chicago, Illinois, USA; ^c^Koninklijke Philips N.V., Amsterdam, The Netherlands; ^d^mean ± SD (age range not provided in original publication)

**Table 92:** Normal native myocardial T1-relaxation time (ms) in children.^a^

Reference	FS	Vendor	Technique	n	Mean ± SD	LL-UL
[169]	1.5T	GE^b^	MOLLI 3b(3s)3b(3s)5b	32	1017 ± 48	921-1113
[169]	1.5T	GE^b^	SmartT1map (single point saturation recovery)	34	1191 ± 62	1067-1315
[172]	1.5T	Siemens^c^	MOLLI 5b(3s)3b	46	1005 ± 40	925-1085
[54]	3T	Philips^d^	MOLLI 5s(3s)3s	119	1241 ± 35	1171-1311
[168]	3T	Siemens^c^	MOLLI 5s(3s)3s	38	1223 ± 29	1165-1281

*Data are means ± standard deviation. T* Tesla, *n* number of subjects, *SD* standard deviation, *LL* lower limit, *UL* upper limit, *MOLLI* modified look-locker inversion-recovery

^a^Measured with a region of interest in the interventricular septum of a mid-ventricular short-axis slice (15; ^b^GE Healthcare Technologies, Chicago, Illinois, USA; ^c^Siemens Healthineers AG, Erlangen, Germany; ^d^Koninklijke Philips N.V., Amsterdam, The Netherlands

**Table 93:** Normal ECV (extracellular volume fraction in %) in children.^a^

Reference	FS	Vendor	Technique	n	Mean ± SD	LL-UL
[170]	3T	Siemens^b^	MOLLI 3s(3s)5s	15	27.0 ± 0.6	25.8- 28.2

*Data are means ± standard deviation. T* Tesla, *n* number of subjects, *SD* standard deviation, *LL* lower limit, *UL* upper limit, *MOLLI* modified look-locker inversion-recovery

^a^Global T1 calculated for the entire myocardium of three short-axis slices; ^b^Siemens Healthineers AG, Erlangen, Germany

**Age:** ECV in children showed a weak positive correlation with age [173].

**Anthropometric measures:** In children, ECV showed a significant positive association with BMI [175] and a weak positive correlation with BSA [173].

**Substrates:** (Please see corresponding section for adults.).

**CMR-acquisition:** (Please see corresponding section for adults.).

**Post-processing**: T1 [173], [176] and ECV [173] in myocardial segments of children free of late gadolinium enhancement (LGE) have been shown to be longer in septal compared to non-septal segments. (Please also see corresponding section for adults.).

## 22 Normal myocardial T2-relaxation time in adults

### 22.1 Influencing factors

**Gender:** One study using gradient spin-echo (GraSE) demonstrated a slightly longer (higher) myocardial T2 in females as compared to males at 1.5T but no difference at 3T [131]; another study using T2-prepared fast-low-angle shot (FLASH) found longer T2 in females compared to males at 3T [139]; while another study using T2-prepared balanced steady-state free precession (bSSFP) showed no significant sex differences [179].

**Age:** T2 has not been found to vary with age across several studies [131], [179] but in one study age was associated with slightly longer T2 [180].

**Anthropometric measures:** T2 showed an independent positive association with body mass index (BMI) [139].

**Substrates:** T2 is influenced by the native T1 time of the myocardium being sampled, particularly if using a T2-prepared bSSFP-based T2 mapping sequence which has an inherent T1 bias related to the variable signal decay after the preparatory phase [181]. For example, when the myocardial T1 is short (e.g., Fabry’s disease), T2 times tend to be overestimated, while in amyloid with long native T1 times, T2 tends to be underestimated [182].

**CMR-acquisition:** T2 times for myocardium vary significantly across field strengths with shorter T2 at 3T compared to 1.5T both in vivo [131] and in phantoms [183]. T2 assessments exhibit greater temperature dependency than T1 in phantoms [155]. T2 measurements are influenced by magnet vendor and T2 mapping sequence prototypes [184]. Other factors that may influence T2 include scanner version, bore size, surface coils, intrinsic physical tissue factors as well as operator experience [185].

**Post-processing:** T2 times in myocardial segments free of late gadolinium enhancement (LGE) have been shown to be longer in nonseptal compared to septal segments [186] and longer in apical compared to basal and mid-ventricular slices [187]. According to the SCMR guidelines for post-processing in CMR, for global assessment of T2-relaxation time, a region of interest should be drawn in the septum of a mid-cavity short-axis map to reduce the impact of susceptibility artifacts from adjacent tissues [3]. Therefore, only values from publications in which the measurement was obtained in the midventricular septum in accordance with this recommendation were included in this review. The remaining publications, e.g., with measurements of T2-times of the entire left ventricular myocardium, are only listed in Table 94. Additionally, apical T2 times were significantly shorter (lower) in systole than in diastole [187]. In phantoms, arrhythmia and heart rate did not significantly impact myocardial T2 times using a T2-prepared bSSFP sequence [188].

**Table 94:** References. Normal myocardial T2-relaxation time in adults.

First author, year n, male:female Age range Study population	CMR acquisition	Post-processing
Wassmuth, 2013[177] (ref. only) 60:13 20-70 years Volunteers, Germany	• 1.5T, Siemens^a^ (Avanto) • T2-prepared SSFP • T2-prepared FLASH • Midventricular short-axis and 4-chamber view	• T2 calculated for the entire myocardium of a midventricular short-axis slice and a 4-chamber view.
Hinojar, 2016 [121] 9:37 (42 ± 15)^b^ years Control subjects (country/ethnicity not specified)	• 3T, Philips^c^ (Achieva) • T2 GraSE • Midventricular short-axis	• T2 measured in the interventricular septum at midventricular short-axis.
Ridouani, 2018 [127] (ref. only) 20:20 (40 ± 12)^b^ years Healthy controls, France	• 1.5T, Siemens^a^ (Avanto) • T2-prepared SSFP • Midventricular short-axis and 4-chamber view	• T2 calculated as the average T2 of the entire myocardium of a midventricular short-axis slice and a 4-chamber view.
Granitz, 2019 [131] (ref. only) 26:32 (male: 42 ± 13 years, female: 40 ± 14 years)^b^ Volunteers, Austria	• 1.5T Philips^c^ (Ingenia) • 3T Philips^c^ (Achiva) • T2 GraSE • Basal, midventricular, and apical short-axis	• T2 measured per segment for all 16 segments. • Global T2 calculated as the average of all segments.
Emrich, 2020 [134] (ref. only) 32:26 (50 ± 15)^b^ years Volunteers, Germany	• 3T, Siemens^a^ (Prisma) • T2-prepared SSFP • Two horizontal long-axis and basal, midventricular and apical short-axis	• Global T2 calculated for the entire myocardium of all three short-axis slices.
Boettcher, 2021 [136] (ref. only) 21:29 (39 ± 14)^b^ years Volunteers, Germany	• 1.5T, Siemens^a^ (Avanto) • T2-prepared SSFP • Basal, midventricular, and apical short-axis	• T2 measured per segment for all 16 segments. • Global T2 calculated as the average of all segments.
Graham-Brown, 2021 [137] (ref. only) 40 -^d^ Volunteers, UK	• 3T Siemens^a^ (Skyra) • T2-prepared SSFP • Midventricular short-axis	• T2 calculated for the entire midventricular slice, the interventricular septum, and non-septal segments (for healthy volunteers only T2 of the entire slice reported).
Lin, 2021 [138] (ref. only) 21:21 (44 ± 10)^b^ years Volunteers, China	• 3T, Siemens^a^ (Verio) • T2-prepared SSFP • Midventricular short-axis	• Region of measurement not specified.
Cavus, 2022 [139] 50:79 59 (52-68)^e^ years HCHS, Germany	• 3T, Siemens^a^ (Skyra) • T2-prepared SSFP • Basal, midventricular, and apical short-axis	• T2 measured in the interventricular septum (segment 8) at midventricular short-axis.
Kersten, 2022 [140] (ref. only) 49:26 18-70 years Caucasian volunteers, Germany	• 1.5T, Siemens^a^ (Aera) • T2-prepared SSFP • Midventricular short-axis	• T2 measured for the entire midventricular short-axis slice.
Meloni, 2022 [178] 50:50 20-70 years Caucasian volunteers, Italy	• 1.5T GE^f^ (Signa Artist) • T2 MEFSE • Basal, midventricular, and apical short-axis	• T2 measured per segment for all 16 segments.
Shaw, 2022 [142] (ref. only) 36:14 18-60 years Volunteers, India	• 1.5T, Siemens^a^ (Aera) • Hybrid gradient and spin-echo sequence • Basal, midventricular, and apical short-axis at two different flip angles (12° and 70°)	• T2 measured per segment for all 16 segments.
Snel, 2022 [143] (ref. only) 44:47 18-45 years Volunteers, Netherlands	• 3T, Siemens^a^ (Prisma) • T2-prepared SSFP • Basal, midventricular, and apical short-axis	• T2 measured per segment for all 16 segments (results not shown). • Global T2 calculated as the average of all segments.
Xu, 2023 [145] (ref. only) 515:500 19-87 years Volunteers, China	• 3T, Siemens^a^ (Prisma or Skyra) • T2-prepared FLASH • Basal, midventricular, and apical short-axis, 2-, 3-, and 4-chamber view	• T2 measured per segment for all 16 segments.
Yamagata, 2023 [146] 24:27 19-59 years Volunteers, Malta	• 3T, Siemens^a^ (Vida) • T2-prepared FLASH • Basal, midventricular, and apical short-axis	• T2 measured in the interventricular septum at midventricular short-axis.

*n* number of study subjects, *T* Tesla, *bSSFP* balanced steady-state free precession, *FLASH* fast low-angle shot, *GraSE* gradient echo spin echo, *MEFSE* multi-echo fast-spin-echo, *HCHS* Hamburg City Health study

^a^Siemens Healthineers AG, Erlangen, Germany; ^b^mean ± SD (age range not provided in original publication); ^c^Koninklijke Philips N.V., Amsterdam, The Netherlands; ^d^not provided in original publication; ^e^median (interquartile range) (complete age range not provided in original publication); ^f^GE Healthcare Technologies, Chicago, Illinois, USA

The following tables with reference ranges for T2 time are for guidance only. Due to the various influencing factors described above, site-specific reference ranges must be established in accordance with the current SCMR consensus guidelines [167].

**Table 95:** Normal native myocardial T2-relaxation time (ms) in adults.^a^

Reference	FS	Vendor	Technique	n	Mean ± SD	LL-UL
[178]	1.5	GE^b^	T2 MEFSE	100	52.1 ± 3.1^c^	46-58
[121]	3	Philips^d^	T2 GraSE	46	45 ± 4	37-53
[139]	3	Siemens^e^	T2-preapared bSSFP	129	41 (40-43)^f^	
[146]	3	Siemens^e^	T2-preapared FLASH	51	39.5 ± 1.8	36-43

*Data are means ± standard deviation or median and interquartlie range, as indicated. FS* field strength in Tesla, *n* number of subjects, *SD* standard deviation, *LL* lower limit, *UL* upper limit, *MEFSE* multi-echo fast-spin-echo, *GraSE* gradient spin echo, *bSSFP* balanced steady-state free precession, *FLASH* fast low-angle shot

^a^Measured with a region of interest in the interventricular septum of a midventricular short-axis slice; ^b^GE Healthcare Technologies, Chicago, Illinois, USA; ^c^calculated as the average of segments 8 and 9; ^d^Koninklijke Philips N.V., Amsterdam, The Netherlands; ^e^Siemens Healthineers AG, Erlangen, Germany; ^f^median (first and third quartile) (mean ± SD not provided in original publication)

## 23 Normal myocardial T2-relaxation time in children

### 23.1 Influencing factors

**Gender:** In one study of 9-18 year old children, females had significantly longer (higher) myocardial T2 times than boys [168]. However, another study of adolescents (15-18 years) found no between-sex differences in global T2 relaxation times although T2 time in the mid-ventricular septal segments was slightly longer in boys than in girls [54].

**Table 96:** References. Normal myocardial T2-relaxation time in children.

First author, year n, male:female Age range Study population	CMR acquisition	Post-processing
Barczuk-Falęcka, 2020 [168] 18:20 9-18 years Volunteers, Poland	• 3T, Siemens^a^ (Skyra) • T2 GraSE • Basal, midventricular, and apical short-axis	• T2 measured in the interventricular septum at midventricular short-axis.
Alsaied, 2021 [171] (ref. only) 87:15 5-19 years Patients with pectus excavatum but no cardiac abnormalities	• 1.5T, Philips^b^ (Ingenia) • T2 GraSE • Midventricular short-axis	• T2 measured for the entire midventricular short axis slice.
Real, 2023 [54] 57:61 15-18 years Participants of the EnIGMA project without evidence or history of cardiovascular disease, Spain	• 3T, Philips^b^ (Elition X) • T2 GraSE • Midventricular short-axis	• T2 measured per segment for all 6 segments. • T2 calculated as the average of all 6 segments and septal T1 as the average of segments 8 and 9.

*n* number of study subjects, *T* Tesla, *GraSE* gradient spin echo, *EnIGMA* early imaging markers of unhealthy lifestyles in adolescents

^a^Siemens Healthineers AG, Erlangen, Germany; ^b^Koninklijke Philips N.V., Amsterdam, The Netherlands

**Table 97:** Normal myocardial T2-relaxation time (ms) in children.^a^

Reference	FS	Vendor	Technique	n	Mean ± SD	LL-UL
[168]	3T	Siemens^b^	T2 GraSE	38	43 ± 4.5	34-52
[54]	3T	Philips^c^	T2 GraSE	118	44.8 ± 2.9	39-51

*Data are means ± standard deviation. FS* field strength in Tesla, *n* number of subjects, *LL* lower limit, *UL* upper limit, *GraSE* gradient spin echo

^a^Measured with a region of interest in the interventricular septum of a midventricular short-axis slice; ^b^Siemens Healthineers AG, Erlangen, Germany; ^c^Koninklijke Philips N.V., Amsterdam, The Netherlands

**Age:** One study found a significantly longer myocardial T2 relaxation times in the pubertal period (age 13-15 years) compared to prepubertal years (9-12) [168].

**Anthropometric measures:** There was no significant correlation between T2 times and BMI in children [168].

**Substrates:** (Please see corresponding section for adults.).

**CMR-acquisition:** (Please see corresponding section for adults.).

**Post-processing:** (Please see corresponding section for adults.).

## 24 Normal myocardial T2*-relaxation time in adults

### 24.1 Influencing factors

**Demographic parameters**: T2* of the myocardium is not related to age or gender [180], [189].

**Table 98:** References. Normal myocardial T2*-relaxation time in adults.

First author, year n, male:female Age range Study population	CMR acquisition	Post-processing
Kirk, 2010 [189] 38:25 18-77 years Volunteers, UK	• 1.5T, multi-echo gradient echo sequence with 8 echo times (2.6-16.74 ms) • Mid-ventricular short-axis	• T2* measured with a region of interest in the interventricular septum.
Roy, 2017 [180] 39:36 20-90 years Caucasian volunteers with cardiovascular risk-factors but without other cardiovascular disease, Belgium	• 3T, multi-echo gradient echo sequence with 8 echo times (minimum TE 2.0, echo spacing 2.2 ms) • Mid-ventricular short-axis	• T2* measured with a region of interest in the interventricular septum and the myocardium of the entire slice.

*n* number of study subjects, *T* Tesla, *TE* echo time

**Substrates:** Quantification of the T2* relaxation time plays an important role for estimation of myocardial iron overload [190]. T2* time is also altered in myocardial necrosis and hemorrhage [167].

**CMR acquisition parameters**: For quantification of the myocardial T2* time, the gradient-echo T2* technique with multiple increasing echo times is preferred over the spin-echo T2 technique due to a greater sensitivity to iron deposition [191], [192], [193]. Normal myocardial T2* varies with the field strength [194]. According to the current consensus statement by the SCMR, a dark-blood multi-echo gradient echo sequence with 8 equally spaced echoes between 2 and 18 ms should be used as a single-breath hold technique for T2*-mapping at 1.5T [167]. However, since 1.5T MR machines might not be available at all institutions, normal T2*-values have also been calculated for 3T [180], [194].

**Post-processing**: Gradient-echo T2* images are vulnerable to distortions of the local magnetic field, e.g., by air-tissue interfaces. The myocardial septum is surrounded by blood on both sides, so susceptibility differences are less than in the lateral wall with improved image quality on T2* images. Therefore, T2* measurements are obtained by placing a region of interest on the interventricular septum of a midventricular short-axis slice [167], [193].

T2* times are frequently reported as relaxation rate, representing the reciprocal of the time constant and calculated as R2* = 1000/T2*. The units of R2* is s^−^^1^ [193]. Cardiac iron concentration can be calculated from T2* values by the following equation: [Fe] = 45/(T2*)^1.22^, where [Fe] is the cardiac iron concentration in milligrams per gram dry weight and T2* in milliseconds [195].

Reported normal values of myocardial T2* relaxation time are shown in Table 99. Depending on the risk to develop heart failure as a consequence of myocardial iron overload, a grading system for disease severity has been published for 1.5T and was adapted to 3T (Table 100) [167], [190], [192], [194], [196], [197].

**Table 99:** Normal myocardial T2*-relaxation time.^a^

Reference	n	Field strength		
[189]	63	1.5T	Median (Q1, Q3)	36.3 (31.6, 45.4)
[180]	75	3T	Mean ± SD	25 ± 5

*Data are median and interquartile range or mean ± standard deviation, as indicated. n* number of subjects, *T* Tesla; *Q* quartile, *SD* standard deviation

^a^Measured in the interventricular septum

**Table 100:** Grading of iron overload based on myocardial T2* measurements according to [167], [190], [192], [194], [196], [197].

Iron load	T2* (ms) at 1.5T	T2* (ms) at 3T
Normal	>20	>12
Iron overload	10-20	5.5-12
Severe iron overload	<10	<5.5

*T* Tesla

## 25 Normal values of cardiac strain in adults

### 25.1 Influencing factors

**Gender:** In tagged CMR and some reports of FT-CMR, normal values for LV strain vary by gender. Cardiac strain values for women are higher than those of men [200]. However, some FT-CMR reports showed no association of circumferential or longitudinal strains with age or gender [15].

**Fig. 16:**
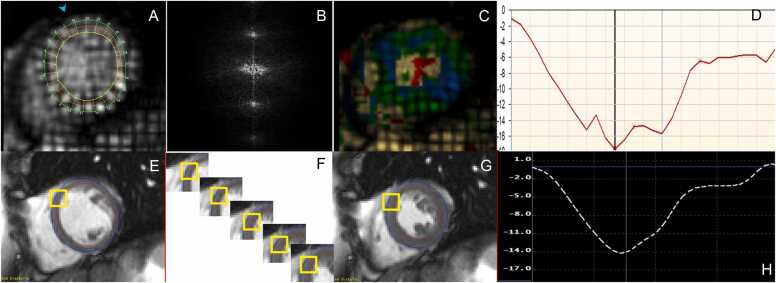
Illustration of strain computation using the Harmonic Phase (HARP) tool on tagged MRI images (A-D) and from feature tracking on cine MRI images (E-H). In HARP, first a semi-automated frequency analysis of the tagged MRI image (A) is performed to identify the harmonic peaks in each of the tag directions (B), filters are then applied to isolate the peaks and obtain the corresponding phase maps from which Eulerian strain maps (C) can be computed. Subplot (D) shows the strain curve at the mid-ventricular level for an asymptomatic volunteer obtained based on tracking of the user-defined mesh (A). In feature tracking of cine MRI images, endo- and epicardial contours are drawn at end-diastole (E) or end-systole (G). A characteristic pixel pattern in the order of a few millimeters squared is identified as a template. The software then tries to discern a similar pattern in the subsequent frame from which displacement of the pixels is computed (F). This is repeated through the entire cycle to obtain displacement from which strain is computed. Subplot (H) shows the strain curve at the mid-ventricular level computed from feature tracking. The tagged and cine MRI images and the strain curves were from the same participant. *MRI* magnetic resonance imaging

**Table 101:** References. Normal values of cardiac strain in adults.

First author, year n, male: female Age range Study population	CMR acquisition	Parameter	Post-processing (Fig. 16)
Augustine, 2013 [198] 54:62 (30 ± 8)^a^ years Volunteers, UK	• 1.5T, short-axis stack, 2-, 3-, and 4-chamber bSSFP	• LV global circumferential and longitudinal strain	• 2D Cardiac Performance Analysis Software (TomTec^b^) was used to obtain strain quantification directly from cine images. • Longitudinal strain was obtained from averaging of 3 long-axis views. Circumferential and radial strain were obtained from 3 short-axis slices of the LV.
Ambale-Venkatesh, 2015 [199] 45:84 45-84 years MESA, USA	• 1.5T, 3 short-axis images, tagged MRI (SPAMM), tagged resolution 7 mm	• LV global circumferential strain	• Short-axis tagged slices were analyzed by the harmonic phase method (HARP v3.0^c^) to assess strain. • Circumferential and radial strain were obtained from 3 short-axis slices.
Andre, 2015 [200] 75:75 21-71 years Volunteers, Germany	• 1.5T, short-axis stack, 2-, 3-, and 4-chamber bSSFP	• LV global circumferential and longitudinal strain	• Image analyses were conducted employing the 2D CPA CMR Feature tracking software (TomTec^b^). • Longitudinal strain was obtained from averaging of 3 long-axis views. Circumferential and radial strain were obtained from 3 short-axis slices of the LV.
Cai, 2017 [77] 91:89 20-69 years Volunteers, Singapore	• 3T, short-axis stack, 2-, 3-, and 4-chamber bSSFP	• LV global circumferential and longitudinal strain	• Myocardial strain and strain rate were measured using cvi42 (Tissue Tracking Plugin).^d^ • Longitudinal strain was obtained from averaging of horizontal and vertical long-axis views. Circumferential and radial strain were obtained from 3 short-axis slices of the LV.
Peng, 2018 [201] 75:75 18-82 years Volunteers, China	• 1.5T and 3T, short-axis stack, 2-, 3-, and 4-chamber bSSFP	• LV global circumferential and longitudinal strain	• Global and regional LA, LV, and RV strain and systolic strain rate measurements were analyzed using commercial cardiovascular post-processing software (Medis 3.0).^e^ • Longitudinal strain was obtained from averaging of horizontal and vertical long-axis views. Circumferential and radial strain were obtained from 3 short-axis slices of the LV.
Liu, 2019 [202] 60:60 20-70 years Volunteers, China	• 1.5T, short-axis stack, 2-, 3-, and 4-chamber bSSFP	• RV global circumferential and longitudinal strain	• The tissue tracking analysis was performed by drawing out the long-axis and the short-axis endo- and epicardial border. The trabecula was excluded, and the tricuspid plane and the apex were marked on the long axis and the short axis of the RV.
Mangion, 2019 [203] Volunteers, 43:45 (45 ± 18)^a^ years Volunteers, Scotland	• 3T, short-axis stack, 2-, 3-, and 4-chamber bSSFP	• LV global circumferential strain, and 2- and 4-chamber longitudinal strain	• Diogenes CMR feature-tracking software (TomTec^b^) was used to quantify strain. • Longitudinal strain was obtained from averaging of 3 long-axis views. Circumferential and radial strain were obtained from 3 short-axis slices of the LV.
Qu, 2020 [204] 75:75 20-80 years Volunteers, Germany	• 1.5T, short-axis stack, 2-, 3-, and 4-chamber bSSFP	• RV global longitudinal strain	• RV free wall longitudinal strain and strain rate were derived from the RV long-axis four chamber view using the commercial specialized software cvi42 (version 5.6.3).^d^ • RV tricuspid annular plane and apex were marked, followed by the manual delineation of the RV endocardial border and free wall epicardial border at end diastole.
Ruijsink, 2020 [205] 929:832 45-74 years UK-Biobank	• 1.5T, short-axis stack, 2-, 3-, and 4-chamber bSSFP	• LV global circumferential strain, and 2- and 4-chamber longitudinal strain	• The myocardial segmentation was performed using a Deep-Learning algorithm. This was followed by a 2D B-spline free-form displacement technique implemented with Medical Image Registration ToolKit to obtain feature-tracking strains.
Gao, 2021 [206] 220:188 21-70 years Healthy participants of CMR screening, China	• 1.5T, short-axis stack, 2-, 3-, and 4-chamber bSSFP	• LA reservoir, conduit, and booster strain	• LA volumetric analysis was performed using commercial software (cvi42, version 5.12.1).^d^ LA endocardial contours were manually traced in the 2- and 4-chamber views excluding pulmonary veins and the LA appendage. • These were then tracked through the cycle to obtain strain.
Qu, 2021 [207] 75:75 20-80 years Volunteers, Germany	• 1.5T, short-axis stack, 2-, 3-, and 4-chamber bSSFP	• LV global circumferential and longitudinal strain	• cvi42 (version 5.3.8)^d^ provided semi-automated delineation of LV endocardium and epicardium at end-diastolic phase on short- and long-axis images. The software automatically propagated the contours and tracked myocardial features phase by phase throughout the cardiac cycle. • Longitudinal strain was obtained from averaging of 3 long-axis views. Circumferential and radial strain were obtained from short-axis slices of the LV.
Li, 2022 [208] 312:254 21-70 years Healthy participants of CMR screening, China	• 1.5T, short-axis stack, 2-, 3-, and 4-chamber bSSFP	• LV global circumferential and longitudinal strain	• cvi42 (version 5.12.1)^d^ was used for manual segmentation of the myocardium. The obtained myocardial contours were then propagated by the software through the cardiac cycle. • Longitudinal strain was obtained from averaging of 3 long-axis views. Circumferential and radial strain were obtained from short-axis slices of the LV.

*n* number of study subjects, *T* Tesla, *bSSFP* balanced steady state free precession, *LV* left ventricular, *MESA* Multi-Ethnic Study in Atherosclerosis, *LA* left atrial, *RV* right ventricular

^a^Mean ± SD (age range not provided in original publication); ^b^TOMTEC Imaging Systems GmbH, Unterschleissheim, Germany; ^c^HARP, Diagnosoft, Palo Alto, California, USA; ^d^Circle Cardiovascular Imaging Inc., Calgary, Alberta, Canada; ^e^Medis Medical Imaging Systems, Leiden, The Netherlands

**Table 102:** Normal strain values (in %) for men and women.

					Men			Women		
Cardiac chamber	Parameter	CMR acquisition	Post-processing/ software	References	n	Mean ± SD	LL-UL	n	Mean ± SD	LL-UL
LV	Global peak circumferential strain	Tagged MRI	HARP^a^	[199]	45	−17.8 ± 1.9	−21.6 to −14.0	84	−18.0 ± 2.5	−23.0 to −13.0
LV	Global peak circumferential strain	bSSFP	2D FT/TT^b^	[198], [200], [203]	172	−21.9 ± 4.4	−30.6 to −13.2	182	−23.7 ± 4.9	−33.3 to −14.1
LV	Global peak longitudinal strain	bSSFP	2D FT/TT^b^	[198], [200], [203]	172	−18.7 ± 3.9	−26.3 to −11.0	182	−21.0 ± 4.1	−29.1 to −12.8
LV	Global peak circumferential strain	bSSFP	2D FT/CVI^c^	[77], [208]	403	−19.2 ± 2.0	−23.1 to −15.2	343	−21.7 ± 2.5	−26.6 to −16.7
LV	Global peak longitudinal strain	bSSFP	2D FT/CVI^c^	[77], [208]	403	−17.0 ± 2.8	−22.5 to −11.6	343	−19.6 ± 3.3	−26.0 to −13.2
LV	Global peak circumferential strain	bSSFP	2D FT/Medis^d^	[201]	75	−23.7 ± 3.1	−29.9 to −17.5	75	−24.9 ± 3.1	−31.1 to −18.7
LV	Global peak longitudinal strain	bSSFP	2D FT/Medis^d^	[201]	75	−21.6 ± 2.5	−26.6 to −16.6	75	−23.3 ± 2.9	−29.1 to −17.5
LV	Global peak circumferential strain	bSSFP	3D FT/CVI^c^	[207]	75	−16.5 ± 2.5	−21.5 to −11.5	75	−17.5 ± 2.8	−23.1 to −11.9
LV	Global peak longitudinal strain	bSSFP	3D FT/CVI^c^	[207]	75	−14.8 ± 2.2	−19.2 to −10.4	75	−16.0 ± 2.3	−20.6 to −11.4
LV	Global peak circumferential strain	bSSFP	MIRTK/2D B-spline FFD^e^	[205]	929	−18.8 ± 2.6	−24.0 to −13.6	832	−20.2 ± 2.8	−25.8 to −14.6
LV	Global peak longitudinal strain, 2 chamber view	bSSFP	MIRTK/2D B-spline FFD^e^	[205]	929	−15.5 ± 5.1	−25.7 to −5.3	832	−17.3 ± 5.7	−28.7 to −5.9
LV	Global peak longitudinal strain, 4 chamber view	bSSFP	MIRTK/2D B-spline FFD^e^	[205]	929	−15.5 ± 5.2	−25.9 to −5.1	832	−15.8 ± 5.8	−27.4 to −4.2
RV	Global peak circumferential strain	bSSFP	2D FT/CVI^c^	[202]	60	−12.3 ± 3.9	−20.1 to −4.5	60	−14.2 ± 4.1	−22.4 to −6.0
RV	Global peak longitudinal strain	bSSFP	2D FT/CVI^c^	[202], [204]	135	−23.5 ± 4.4	−32.2 to −14.8	135	−25.6 ± 5.2	−35.9 to −15.4
LA	Reservoir strain^f^	bSSFP	2D FT/CVI^c^	[206]	220	38.3 ± 8.7	20.9-55.7	188	44.0 ± 9.9	24.2-63.8
LA	Conduit strain^g^	bSSFP	2D FT/CVI^c^	[206]	220	22.3 ± 6.8	8.7-35.9	188	26.7 ± 8.0	10.7-42.7
LA	Booster strain^h^	bSSFP	2D FT/CVI^c^	[206]	220	16.0 ± 3.8	8.4-23.6	188	17.3 ± 4.4	8.5-26.1

*Data are means ± standard deviation. n* number of study subjects, *LL* lower limit, *UL* upper limit, *LV* left ventricle, *HARP* harmonic phase, *FT* feature tracking, *bSSFP* balanced steady-state free precession, *FFD* Free-Form Deformation, *RV* right ventricle, *LA* right atrium

^a^HARP commercial, Diagnosoft, Palo Alto, California, USA; ^b^TT = TomTec Imaging Systems, Unterschleissheim, Germany; ^c^CVI = CVI42, Circle Cardiovascular Imaging Inc., Calgary, Alberta, Canada; ^d^Medis = Medis Medical Imaging Systems, Leiden, The Netherlands; ^e^MIRTK = Medical Image Registration ToolKit; ^f^corresponds to atrial reservoir function during left-ventricular end-systole; ^g^corresponds to atrial conduit function during early diastole; ^h^corresponds to atrial booster function during late diastole

References [199], [207] provided additional data for analysis.

**Age:** Using both tagged CMR and FT-CMR, several studies report greater age is associated with decrease in peak circumferential or longitudinal shortening [201].

**Ethnicity:** A few studies have shown that the reference ranges are different for different race/ethnic groups. In MESA, African-Americans had reduced circumferential shortening compared to Chinese-Americans, with Hispanics and Caucasians in the intermediate range [199]. The UK Biobank showed that South Asians had on average greater circumferential and longitudinal strain compared to White European participants [209].

**Left ventricular volumes and function:** In MESA, better LV circumferential strain (more negative) was associated with greater ejection fraction, lower LV mass, and smaller LV volumes [210].

**Post-processing:** With tagged MRI, normal midwall circumferential strain values are relatively comparable between studies [199], [211]. Myocardial deformation assessed by 2D FT-CMR depends on segmentation procedure and type of analysis software, small differences between published results exist for reference values, probably due to inter-vendor differences [77], [200]. Moreover, for both tagged MRI and FT-CMR, attention should be paid to the number of short-axis slices used for calculating global circumferential and radial strains. Most publications use three slices, one each at the levels of base, mid-ventricle, and apex. Longitudinal strain is typically averaged over two-, three-, and four-chamber images.

**Acquisition sequences:** The reference ranges of normal circumferential strains from FT-CMR are seen to be comparable to those obtained from tagged CMR for circumferential strain.

## 26 Normal values of cardiac strain in children

### 26.1 Influencing factors

**Gender:** LV, LA, and RA measurements did not differ significantly between boys and girls. RV strain was higher in girls as compared to boys [212].

**Table 103:** References. Normal values of cardiac strain in children.

First author, year n, male:female Age range Study population	CMR acquisition	Parameter	Post-processing
Voges, 2022 [212] 102:55 5-18 years Patients with CMR for clinical indication but without cardiovascular pathology at CMR and clinical follow-up	• 1.5T, short-axis stack and 2-, 3-, and 4ch bSSFP	• LV, RV, LA, and RA global longitudinal strain; LV global circumferential strain	• Global LV, RV, LA, and RA strain were analyzed using commercial cardiovascular post-processing software (Medis^a^). • LA longitudinal strain was obtained from averaging of horizontal and vertical long-axis view; LV longitudinal strain from the average of 2-, 3- and 4-chamber views; RV and RA longitudinal strain from one 4-chamber view; circumferential strain was obtained from three short-axis slices of the LV.

*n* number of study subjects, *T* Tesla, *2ch* two-chamber view, *3ch* three-chamber view, *4ch* four-chamber view, *LV* left ventricular, *RV* right ventricular, *LA* left atrial, *RA* right atrial

^a^Medis Medical Imaging Systems, Leiden, The Netherlands

**Table 104:** Centiles of normal global left ventricular myocardial longitudinal strain (in %) in children by body height according to reference [212].

Height (cm)	5^th^	10^th^	25^th^	50^th^	75^th^	90^th^	95^th^
100	−30.5	−29.1	−26.9	−24.5	−22.0	−19.8	−18.5
110	−29.7	−28.4	−26.3	−23.9	−21.6	−19.5	−18.2
120	−28.9	−27.7	−25.6	−23.4	−21.1	−19.1	−17.9
130	−28.1	−26.9	−25.0	−22.8	−20.6	−18.7	−17.5
140	−27.3	−26.2	−24.3	−22.2	−20.2	−18.3	−17.2
150	−26.6	−25.5	−23.7	−21.7	−19.7	−17.9	−16.8
160	−25.8	−24.8	−23.0	−21.1	−19.2	−17.5	−16.4
170	−25.1	−24.1	−22.4	−20.6	−18.7	−17.1	−16.1
180	−24.3	−23.4	−21.8	−20.0	−18.2	−16.6	−15.7
190	−23.6	−22.7	−21.1	−19.4	−17.7	−16.2	−15.3
200	−22.9	−22.0	−20.5	−18.9	−17.3	−15.8	−14.9

**Table 105:** Centiles of normal global left ventricular myocardial circumferential strain (in %) in children by age according to reference [212].

Age (y)	5^th^	10^th^	25^th^	50^th^	75^th^	90^th^	95^th^
4	−28.7	−27.1	−24.5	−21.6	−18.7	−16.0	−14.5
5	−28.4	−26.9	−24.4	−21.7	−18.9	−16.4	−14.9
6	−28.2	−26.7	−24.4	−21.8	−19.1	−16.8	−15.4
7	−27.9	−26.6	−24.3	−21.8	−19.3	−17.1	−15.7
8	−27.6	−26.3	−24.2	−21.8	−19.5	−17.3	−16.0
9	−27.3	−26.1	−24.1	−21.8	−19.5	−17.5	−16.2
10	−27.0	−25.8	−23.8	−21.7	−19.5	−17.5	−16.4
11	−26.6	−25.5	−23.6	−21.5	−19.4	−17.5	−16.4
12	−26.3	−25.2	−23.3	−21.3	−19.2	−17.4	−16.3
13	−26.0	−24.9	−23.1	−21.0	−19.0	−17.1	−16.0
14	−25.8	−24.7	−22.8	−20.7	−18.6	−16.7	−15.6
15	−25.5	−24.4	−22.4	−20.3	−18.2	−16.2	−15.1
16	−25.3	−24.1	−22.1	−19.8	−17.6	−15.6	−14.4
17	−25.0	−23.8	−21.7	−19.3	−17.0	−14.9	−13.6
18	−24.8	−23.5	−21.3	−18.8	−16.4	−14.2	−12.8

**Table 106:** Normal right ventricular and atrial strain (in %) in children according to reference [212].

Cardiac chamber	Parameter	Male (mean ± SD)	Female (mean ± SD)
Right ventricle	Endocardial global longitudinal strain	−24.6 ± 4.3	−26.2 ± 4.3
Left atrium	Endocardial global longitudinal strain, 4ch	31.8 ± 8.0	30.9 ± 9.7
Left atrium	Endocardial global longitudinal strain, 2ch	40.4 ± 11.2	37.9 ± 11.8
Right atrium	Endocardial global longitudinal strain	25.5 ± 8.2	27.1 ± 8.1

*Data are means ± standard deviation. 4ch* four-chamber view, *2ch* two-chamber view

**Age:** Large variations are seen across neonates and infants as there are significant histological and physiological changes that happen early in life. Longitudinal and circumferential strains decrease with age [212].

**Anthropometric measures:** Ventricular strains are strongly dependent on body size with both increase in height and weight leading to decreased longitudinal and circumferential strains. Atrial strains were however independent of body size [212].

**Post-processing:** Myocardial deformation assessed by 2D FT-CMR depends on segmentation procedure and type of analysis software, small differences between published results exist for reference values, probably due to inter-vendor differences [77], [200].

## 27 Normal values of myocardial perfusion in adults

### 27.1 Influencing factors

**Gender:** Rest and stress myocardial blood flow (MBF), including rest MBF normalized by the rate-pressure product, is higher in females (before menopause) than males [214], [215].

**Fig. 17:**
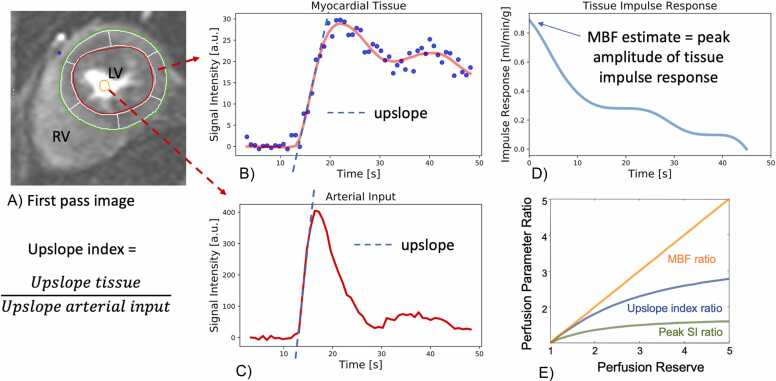
(A) The quantification of myocardial perfusion proceeds from the segmentation of images acquired during the first pass of contrast through the heart to delineate myocardial segments and a region in the center of the LV blood pool for the arterial input. This example shows one short-axis image for a mid-slice level in the left ventricle. (B) For each myocardial segment, one obtains a signal-intensity versus time curve. A useful semi-quantitative parameter for the assessment of the perfusion in a myocardial segment is the upslope, which is estimated from a fit to approximately 3-5 points during the initial myocardial contrast enhancement. (C) An analogous upslope parameter can be extracted from the first pass peak of the arterial input function. A perfusion index can be calculated from the ratio of the two upslopes as shown in the formula below panel (A), and accounts for some changes in the arterial input between rest and stress. (D) Absolute estimates of myocardial blood flow in mL/min/g can be obtained from the myocardial contrast enhancement curves and the arterial input function by fitting to a kinetic model for contrast enhancement, or, as done for this example, to estimate the myocardial impulse response by constrained deconvolution. Constraints are that the impulse response should be a monotonically decaying function of time, and requiring a relatively smooth, “regularized” impulse response. Myocardial blood flow (MBF) is estimated from the peak amplitude of the impulse response. (E) The ratio of myocardial blood flows during stress, divided by MBF at rest, provides the most accurate estimate of the coronary flow reserve. In comparison, other ratios of perfusion indices (e.g., upslope index) for stress and rest systematically underestimate the flow reserve but may still prove useful for the detection of disease, assuming that one has established the normal range of the index. *LV* left ventricular

**Table 107:** References. Normal values of myocardial perfusion in adults.

First author, year n, male: female Age range Study population	CMR acquisition	Post-processing (Fig. 17)
*Dipyridamole*		
Vasu, 2013 [213] 14:1 21 (3, 5) years^a^ Volunteers, USA	• 1.5T, T1-weighted saturation-recovery prepared with single-shot per slice 2D bSSFP image read-out (TR/TE/flip = 2.3/1.1 ms/50°) • 1 R-to-R temporal resolution; 60 dynamics/slice for rest and dipyridamole stress (4 min after infusion)	• Signal-intensity curves for 6 myocardial sectors/slice and ROI in center of LV cavity for arterial input function. • Proton-density image for receiver coil-profile correction. • Fermi-model based deconvolution of myocardial curves with arterial input for quantification in mL/min/g.
*Adenosine*		
Wang, 2006 [214] 49:50 59 ± 11 years^b^ MESA, USA	• 1.5T, T1-weighted saturation recovery prepared, with single-shot per slice 2D gradient echo image-read-out • 1 R-to-R temporal resolution; 60 dynamics/slice for rest and stress	• Signal-intensity curves for 6 myocardial sectors/slice and ROI in center of LV cavity for arterial input function. • Receiver coil-profile correction from pre-contrast images. • Fermi-model based deconvolution of myocardial curves with arterial input for quantification in mL/min/g.
Brown, 2023 [215] 88:62 19-79 years Volunteers, UK	• 3T, dual-contrast T1-weighted saturation recovery prepared, with single-shot per slice 2D gradient echo image-read-out • 1 R-to-R temporal resolution; 60 dynamics/slice for rest and stress (140 μg/kg/min for minimum of 3 min) • First three beats, proton density weighted images for coil profile correction and signal calibration	• AI-based automatic segmentation of myocardium. • Breathing motion correction and elastic deformation-based image registration. • Quantification of myocardial blood flow with blood-tissue model of contrast exchange.
*Regadenoson*		
Vasu, 2013 [213] 14:1 21 (3, 5) years^a^ Volunteers, USA	• As above for Vasu, 2013 • Regadenoson injected ~70 s min before start of image acquisition.	• As above for Vasu, 2013.

*n* number of study participants, *T* Tesla, *bSSFP* balanced steady-state free precession, *TR* relaxation time, *TE* echo time, *MESA* Multi-Ethnic Study of Atherosclerosis

Reference [214] provided additional data (analysis of a subset of healthy subjects without hypertension, no use of antihypertensive or other medication for a cardiovascular condition, no diabetes, normal glucose tolerance, no smoking history and normal total cholesterol [<240 mg/dL] of the original cohort)

^a^Median, interquartile range (age range not provided in original publication), ^b^mean ± SD (age range not provided in original publication)

**Table 108:** Normal absolute myocardial blood flow at rest and during stress and perfusion reserve in adults.

				MBF at rest (mL/min/g)		MBF during stress (mL/min/g)		Perfusion reserve^a^	
Reference	Stress agent	n	Sample	Mean ± SD	LL-UL	Mean ± SD	LL-UL	Mean ± SD	LL-UL
[214]	Adenosine	99 49 50	All Men Women	1.02 ± 0.24 0.96 ± 0.23 1.08 ± 0.23	0.54-1.50 0.50-1.42 0.62-1.54	3.13 ± 0.80 2.79 ± 0.72 3.46 ± 0.73	1.53-4.73 1.35-4.23 2.00-4.92	3.17 ± 0.87	1.43-4.91
[213]	Dipyridamole	15	All	1.09 ± 0.22		2.81 ± 0.67		2.61 ± 0.57	
[213]	Regadenoson	15	All	1.21 ± 0.38		3.58 ± 0.58		3.11 ± 0.62	
[215]	Adenosine	150 88 62	All Men Women	0.62 ± 0.13 0.58 ± 0.12 0.69 ± 0.13	0.36-0.88 0.34-0.82 0.34-0.95	2.24 ± 0.53 2.13 ± 0.54 2.41 ± 0.47	1.18-3.30 1.05-3.21 1.47-3.35	3.74 ± 1.0 3.79 ± 1.0 3.67 ± 1.0	1.74-5.74 1.79-5.79 1.67-5.67

*Data are means ± standard deviation. MBF* myocardial blood flow, *n* number of study participants, *SD* standard deviation, *LL* lower limit, *UL* upper limit

^a^Ratio of MBF during stress divided by MBF at rest

**Age:** Higher age, a risk factor for atherosclerosis, is associated with a reduction of hyperemic blood flow, but the available data do not allow to conclusively determine if this age-related decline is sex-dependent [214], [215].

**Anthropometric measures:** No known association of BSA with rest or stress MBF, unless it involves obese subjects.

**CMR acquisition:** The magnetic field strength of the scanner does not appear to be a confounding factor. Image read-outs with steady-state free precession are sensitive to transient shifts of the resonance frequency as a bolus of contrast passes through the heart. This can result in signal modulation for steady-state free precession read-outs that are sensitive to off-resonance shifts. At 3T, the resonance frequency shifts lead to prominent image artifacts. Use of a surface coil introduces a spatial variation of the signal intensity, which needs to be corrected [216].

**Post-processing:** The MBF estimates depend on the method used for quantification of blood flow. Early studies did not use a saturation-correction for the arterial input, and instead relatively low gadolinium contrast dosages (~0.03 mmol/kg) were injected, which nevertheless can still cause saturation of the peak signal intensity. In general, saturation effects result in a bias to over-estimate myocardial blood flow [217], [218]. Saturation effects are more pronounced with normal cardiac output, resulting in a compact first pass, compared to reduced cardiac output when the first pass of contrast in the ventricular cavity is more dispersed.

## 28 Normal values of myocardial perfusion in children

### 28.1 Influencing factors

**Gender and Age:** Gender and age are not known to have any effect on rest and stress myocardial blood flow in children.

**Table 109:** References. Normal values of myocardial perfusion in children.

First author, year n, male:female Age range Study population	CMR acquisition	Post-processing
Madriago, 2015 [219] 11:9 0.3-16 years Children with hemodynamically trivial congenital heart malformations	• 1.5T, T1-weighted saturation-recovery prepared with single-shot per slice 2D bSSFP image read-out (TR/TE/flip = 3/1.4 ms/20°) • 1 R-to-R temporal resolution; 60 dynamics/slice for rest and stress (140 μg/kg/min for 3 min)	• Signal-intensity curves for 6 myocardial sectors/slice and ROI in center of LV cavity for arterial input function. • Receiver coil-profile correction from pre-contrast images. • Fermi-model based deconvolution of myocardial curves with saturation-corrected arterial input for quantification in mL/min/g.

*n* number of study participants, *T* Tesla, *bSSFP* balanced steady-state free precession, *TR* relaxation time, *TE* echo time

Reference [219] provided additional data for analysis

**Table 110:** Normal absolute myocardial blood flow at rest and during stress and perfusion reserve in children.

Reference	Stress agent	n	MBF at rest (mL/min/g) Mean ± SD	MBF during stress (ml/min/g) Mean ± SD	Perfusion reserve^a^ Mean ± SD
[219]	Adenosine	20	0.94 ± 0.17	2.34 ± 0.82	2.63 ± 0.96

*Data are means ± standard deviation. n* number of study participants, *MBF* myocardial blood flow, *SD* standard deviation

^a^Ratio of MBF during stress divided by MBF at rest

**BSA:** No known effect.

**CMR acquisition:** Similar considerations as for adults apply here.

## 29 Normal myocardial triglyceride content in adults

### 29.1 Influencing factors

**Gender:** No consistent impact of gender on myocardial triglyceride has been demonstrated [221], [223].

**Fig. 18:**
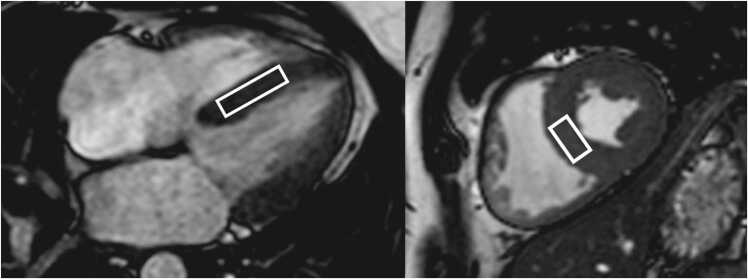
Position of the ^1^H-MRS voxel within the interventricular septum for determination of myocardial triglyceride ratio. From Reference [222]. *MRS* magnetic resonance spectroscopy

**Table 111:** References. Normal myocardial triglyceride content in adults.

First author, year n, male: female Age range Study population	CMR acquisition	Post-processing (Fig. 18)
Rijzewijk, 2008 [220] 28:0 45-65 years Volunteers, The Netherlands	• 1.5T, Philips^a^ • ECG- and navigator-gated • Water suppressed spectra acquired at end-systole (echo time 26 ms, repetition time at least 3000 ms)	• Myocardial proton magnetic resonance spectroscopy spectra were fitted using in-house custom JAVA-based software. • Myocardial triglyceride content was determined as a percentage relative to water as (signal amplitude of triglyceride)/(signal amplitude of water) × 100.
Petritsch, 2016 [221] 22:18 32 ± 8 years (men)^b^ 22 ± 11 years (women)^b^ Volunteers, Germany	• 3T, Siemens^c^ • ECG- and navigator-gated • Single voxel proton magnetic resonance spectroscopy performed at end-systole and end-expiration using a point-resolved spectroscopy sequence (echo time 35 ms, repetition time of at least one heartbeat). • A set of 32 single data acquisitions was averaged for each spectrum.	• Data were analyzed using commercially available software (Spectroscopy Evaluation, Siemens Healthcare, Erlangen, Germany). • Relative myocardial triglyceride was calculated by expressing the myocardial metabolite signal as a percentage of the unsuppressed water signal (myocardial triglyceride/water resonance ratio).
Gastl, 2019 [222] 8:3 63 ± 9 years^b^ Volunteers, Switzerland	• 1.5T, Philips^a^ • ECG- and navigator-gated single voxel, point resolved spectroscopy sequence (PRESS) • Voxel placed in the interventricular septum	• Myocardial proton magnetic resonance spectroscopy spectra were processed offline using in-house custom software. • The triglyceride/water ratio was calculated as the sum of the fitted triglyceride resonance at 0.9 and 1.3 ppm divided by the fitted water.
Bakermans, 2021 [223] 8:8 31 ± 3 years (men)^b^ 28 ± 2 years (women)^b^ Volunteers, The Netherlands	• 3T, Philips^a^ • ECG- and navigator-gated single voxel, point‐resolved spectroscopy sequence (PRESS) • Voxel placed in the interventricular septum	• Spectral fitting was performed using in-house custom software. • Myocardial triglyceride content was estimated as the percentage of the sum of the triglyceride-methylene and triglyceride-methyl signal amplitudes divided by the unsuppressed water signal amplitude.
Soghomonian, 2023 [224] 96:112 21 [20; 24]^d^ Volunteers, Europe (NS)	• 3T, Siemens^c^ • ECG-gated, single voxel, point resolved spectroscopy sequence (PRESS) • Voxel placed in the interventricular septum on cine images	• Myocardial proton magnetic resonance spectroscopy spectra were processed offline using in-house custom software. • Myocardial triglyceride content was determined as a percentage of tissue water content (%triglyceride = triglyceride/water) × 100) with the resonance of triglycerides and water integrated in the frequency domain and taking into account the saturation due to incomplete relaxation.

*n* number of study subjects, *T* Tesla, *NS* not specified

^a^Koninklijke Philips N.V., Amsterdam, The Netherlands; ^b^Siemens Healthineers AG, Erlangen, Germany; ^c^mean ± standard deviation (age range not provided in original publication); ^d^median [25th percentile; 75th percentile] (age range not provided in original publication)

**Table 112:** Normal myocardial triglyceride content (in %) in adults.

Reference	Field strength	n	Mean ± SD
[220]	1.5T	28	0.65 ± 0.05
[221]	3T	40	0.41 ± 0.47
[222]	1.5T	11	0.80 ± 0.26
[223]	3T	16	0.35 ± 0.13
[224]	3T	208	0.42 [0.23; 0.67]^a^

*Data are means ± standard deviation or median and 25th ad 75th percentile, as indicated. n* number of study subjects, *SD* standard deviation, *T* Tesla

^a^Median [25^th^; 75^th^ percentile] (according to original publication)

**Age:** Greater age is associated with higher myocardial triglyceride ratio (0.1% per 10 years) [225].

**Obesity/diabetes:** Obesity and diabetes are associated with higher myocardial triglyceride ratio [220], [224], [226], [227], [228].

**CMR acquisition parameters:** ^1^H-MRS spectral resolution and signal is superior at 3T versus 1.5T. As a result, 3T MRS results are expected to be more reliable than at 1.5T. For ^1^H-MRS of the heart, a point resolved spectroscopy (PRESS) sequence is typically used to restrict signal acquisition to the interventricular myocardial septum. ^1^H-MRS acquisitions are typically both respiratory triggered (navigator echo) and ECG-gated due to long acquisition times (10-15 min). Patients are typically asked to fast for more than 2 h before the procedure.

Repetition times are 1-2 heart beats; echo times range from 20-35 ms. Acquisitions are obtained with and without water suppression to quantify the myocardial triglyceride resonance. Multiple signal averages are used to improve signal-to-noise ratio. The voxel size is 6-8 mm.

**CMR analysis methods:** The signal contribution of protons in methylene groups (1.3 ppm) and methyl groups (0.9 ppm) are summed to represent triglyceride signal. This is divided by proton water signal (4.7 ppm) to obtain the myocardial triglyceride ratio.

The detailed analysis of myocardial triglyceride is complex and involvement of an experienced MR spectroscopist is encouraged to oversee the data evaluation. Analysis of the myocardial triglyceride spectra is not standardized, but should include fitting for both longitudinal and transverse relaxation as well as other curve fitting assumptions that cannot be readily validated [229]. As a result, caution is warranted when comparing reported lipid levels in the literature.

## Conclusions

This review provides an updated and expanded version of reference ranges for routine and specific quantitative cardiovascular imaging parameters to be applied in clinical practice and research.

Continuing publication of normal values of large reference cohorts using new acquisition and post-processing techniques require periodical updates of existing reference ranges.

## Declaration of Generative AI and AI-assisted technologies in the writing process

The authors confirm that they made no use of generative AI and AI-assisted technologies in the writing process.

## Declaration of competing interests

David Bluemke reports a relationship with GE Healthcare that includes consulting or advisory. Co-author editor for the Journal of Cardiovascular Magnetic Resonance: Christopher François. The other authors declare that they have no known competing financial interests or personal relationships that could have appeared to influence the work reported in this paper.
